# Steroidal Glycosides from *Allium tuberosum* Seeds and Their Roles in Promoting Testosterone Production of Rat Leydig Cells

**DOI:** 10.3390/molecules25225464

**Published:** 2020-11-22

**Authors:** Da-Bing Zhang, Xian-Yong Wei

**Affiliations:** 1 Key Laboratory of Coal Processing and Efficient Utilization, Ministry of Education, China University of Mining & Technology, Xuzhou 221116, Jiangsu, China; dbzhang@sina.cn; 2 Jiangsu Hanbon Science & Technology Co., Ltd., Huaian 223005, Jiangsu, China; 3 State Key Laboratory of High-efficiency Coal Utilization and Green Chemical Engineering, Ningxia University, Yinchuan 750021, Ningxia, China

**Keywords:** liliaceae, *Allium tuberosum*, steroidal saponin, allituberoside

## Abstract

A systematic phytochemical study on the components in the seeds of *Allium tuberosum* was performed, leading to the isolation of 27 steroidal glycosides (SGs **1**–**27**). The structures of SGs were identified mainly by nuclear magnetic resonance and mass spectrometries as well as the necessary chemical evidence. In the SGs, **1**–**10** and **22**–**26** are new steroidal saponin analogues. An in vitro bioassay indicates that **1**, **2**, **7**, **8**, **10**, **13**–**15**, **20**, **23**, and **26** display promotional roles in testosterone production of rat Leydig cells with the EC_50_ values of 1.0 to 4.5 μM, respectively.

## 1. Introduction

Steroidal saponins (SSs) are the important class of secondary metabolites in many medicinal plants. Structurally, SSs are mostly in the form of glycosides which are composed of one or more hydrophilic sugar residues and hydrophobic steroidal part [1]. All the time, they have been a kind of constituents concerned by scholars owing to the wide range of their biological actions. For example, SSs from *Dioscorea zingiberensis* are widely used for preventing cardiovascular diseases [2], SSs of *Ophiopogon japonicus* displayed multiple biofunctions of anticancer, immunomodulation, anti-oxidation, anti-inflammation, and anti-diabetes [3]; SSs in Paris species are used to treat cancer and bleeding [4], and timosaponin AIII obtained from *Anemarrhena asphodeloides* exhibits inhibitory activity against tumor cells [5].

**Figure 1:**
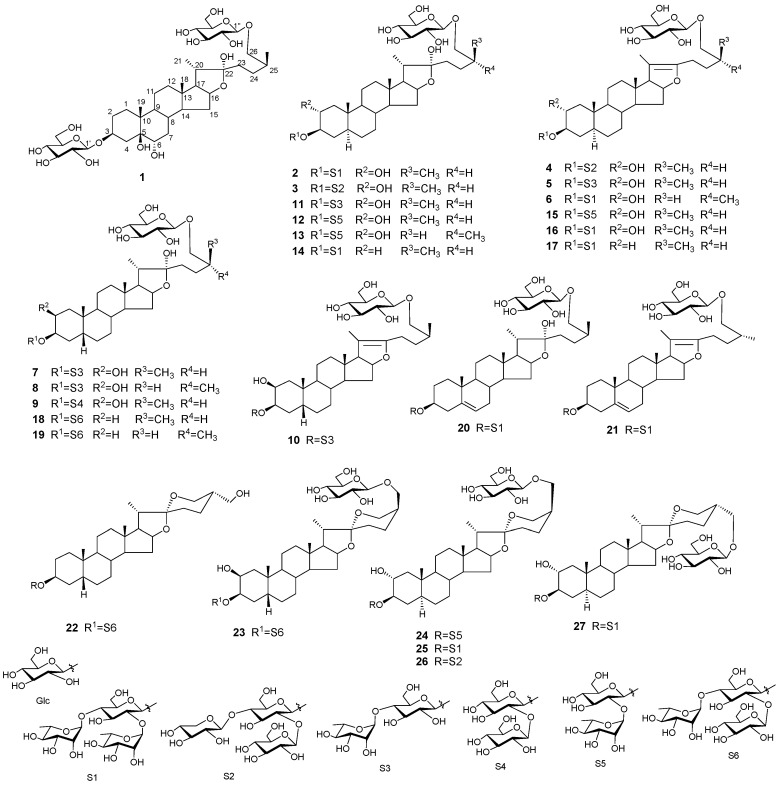
Structures of **1**–**27.**

SSs have been reported in more than 40 different Allium species [6]. *Allium tuberosum* is a type of Allium plant widely cultivated as food in China, and the mature seeds of this plant are used as a traditional herb medicine treating both impotence and nocturnal emissions [7]. The seeds of this plant are famous for their sulfur-containing biologically active natural products [8,9,10], and they also contain amounts of SSs as the main constituents [11,12,13,14,15,16,17]. Previous studies reported many SSs from *A. tuberosum* seeds (ATSs), while a systematic phytochemical investigation for clarifying the bioactive SSs is still necessary. Recently, a systematic phytochemical study on the components in ATSs finally let us to obtain total 27 SSs (**1**–**27**) (Figure 1). According to the traditional pharmacological action of ATSs, all isolated compounds were tested for their effects on testosterone production of rat Leydig cells.

## 2. Results and Discussion

By comparing the nuclear magnetic resonance (NMR) data with the reported values, the known compounds **11**–**18**, **20**, **21**, and **27** are identified as trigofoenoside B (**11**) [18], trigoneoside Xa (**12**) [19], trigoneoside Xb (**13**) [19], nicotianoside F (**14**) [20], tuberoside A (**15**) [11], tuberoside B (**16**) [11], 26-*O*-*β*-d-glucopyranosyl-(25*S*)-5*α*-furostan-△^20(22)^-ene-3*β*,26-glycol-3-*O*-*α*-l-rhamnopyranosyl-(1→4)-[*α*-l-rhamnopyranosyl(1→2)]-*β*-d-glucopyranoside (**17**) [21], shatavarin I (**18**) [22], protoneodioscin (**20**) [23], pseudoprotodioscin (**21**) [24], tuberoside L (**27**) [13], respectively. The structure of **19** has been already registered in CAS with the numbers of 1493828-40-2 and also reported as 3-*O*-*α*-l-rhamnopyranosyl(1→4)-[*β*-d-glucopyranosyl(1→2)]-*β*-d-glucopyranosyl-26-*O*-*β*-d-glucopyranosyl-(25*R*)-5*β*-furostane-3*β*,22*α*,26-triol in the literature [25], but no full NMR data are available for them. By systematic spectroscopic data analysis, its NMR data are assigned (see Supplementary Materials). The remaining new analogues are identified mainly by analysis of NMR and MS spectra as well as the necessary chemical evidence such as acid hydrolysis experiments for determining absolute configuration of the sugar units in the structures.

Compound **1** is in the form of white amorphous powders with a molecular formula of C_39_H_66_O_16_ as determined by HR-ESI-MS at *m*/*z* 789.4272 [M − H]^−^ (calcd for C_39_H_65_O_16_, 789.4273) together with its ^13^C NMR data (Table 1). In the ^1^H NMR spectrum, four typical methyl proton signals at *δ* 0.88 (3H, s), 1.17 (3H, s), 1.33 (3H, d, *J* = 6.8 Hz), 1.05 (3H, d, *J* = 7.0 Hz), and two anomeric proton signals at *δ* 4.83 (1H, d, *J* = 7.8 Hz) and 5.11 (1H, d, *J* = 7.8 Hz) are observed. Its ^13^C NMR spectrum exhibits total 39 carbon resonances including 27 ones due to the aglycone part and twelve ones attributed to the two hexoses. Comparison of NMR data suggests that **1** has the identical planar structure of aglycone skeleton with 26-*O*-*β*-d-glucopyranosyl-(25*S*)-3*β*,5*β*,6*α*,22,26-pentahydroxyl-5*β*-furostane 3-*O*-*α*-l-rhamnopyranosyl-(1→4)-*β*-d-glucopyranoside [17], which is further confirmed by detailed analyses of ^1^H-^1^H COSY, HSQC, and HMBC spectra of **1**. The chemical shifts of H-26a (*δ* 4.09) and H-26b (*δ* 3.50) (Δab > 0.57) further confirm the C-25*S* configuration of **1 [26]**. Starting from the anomeric protons at *δ* 4.83 (H-1′) and 5.11 (H-1′′), the proton signals of sugars are delineated relying on the ^1^H-^1^H COSY correlations, contributing to establish the structures of the two sugar unites. The large coupling constants of *J*_1′, 2′_ (7.8 Hz) and the ^13^C NMR data facilitate defining the sugars as *β*-d-glucopyranoses, which is also supported by the result of the acid hydrolysis experiment. The connectivity of the glucopyranose is identified by the HMBC correlations of *δ* 4.83 (H-1′)/79.2 (C-3), and *δ* 5.11 (H-1′′)/75.4 (C-26), respectively. Consequently, the structure of **1** is elucidated as 26-*O*-*β*-d-glucopyranosyl-(25*S*)-furost-3*β*,5*β*,6*α*,22*α*,26-pentanol 3-*O*-*β*-d-glucopyranoside, named allituberoside A.

Compound **2** has a molecular formula of C_51_H_86_O_23_ as determined by HR-ESI-MS. Its ^1^H NMR spectrum obviously shows four typical methyl proton signals at *δ* 0.87 (3H, s), 0.90 (3H, s), 1.05 (3H, d, *J* = 7.0 Hz), and 1.31 (3H, d, *J* = 6.8 Hz), and four anomeric proton signals at *δ* 4.82 (1H, d, *J* = 7.9 Hz), 5.03 (1H, d, *J* = 7.1 Hz), 5.86 (1H, br s), and 6.40 (1H, br s). Its ^13^C NMR spectrum exhibits total 45 carbon resonances including 27 ones due to the aglycone part and 24 ones attributed to the sugar moieties consisting of four hexoses. The ^13^C NMR data of **2** suggests that it had the identical aglycone skeleton with **11** and the same sugar moieties with **14**, which is supported by its ^1^H-^1^H COSY, HSQC, and HMBC spectral data. The chemical shifts of H-26a (*δ* 4.10) and H-26b (*δ* 3.49) (Δab > 0.57) further confirm the C-25*S* configuration of **2**. Thus, the structure of **2** is elucidated as 26-*O*-*β*-d-glucopyranosyl-(25*S*)-5*α*-furost-2*α*,3*β*,22*α*,26-tetrol 3-*O*-α-l-rhamnopyranosyl -(1→4)-[*α*-l-rhamnopyranosyl-(1→2)]-*β*-^D^-glucopyranoside, named allituberoside B.

Compound **3** displays a molecular formula of C_50_H_84_O_23_ as confirmed by HR-ESI-MS. Its NMR data suggests that **3** has the identical structure with **2** except for the terminal sugar unit at C-4′. The neutral missing fragment of 136 Da presented on the mass spectrum of **3** suggests that **3** has a xylose moiety, and detailed analyses of ^1^H-^1^H COSY, HSQC, and HMBC spectra finally identified the structure of the C-3 sugar chain of **3** to be 3-*O*-*β*-d-xylopyranosyl-(1→4)-[*α*-l-rhamnopyranosyl-(1→2)]-*β*-d-glucopyranoside. The chemical shifts of H-26a (*δ* 4.11) and H-26b (*δ* 3.49) (Δab > 0.57) further confirm the C-25*S* configuration of **3**. Therefore, the structure of **3** is elucidated as 26-*O*-*β*-d-glucopyranosyl-(25*S*)-5*α*-furost-2*α*,3*β*,22*α*,26-tetrol 3-*O*-*β*-d-xylopyranosyl-(1→4)-[*α*-l-rhamnopyranosyl-(1→2)]-*β*-d-glucopyranoside, named allituberoside C.

Compound **4** has a molecular formula of C_50_H_82_O_22_ as determined by HR-ESI-MS. Its NMR data suggests that the structure of **4** is closely similar to that of **3** except for the different substructure surrounding C-20 and C-22 positions. In its ^13^C NMR spectrum, the characteristic carbon signals of *δ* 103.6 and 152.4 which are the same as those of **15**–**17** indicate the existence of the Δ^20(22)^-ene substructure in **4**, which is supported by the HMBC correlations of *δ* 1.62 (H-21)/103.6 (C-20) and 152.4 (C-22). By detailed analysis of ^1^H-^1^H COSY, HSQC, and HMBC spectra, the structure of **4** is further confirmed, and its C-25*S* configuration is deduced according to the chemical shifts of H-26a (*δ* 4.10) and H-26b (*δ* 3.49) (Δab > 0.57). Subsequently, the structure of **4** is elucidated as 26-*O*-*β*-d-glucopyranosyl-(25*S*)-5*α*-furost-Δ^20(22)^-ene-2*α*,3*β*,26-triol 3-*O*-*β*-d-xylopyranosyl-(1→4)-[*α*-l-rhamnopyranosyl-(1→2)]-*β*-d-glucopyranoside, named allituberoside D.

Compound **5** has a molecular formula of C_45_H_74_O_18_ as established by HR-ESI-MS. Its NMR data suggests that except for the C-3 sugar chain, **5** and **4** have the same structure. Analyses of the ^1^H-^1^H COSY spectrum give the structures of the glucose and rhamnose comprising the C-3 sugar chain, and the HMBC correlations between *δ* 5.92 (H-1′′ of Rha) and 78.3 (C-4′ of 3-*O*-Glc) confirm their connectivity. By detailed analyses of ^1^H-^1^H COSY, HSQC, and HMBC spectra, the structure of **5** is further confirmed. The C-25*S* configuration is deduced according to the chemical shifts of H-26a (*δ* 4.10) and H-26b (*δ* 3.49) (Δab > 0.57). Thus, the structure of **5** is elucidated as 26-*O*-*β*-d-glucopyranosyl-(25*S*)-5*α*-furost-Δ^20(22)^-ene-2*α*,3*β*,26-triol 3-*O*-*α*-l-rhamnopyranosyl-(1→2)-*β*-d-glucopyranoside, named allituberoside E.

Compound **6** has a molecular formula of C_51_H_84_O_22_ as identified by HR-ESI-MS. Its NMR data suggest that **6** has the identical aglycone structure with **5** and has the same C-3 sugar chain as **2**. According to its ^1^H-^1^H COSY, HSQC and HMBC spectra, the structure of **6** is confirmed. The chemical shifts of H-26a (*δ* 3.96) and H-26b (*δ* 3.63) (Δab < 0.48) further deduce its C-25*R* configuration [26]. Therefore, the structure of **6** is elucidated as 26-*O*-*β*-d-glucopyranosyl-(25*R*)-5*α*-furost-Δ^20(22)^-ene-2*α*,3*β*,26-triol 3-*O*-*α*-l-rhamnopyranosyl-(1→4)-[*α*-l-rhamnopyranosyl-(1→2)]-*β*-D-glucopyranoside, named allituberoside F.

Compound **7** with a molecular formula of C_45_H_76_O_19_ confirmed by HR-ESI-MS has the same sugar moieties as 5 by detailed comparison of the NMR data. Further comparison of the NMR data of **7** and **18** deduces that they share the same aglycone skeleton. According to its ^1^H-^1^H COSY, HSQC, and HMBC spectra, the structure of **7** is confirmed. The chemical shifts of H-26a (*δ* 4.10) and H-26b (*δ* 3.49) (Δab > 0.57) further confirm the C-25*S* configuration of **7**. Finally, the structure of **7** is elucidated as 26-*O*-*β*-d-glucopyranosyl-(25*S*)-5*β*-furost-2*β*,3*β*,26-triol 3-*O*-*α*-l-rhamnopyranosyl-(1→4)-*β*-d-glucopyranoside, named allituberoside J.

Compound **8** has the same molecular formula of C_45_H_76_O_19_ as **7** determined by HR-ESI-MS together with its ^13^C NMR data. The same ^13^C NMR data suggests that **8** and **7** are C-25*R*/*S* isomers of each other. The chemical shifts of H-26a (*δ* 3.95) and H-26b (*δ* 3.63) (Δab < 0.48) exhibit that the C-25 configuration is *R*. Consequently, the structure of **8** is elucidated as 26-*O*-*β*-d-glucopyranosyl-(25*R*)-5*β*-furost-2*β*,3*β*,26-triol 3-*O*-*α*-l-rhamnopyranosyl-(1→4)-*β*-d-glucopyranoside, named allituberoside H.

Compound **9** has a molecular formula of C_45_H_76_O_20_ as confirmed by HR-ESI-MS along with its ^13^C NMR data (Table 2). The NMR data suggests that **9** has the identical aglycone structure but has different C-3 sugar from **7**. Analysis of the ^1^H-^1^H COSY spectrum allows to establish the structures of the two glucoses comprising of the C-3 sugar chain, and the HMBC correlation between *δ* 5.39 (H-1′′) and 83.1 (C-2′) confirm their connectivity. By detailed analyses of ^1^H-^1^H COSY, HSQC, and HMBC spectra, the structure of **9** is further confirmed. The chemical shifts of H-26a (*δ* 4.10) and H-26b (*δ* 3.50) (Δab > 0.57) exhibit that the C-25*S* configuration. Consequently, the structure of **9** is elucidated as 26-*O*-*β*-d-glucopyranosyl-(25*R*)-5*β*-furost-2*β*,3*β*,26-triol 3-*O*-*β*-d-glucopyranosyl-(1→2)-*β*-d-glucopyranoside, named allituberoside I.

Compound **10** has a molecular formula of C_45_H_74_O_18_ as assigned by HR-ESI-MS. Its NMR data suggest that it shares the identical structure with **7** except for the differences surrounding the C-20 and C-22 positions. The characteristic carbon signals of *δ* 103.6 and 152.4 indicate the existence of the Δ^20(22)^-ene substructures in the molecule. Detailed analysis of ^1^H-^1^H COSY, HSQC, and HMBC spectra allows the structure of **10** to be further confirmed. The chemical shifts of H-26a (*δ* 4.09) and H-26b (*δ* 3.49) (Δab > 0.57) exhibit that the C-25*S* configuration of **10**. Thus, the structure of **10** is elucidated as 26-*O*-*β*-d-glucopyranosyl-(25*S*)-5*β*-furost-Δ^20(22)^-ene-2*α*, 3*β*, 26-diol 3-*O*-*α*-l-rhamnopyranosyl-(1→4)-*β*-d-glucopyranoside, named allituberoside J.

Compound **22** is isolated in the form of white powders with a molecular formula of C_45_H_74_O_18_ based on HR-ESI-MS and ^13^C NMR data. In its ^1^H NMR spectrum, the anomeric proton signals at *δ* 4.87 (1H, d, *J* = 7.2 Hz), 5.93 (1H, br s), and 5.47 (1H, d, *J* = 7.6 Hz) suggest that **22** has three sugar units. Comparing the NMR data of **22** and **18** reveals that they share the same C-3 sugar chain together with the substructure of A-E rings. Only three typical methyl proton signals at *δ* 0.85 (3H, s), 0.97 (3H, s), 1.18 (3H, d, *J* = 6.8 Hz) are observed in the ^1^H NMR spectrum of **22**, and its ^13^C NMR spectrum shows that the chemical shift of C-27 (*δ* 64.1) shifted to a lower field, suggesting the linkage of hydroxyl group to the C-27. By further comparing the NMR data, it is deduced that **22** had the same F-ring structure as (25*S*)-27-hydroxypenogenin-3-*O*-*α*-l-rhamnopyranosyl-(1→2)-*O*-*β*-d-glucopyranoside [15]. Thus, the structure of **22** is elucidated as (25*S*)-5*β*-spirost-3*β*, 27-diol 3-*O*-*α*-l-rhamnopyranosyl-(1→4)-[*β*-d-glucopyranosyl-(1→2)]-*β*-d-glucopyranoside, named allituberoside K.

Compound **23** has a molecular formula of C_51_H_84_O_24_ as measured by HR-ESI-MS and ^13^C NMR data. In the ^1^H NMR spectrum, the anomeric proton signals at *δ* 4.94 (2H, d, *J* = 7.8 Hz), 5.45 (1H, d, *J* = 7.6 Hz), and 5.90 (1H, br s) suggest that it has four sugar units. According to its NMR data, **23** has the same C-3 sugar chain as **22**, and shares the identical A-E rings substructure with **7**–**9**. The ^1^H NMR spectrum of **23** shows only three typical methyl proton signals at *δ* 0.78 (3H, s), 0.98 (3H, s), 1.09 (3H, d, *J* = 6.8 Hz), suggesting that its C-27 is substituted by an additional glucose unit, and that is also supported by the HMBC correlations of *δ* 4.37 (H-27a), 3.94 (H-27b)/21.4 (C-24), 33.5 (C-25), 61.0 (C-26), and of *δ* 4.94 (H-1′′′′)/69.5 (C-27). While the different NMR data of F-ring (C-22~C-27) between **23** and **27** suggest that they might have different C-25 configurations. The key carbon signals of *δ* 27.1 (C-23), 21.4 (C-24), and 61.0 (C-26) which are in accordance with those of trikamsteroside A [27] suggest that the C-25 configuration of **23** to be *R.* Thus, the structure of **23** is elucidated as 27-*O*-*β*-d-glucopyranoside-(25*R*)-5*β*-spirost-2*β*,26,27-triol 3-*O*-*α*-l-rhamnopyranosyl-(1→4)-[*β*-d-glucopyranosyl-(1→2)]-*β*-d-glucopyranoside, named allituberoside L.

Compound **24** with a molecular formula of C_45_H_74_O_19_ as deduced by HR-ESI-MS and ^13^C NMR data has the identical sugar chain at C-3 position and A-E rings substructure with **11**–**13** and shares the same F-ring including the 27-*O*-*β*-d-glucopyranose with **23** by comparing their NMR data. A detailed analysis of ^1^H-^1^H COSY, HSQC, and HMBC spectra allows the structure of **24** to be further confirmed as 27-*O*-*β*-d-glucopyranoside-(25*R*)-5*α*-spirost-2*α*,26,27-triol 3-*O*-*α*-l-rhamnopyranosyl-(1→4)-[*β*-d-glucopyranosyl-(1→2)]-*β*-d-glucopyranoside, named allituberoside M. 

Compound **25** has a molecular formula of C_51_H_84_O_23_ as determined by HR-ESI-MS and ^13^C NMR data. The NMR data suggests that, except for the different sugar chain at C-3 position, it has the identical structure with **24**. Comparison of NMR data further reveals that it has the same C-3 sugar chain as **27**. Detailed analysis of ^1^H-^1^H COSY, HSQC, and HMBC spectra finally confirms the structure of **25** to be 27-*O*-*β*-d-glucopyranoside-(25*R*)-5*α*-spirost-2*α*,26,27-triol 3-*O*-*α*-l-rhamnopyranosyl-(1→4)-[*α*-l-rhamnopyranosyl-(1→2)]-*β*-d-glucopyranoside, named allituberoside N. 

Compound **26** has a molecular formula of C_50_H_82_O_23_ as revealed according to HR-ESI-MS. Its NMR data suggests that **26** shares the identical structure with **24** except for the difference in structure of sugar chain at C-3. Further comparison of the NMR data reveals that the C-3 sugar chain of **26** is same as that of **3**. According to its ^1^H-^1^H COSY, HSQC, and HMBC spectra data, the whole structure of **26** is finally confirmed. Therefore, the structure of **26** is confirmed to be 27-*O*-*β*-d-glucopyranoside-(25*R*)-5*α*-spirost-2*α*,26,27-triol 3-*O*-*β*-d-xylopyranosyl-(1→4)-[*β*-d-glucopyranosyl-(1→2)]-*β*-d-glucopyranoside, named allituberoside O.

**Figure 2:**
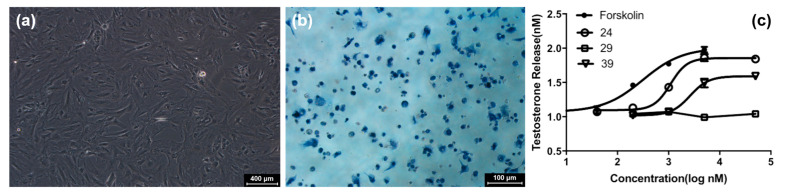
(**a**) Cell morphology of purified rat Leydig cells. (**b**) 3*β*-HSD staining of purified rat Leydig cells. The positive cells were stained in dark blue color. (**c**) Effects of compounds **15**, **21**, and **26** on testosterone secretion in Leydig cells.

An in vitro bioassay is carried out on compounds **1**–**27** for evaluating their promotional roles in testosterone production of rat Leydig cells. The results of 3*β*-HSD staining show that Leydig cells are successfully isolated from testes (Figure 2), with an approximate purity of ca. 90%. After treatment with these compounds at 50 μM, the cell survival rates are higher than 80%, suggesting that no compound exhibits the noticeable cytotoxic effect on the rat Leydig cells. 

Exposure to forskolin results in a significant increase in levels of testosterone production in Leydig cells. Similarly, **1, 2**, **7**, **8**, **10**, **13**–**15**, **20**, **23**, and **26** display a good role in increasing testosterone secretion with the EC_50_ values of 1.0 to 4.5 μM, respectively, and other compounds exhibit no significant activities (Each EC_50_ > 50 μM) (Table 3 and Figure 2).

## 3. Materials and Methods

### 3.1. Experimental Procedures for Phytochmistry Study

#### 3.1.1. General Experimental Procedures

Optical rotations were recorded on a Rudolph Autopol^®^ IV polarimeter. HR-ESI-MS was recorded on a Synapt MS (Waters Corporation, Milford, MA, USA). The NMR experiments were performed on Varian ^UNITY^ INOVA 600 spectrometer (600 MHz for ^1^H NMR and 150 MHz for ^13^C NMR). The optical rotations were measured with a JASCO J-810 polarimeter. HPLC analysis is performed on an Agilent 1100 system equipped with an Alltech 2000 evaporative light scattering detector. Semi-preparative HPLC is performed on an NP7000 module (Hanbon Co. Ltd., Huaian, China) equipped with a Shodex RID 102 detector (Showa Denko Group, Tokyo, Japan). Silgreen HPLC C_18_ columns (4.6/10.0 × 250 mm, 5 μm, Silgreen Co. Ltd., Beijing, China) were used for HPLC and Semi-preparative HPLC. Silica gel H (Qingdao Marine Chemical, Qingdao, China), AB-8 macroporous adsorption resin (Solarbio, Beijing, China), SP825 macroporous adsorption resin (Mitsubishi Chemicals, Tokyo, Japan), MCI gel (Mitsubishi Chemicals, Tokyo, Japan), and ODS silica-gel (50 μm, YMC, Kyoto, Japan) were applied for column chromatography. 

#### 3.1.2. Plant Material

The dried ATSs were purchased from Shoguang City of Shandong Province in Oct 2018, and identified by Professor Baolin Guo (Institute of Medicinal Plant Development, Chinese Academy of Medical Sciences, Beijing, China). A voucher specimen was deposited in the authors’ lab.

#### 3.1.3. Extraction and Isolation 

*Allium tuberosum* seeds (50 kg) were crushed and extracted with 70% aq. CH_3_CH_2_OH (400 L) at reflux three times (1 h for each time). The filtered solution was concentrated in vacuo to get the supernatants and sediments. The supernatants were subjected to an AB-8 macroporous adsorption resin column eluted with CH_3_CH_2_OH-H_2_O (*v/v*, 15:85→45:55→75:25→90:10) to yield five fractions (Fr.A~Fr.D). Fr.B were further subjected to a SP825 macroporous adsorption resin column eluted with CH_3_CH_2_OH-H_2_O (*v/v*, 0:100→20:80→75:25) to yield three fractions (Fr.B_1_~Fr.B_3_). Fr.B_3_ was suspended with water and partitioned with ethyl acetate and butanol in turn to yield Fr.B_3_-A and Fr.B_3_-B. Fr.B_3_-B was subjected to silica-gel column chromatography eluted with a gradient mixture of CHCl_3_-CH_3_OH (*v/v*, 5:1→4:1→3:1→0:100) to yield 66 subfractions (Fr.B_3_-B-S_1_~Fr.B_3_-B-S_66_). Fr.B_3_-B-S_36–40_ was subjected to ODS column chromatography eluted with CH_3_OH--H_2_O (*v/v*, 60:40) to afford 35 subfractions (Fr.B_3_-B-S_36–40_-O_1_~Fr.B_3_-B-S_36–40_-O_35_). Among them, Fr.B_3_-B-S_36–40_-O_16–17_ was separated by semi-preparative HPLC with CH_3_CN-H_2_O (*v/v*, 23:77, flowrate 4.0 mL/min) to afford **1** (12.5 mg); Fr.B_3_-B-S_36–40_-O_20–23_ was separated by semi-preparative HPLC with CH_3_CN-H_2_O (*v/v*, 24:76, flowrate 4.0 mL/min) to afford **7** (225.7 mg), **8** (13.5 mg), and **11** (125.6 mg); Fr.B_3_-B-S_36–40_-O_30–34_ was separated by semi-preparative HPLC with CH_3_CN-H_2_O (*v/v*, 27:73, flowrate 4.0 mL/min) to afford **10** (78.5 mg); Fr.B_3_-B-S_36–40_-O_35–36_ was separated by semi-preparative HPLC with CH_3_CN-H_2_O (*v/v*, 30:70, flowrate 4.0 mL/min) to afford **5** (18.7 mg) and **22** (4.5 mg). Fr.B_3_-B-S_48–54_ was subjected to ODS column chromatography eluted with methanol-H_2_O (*v/v*, 25:75→28:72→30:70) to afford 30 subfractions (Fr.B_3_-B-S_48–54_-O_1_~Fr.B_3_-B-S_48–54_-O_30_). Among them, Fr.B_3_-B-S_48–54_-O_6–8_ was separated by semi-preparative HPLC with CH_3_CN-H_2_O (*v/v*, 30:70, flowrate 4.0 mL/min) to afford **2** (18.3 mg), **12** (122.6 mg), **13** (20.7 mg) and **24** (20.8 mg); Fr.B_3_-B-S_48–54_-O_18–20_ was separated by semi-preparative HPLC with CH_3_CN-H_2_O (*v/v*, 26:74, flowrate 4.0 mL/min) to afford **14** (87.8 mg) and **20** (18.4 mg); Fr.B_3_-B-S_48–54_-O_26–29_ was separated by semi-preparative HPLC with CH_3_CN-H_2_O (*v*/*v*, 30:70, flowrate 4.0 mL/min) to afford **15** (48.5 mg); Fr.B_3_-B-S_48–54_-O_34–38_ was separated by semi-preparative HPLC with CH_3_CN-H_2_O (*v/v*, 30:70, flowrate 4.0 mL/min) to afford **17** (84.2 mg) and **21** (13.0 mg). Fr.B_3_-B-S_55–65_ was subjected to ODS column chromatography eluted with CH_3_OH-H_2_O (*v/v*, 23:77→30:70) to afford 39 subfractions (Fr.B_3_-B-S_55–65_-O_1_~Fr.B_3_-B-S_55–65_-O_39_). Among them, Fr.B_3_-B-S_55–65_-O_7-9_ was separated by semi-preparative HPLC with CH_3_CN-H_2_O (*v/v*, 22:78, flowrate 4.0 mL/min) to afford **9** (45.8 mg) and **18** (19.2 mg); Fr.B_3_-B-S_55–65_-O_14–15_ was separated by semi-preparative HPLC with CH_3_CN-H_2_O (*v/v*, 23:77, flowrate 4.0 mL/min) to afford **2** (53.3 mg), **3** (18.1 mg), **25** (25.6 mg), and **26** (49.6 mg); Fr.B_3_-B-S_55–65_-O_22–25_ was separated by semi-preparative HPLC with CH_3_CN-H_2_O (*v/v*, 24:76, flowrate 4.0 mL/min) to afford **18** (134.5 mg), **19** (16.5 mg), and **27** (18.3 mg); Fr.B_3_-B-S_55–65_-O_33_ was separated by semi-preparative HPLC with CH_3_CN -H_2_O (*v/v*, 25:75, flowrate 4.0 mL/min) to afford **14** (25.6 mg); Fr.B_3_-B-S_55–65_-O_34–37_ was separated by semi-preparative HPLC with CH_3_OH-H_2_O (*v/v*, 60:40, flowrate 4.0 mL/min) to afford **4** (9.4 mg), **6** (7.2 mg), and **16** (36.5 mg). Fr.B_3_-B-S_66_ was subjected to ODS column chromatography eluted with CH_3_OH-H_2_O (*v/v*, 40:60→60:40) to afford 7 subfractions (Fr.B_3_-B-S_66_-O_1_~Fr.B_3_-B-S_66_-O_7_). Among them, Fr.B_3_-B-S_66_-O_4_ was separated by semi-preparative HPLC with CH_3_CN-H_2_O (*v/v*, 22:78, flowrate 4.0 mL/min) to afford **23** (14.0 mg).

Allituberoside A (**1**): C_39_H_66_O_16_; white amorphous powder; [α]${}_{D}^{25}$−44.4 (*c* 0.036, CH_3_OH); ^1^H NMR (600 MHz, pyridine-*d*_5_) *δ* 4.71 (1H, m, H-3), 0.88 (3H, s, H-18), 1.17 (3H, s, H-19), 1.33 (3H, d, *J* = 6.8 Hz, H-21), 4.09 (1H, overlap, H-26-Ha), 3.50 (1H, dd, *J* = 8.9, 7.3 Hz, H-26-Hb), 1.05 (3H, d, *J* = 7.0 Hz, H-27), 5.11 (1H, d, *J* = 7.8 Hz, H-1′ of 3-*O*-Glc), 4.83 (1H, d, *J* = 7.8 Hz, H-1′′ of 26-*O*-Glc); ^13^C NMR (150 MHz, pyridine-*d*_5_) spectroscopic data see Table 1; HR-ESI-MS *m*/*z* 789.4272 [M − H]^−^ (calcd. for C_39_H_65_O_16_, 789.4273).

Allituberoside B (**2**): C_51_H_86_O_23_; white amorphous powder; [α]${}_{D}^{25}$ −78.5 (*c* 0.028, CH_3_OH); ^1^H NMR (600 MHz, pyridine-*d*_5_): *δ* 3.89 (1H, m, H-3), 0.87 (3H, s, H-18), 0.90 (3H, s, H-19), 1.31 (3H, d, *J* = 6.7 Hz, H-21), 4.10 (1H, overlap, H-26-Ha), 3.49 (1H, dd, *J* = 9.0, 7.2 Hz, H-26-Hb), 1.03 (3H, d, *J* = 7.0 Hz, H-27), 5.03 (1H, d, *J* = 7.1 Hz, H-1′ of 3-*O*-Glc), 6.40 (1H,br s, H-1′′ of 2′-*O*-Rha), 5.86 (1H, br s, H-1′′′ of 4′-*O*-Rha), 4.82 (1H, d, *J* = 7.8 Hz, H-1′′′′ of 26-*O*-Glc); ^13^C NMR (150 MHz, pyridine-*d*_5_) spectroscopic data see Table 1; HR-ESI-MS: *m*/*z* 1065.5466 [M − H]^−^ (calcd. for C_51_H_85_O_23_, 1065.5482).

Allituberoside C (**3**): C_50_H_84_O_23_; white amorphous powder; [α]${}_{D}^{25}$ −70.3 (*c* 0.037, CH_3_OH); ^1^H NMR (600 MHz, pyridine-*d*_5_) *δ* 3.88 (1H, m, H-3), 0.87 (3H, s, H-18), 0.91 (3H, s, H-19), 1.31 (3H, d, *J* = 6.8 Hz, H-21), 4.11 (1H, overlap, H-26-Ha), 3.49 (1H, dd, *J* = 9.2, 7.1 Hz, H-26-Hb), 1.03 (3H, d, *J* = 6.6 Hz, H-27), 5.03 (1H, d, *J* = 7.9 Hz, H-1′ of 3-*O*-Glc), 6.26 (1H, br s, H-1′′ of 2′-*O*-Rha), 5.04 (1H, d, *J* = 7.8 Hz, H-1′′′ of 4′-*O*-Xyl), 4.82 (1H, d, *J* = 8.0 Hz, H-1′′′′ of 26-*O*-Glc); ^13^C NMR (150 MHz, pyridine-*d*_5_) spectroscopic data see Table 1; HR-ESI-MS *m*/*z* 1051.5316 [M − H]^−^ (calcd. for C_50_H_83_O_23_, 1051.5325).

Allituberoside D (**4**): C_50_H_82_O_22_; white amorphous powder; [α]${}_{D}^{25}$ −75.2 (*c* 0.028, CH_3_OH); ^1^H NMR (600 MHz, pyridine-*d*_5_) *δ* 3.88 (1H, m, H-3), 0.68 (3H, s, H-18), 0.92 (3H, s, H-19), 1.62 (3H, s, H-21), 4.10 (1H, overlap, H-26-Ha), 3.49 (1H, dd, *J* = 9.1, 7.1 Hz, H-26-Hb), 1.04 (3H, d, *J* = 6.7 Hz, H-27), 5.04 (1H, d, *J* = 7.4 Hz, H-1′ of 3-*O*-Glc), 6.27 (1H, br s, H-1′′ of 2′-*O*-Rha), 5.05 (1H, d, *J* = 7.8 Hz, H-1′’’ of 4′-*O*-Xyl), 4.85 (1H, d, *J* = 7.7 Hz, H-1′′′′ of 26-*O*-Glc); ^13^C NMR (150 MHz, pyridine-*d*_5_) spectroscopic data see Table 1; HR-ESI-MS *m*/*z* 1033.5208 [M − H]^−^ (calcd. for C_50_H_81_O_22_, 1033.5219).

Allituberoside E (**5**): C_45_H_74_O_18_; white amorphous powder; [α]${}_{D}^{25}$ −66.7 (*c* 0.024, CH_3_OH); ^1^H NMR (600 MHz, pyridine-*d*_5_) *δ* 3.86 (1H, m, H-3), 0.69 (3H, s, H-18), 0.75 (3H, s, H-19), 1.62 (3H, s, H-21), 4.10 (1H, overlap, H-26-Ha), 3.49 (1H, dd, *J* = 9.1, 7.1 Hz, H-26-Hb), 1.04 (3H, d, *J* = 6.7 Hz, H-27), 5.04 (1H, d, *J* = 7.9 Hz, H-1′ of 3-*O*-Glc), 5.92 (1H, br s, H-1′′ of 2′-*O*-Rha), 4.85 (1H, d, *J* = 7.7 Hz, H-1′′′ of 26-*O*-Glc); ^13^C NMR (150 MHz, pyridine-*d*_5_) spectroscopic data see Table 1; HR-ESI-MS *m*/*z* 901.4807 [M − H]^−^ (calcd. for C_45_H_73_O_18_,901.4797).

Allituberoside F (**6**): C_51_H_84_O_22_; white amorphous powder; [α]${}_{D}^{25}$ −70.8 (*c* 0.030, CH_3_OH); ^1^H NMR (600 MHz, pyridine-*d*_5_) *δ* 3.89 (1H, m, H-3), 0.70 (3H, s, H-18), 0.91 (3H, s, H-19), 1.63 (3H, d, *J* = 6.7 Hz, H-21), 3.96 (1H, dd, *J* = 9.1, 7.9 Hz, H-26-Ha), 3.63 (1H, dd, *J* = 9.4, 5.8 Hz, H-26-Hb), 1.03 (3H, d, *J* = 7.0 Hz, H-27), 5.03 (1H, d, *J* = 6.8 Hz, H-1′ of 3-*O*-Glc), 6.41 (1H,br s, H-1′′ of 2′-*O*-Rha), 5.87 (1H, br s, H-1′′′ of 4′-*O*-Rha), 4.86 (1H, d, *J* = 8.0 Hz, H-1′′′′ of 26-*O*-Glc); ^13^C NMR (150 MHz, pyridine-*d*_5_) spectroscopic data see Table 1; HR-ESI-MS *m*/*z* 1047.5400 [M − H]^−^ (calcd. for C_51_H_83_O_22_, 1047.5376).

Allituberoside G (**7**): C_45_H_76_O_19_; white amorphous powder; [α]${}_{D}^{25}$ −69.0 (*c* 0.029, CH_3_OH); ^1^H NMR (600 MHz, pyridine-*d*_5_): *δ* 3.90 (1H, m, H-3), 0.87 (3H, s, H-18), 0.85 (3H, s, H-19), 1.33 (3H, d, *J* = 6.8 Hz, H-21), 4.10 (1H, dd, *J* = 9.4, 5.8 Hz, H-26-Ha), 3.49 (1H, dd, *J* = 9.2, 7.1 Hz, H-26-Hb), 1.04 (3H, d, *J* = 6.7 Hz, H-27), 4.94 (1H, d, *J* = 7.9 Hz, H-1′ of 3-*O*-Glc), 5.92 (1H, br s, H-1′′ of 4′-*O*-Rha), 4.83 (1H, d, *J* = 7.8 Hz, H-1′′ of 26-*O*-Glc); ^13^C NMR (150 MHz, pyridine-*d*_5_) spectroscopic data see Table 1; HR-ESI-MS *m*/*z* 919.4893 [M − H]^−^ (calcd. for C_45_H_75_O_19_, 919.4903).

Allituberoside H (**8**): C_45_H_76_O_19_; white amorphous powder; [α]${}_{D}^{25}$ −88.0 (*c* 0.025, CH_3_OH); ^1^H NMR (600 MHz, pyridine-*d*_5_) *δ* 3.90 (1H, m, H-3), 0.87 (3H, s, H-18), 0.85 (3H, s, H-19), 1.34 (3H, d, *J* = 6.4 Hz, H-21), 3.95 (1H, overlap, H-26-Ha), 3.63 (1H, dd, *J* = 9.4, 6.0 Hz, H-26-Hb), 1.04 (3H, d, *J* = 6.7 Hz, H-27), 4.94 (1H, d, *J* = 7.9 Hz, H-1′ of 3-*O*-Glc), 5.92 (1H, br s, H-1′′ of 4′-*O*-Rha), 4.84 (1H, d, *J* = 7.8 Hz, H-1′′′ of 26-*O*-Glc); ^13^C NMR (150 MHz, pyridine-*d*_5_) spectroscopic data see Table 1; HR-ESI-MS *m*/*z* 919.4892 [M − H]^−^ (calcd. for C_45_H_75_O_19_, 919.4903).

Allituberoside I (**9**): C_45_H_76_O_20_; white amorphous powder; [α]${}_{D}^{25}$ −47.4 (*c* 0.038, CH_3_OH); ^1^H NMR (600 MHz, pyridine-*d*_5_) *δ* 4.26 (1H, m, H-3), 0.87 (3H, s, H-18), 1.00 (3H, s, H-19), 1.32 (3H, d, *J* = 6.8 Hz, H-21), 4.10 (1H, overlap, H-26-Ha), 3.50 (1H, dd, *J* = 9.2, 7.1 Hz, H-26-Hb), 1.04 (3H, d, *J* = 6.6 Hz, H-27), 5.04 (1H, d, *J* = 7.8 Hz, H-1′ of 3-*O*-Glc), 5.39 (1H, d, *J* = 7.6 Hz, H-1′′ of 2′-*O*-Glc), 4.83 (1H, d, *J* = 7.8 Hz, H-1′′′ of 26-*O*-Glc); ^13^C NMR (150 MHz, pyridine-*d*_5_) spectroscopic data see Table 2; HR-ESI-MS *m*/*z* 935.4837 [M − H]^−^ (calcd. for C_45_H_75_O_20_, 935.4852).

Allituberoside J (**10**): C_45_H_74_O_18_; white amorphous powder; [α]${}_{D}^{25}$ −51.6 (*c* 0.031, CH_3_OH); ^1^H NMR (600 MHz, pyridine-*d*_5_) *δ* 4.47 (1H, m, H-3), 0.68 (3H, s, H-18), 0.87 (3H, s, H-19), 1.62 (3H, d, *J* = 6.8 Hz, H-21), 4.09 (1H, dd, *J* = 9.3, 5.8 Hz, H-26-Ha), 3.49 (1H, dd, *J* = 9.2, 7.1 Hz, H-26-Hb), 1.04 (3H, d, *J* = 6.6 Hz, H-27), 4.96 (1H, d, *J* = 7.8 Hz, H-1′ of 3-*O*-Glc), 5.93 (1H, d, *J* = 7.6 Hz, H-1′′ of 4′-*O*-Rha), 4.86 (1H, d, *J* = 7.6 Hz, H-1′′′ of 26-*O*-Glc); ^13^C NMR (150 MHz, pyridine-*d*_5_) spectroscopic data see Table 2; HR-ESI-MS *m*/*z* 901.4787 [M − H]^−^ (calcd. for C_45_H_73_O_18_, 901.4797).

Allituberoside K (**22**): C_45_H_74_O_18_; white amorphous powder; [α]${}_{D}^{25}$ −99.3 (*c* 0.025, CH_3_OH); ^1^H NMR (600 MHz, pyridine-*d*_5_) *δ* 4.26 (1H, m, H-3), 0.85 (3H, s, H-18), 0.97 (3H, s, H-19), 1.18 (3H, d, *J* = 6.8 Hz, H-21), 4.17 (1H, dd, *J* = 10.6, 3.8 Hz, H-27-Ha), 3.93 (1H, t, *J* = 11.1 Hz, H-27-Hb), 4.87 (1H, d, *J* = 7.2 Hz, H-1′ of 3-*O*-Glc), 5.47 (1H, d, *J* = 7.6 Hz, H-1′′ of 2′-*O*-Glc), 5.93 (1H, br s, H-1′′′ of 4′-*O*-Rha); ^13^C NMR (150 MHz, pyridine-*d*_5_) spectroscopic data see Table 2; HR-ESI-MS *m*/*z* 901.4800 [M − H]^−^ (calcd. for C_45_H_73_O_18_,901.4797).

Allituberoside L (**23**): C_51_H_84_O_24_; white amorphous powder; [α]${}_{D}^{25}$ −108.3 (*c* = 0.024, CH_3_OH); ^1^H NMR (600 MHz, pyridine-*d*_5_) *δ* 4.27 (1H, m, H-3), 0.78 (3H, s, H-18), 0.98 (3H, s, H-19), 1.09 (3H, d, *J* = 6.8 Hz, H-21), 4.37 (1H, overlap, H-27-Ha), 3.94 (1H, t, *J* = 8.5 Hz, H-27-Hb), 4.94 (1H, d, *J* = 7.8 Hz, H-1′ of 3-*O*-Glc), 5.45 (1H, d, *J* = 7.6 Hz, H-1′′ of 2′-*O*-Glc), 5.90 (1H, br s, H-1′′′ of 4′-*O*-Rha), 4.94 (1H, d, *J* = 7.8 Hz, H-1′′′′ of 27-*O*-Glc); ^13^C NMR (150 MHz, pyridine-*d*_5_) spectroscopic data see Table 2; HR-ESI-MS *m*/*z* 1079.5267 [M − H]^−^ (calcd. for C_51_H_83_O_24_, 1079.5274).

Allituberoside M (**24**): C_45_H_74_O_19_; white amorphous powder; [α]${}_{D}^{25}$ −87.2 (*c* 0.039, CH_3_OH); ^1^H NMR (600 MHz, pyridine-*d*_5_) *δ* 3.93 (1H, m, H-3), 0.77 (3H, s, H-18), 0.89 (3H, s, H-19), 1.08 (3H, d, *J* = 6.9 Hz, H-21), 4.36 (1H, overlap, H-27-Ha), 3.92 (1H, overlap, H-27-Hb), 5.10 (1H, d, *J* = 7.6 Hz, H-1′ of 3-*O*-Glc), 6.39 (1H, br s, H-1′′ of 2′-*O*-Glc), 4.93 (1H, d, *J* = 7.8 Hz, H-1′′′ of 27-*O*-Glc); ^13^C NMR (150 MHz, pyridine-*d*_5_) spectroscopic data see Table 2; HR-ESI-MS *m*/*z* 917.4756 [M − H]^−^ (calcd. for C_45_H_73_O_19_,917.4746).

Allituberoside N (**25**): C_51_H_84_O_23_; white amorphous powder; [α]${}_{D}^{25}$ −93.8 (*c* 0.032, CH_3_OH); ^1^H NMR (600 MHz, pyridine-*d*_5_) *δ* 3.88 (1H, m, H-3), 0.77 (3H, s, H-18), 0.89 (3H, s, H-19), 1.07 (3H, d, *J* = 6.3 Hz, H-21), 4.37 (1H, overlap, H-27-Ha), 3.92 (1H, t, *J* = 8.5 Hz, H-27-Hb), 5.03 (1H, d, *J* = 7.3 Hz, H-1′ of 3-*O*-Glc), 6.40 (1H, br s, H-1′′ of 2′-*O*-Glc), 5.86 (1H, br s, H-1′′′ of 4′-*O*-Rha), 4.93 (1H, d, *J* = 7.8 Hz, H-1′′′′ of 27-*O*-Glc); ^13^C NMR (150 MHz, pyridine-*d*_5_) spectroscopic data see Table 2; HR-ESI-MS *m*/*z* 1063.5320 [M − H]^−^ (calcd. for C_51_H_83_O_23_, 1063.5325).

Allituberoside O (**26**): C_50_H_82_O_23_; white amorphous powder; [α]${}_{D}^{25}$ −84.6 (*c* 0.026, CH_3_OH); ^1^H NMR (600 MHz, pyridine-*d*_5_) *δ* 3.88 (1H, m, H-3), 0.77 (3H, s, H-18), 0.89 (3H, s, H-19), 1.07 (3H, d, *J* = 6.3 Hz, H-21), 4.36 (1H, overlap, H-27-Ha), 3.93 (1H, overlap, H-27-Hb), 5.03 (1H, d, *J* = 7.6 Hz, H-1′ of 3-*O*-Glc), 6.26 (1H, br s, H-1′′ of 2′-*O*-Glc), 5.04 (1H, d, *J* = 7.6 Hz, H-1′′′ of 4′-*O*-Rha), 4.93 (1H, d, *J* = 7.7 Hz, H-1′′′′ of 27-*O*-Glc); ^13^C NMR (150 MHz, pyridine-*d*_5_) spectroscopic data see Table 2; HR-ESI-MS *m*/*z* 1049.5180 [M − H]^−^ (calcd. for C_50_H_81_O_23_, 1049.5169).

#### 3.1.4. Acid Hydrolysis and Absolute Configuration Determination

Compounds **1**–**10** and **20**–**26** (each 1.0 mg) were individually hydrolyzed by heating in 1 mL of 6 M TFA at 90 °C for 2 h. After cooling, the reaction mixture was extracted with CHCl_3_. Then, each aqueous layer was evaporated to dryness, and the residue was dissolved in 1 mL of pyridine containing 1 mg of L-cysteine methyl ester hydrochloride and further heated at 60 °C for 1 h. Following, o-tolyl isothiocyanate (5 μL) was added to each mixture, and heated at 60 °C for 1 h. Standard sugars (each 5 mg) and L-cysteine methyl ester hydrochloride (5 mg) was dissolved in pyridine (5 mL) and heated to 60 °C for 1 h. Then o-tolyl isothiocyanate (10 μL) was added to the mixture and refluxed for 1 h. The reaction mixture was analyzed by HPLC. As a result, the D-configurations for glucoses and xylose, L-configrations for rhamnoses in the corresponding compounds were identified by comparing the retention time with the standards. 

### 3.2. Bioactivity Assay

#### 3.2.1. Preparation of Rat Leydig Cells and Primary Culture

Leydig cells were isolated from 50–70-day-old Sprague Dawley rats followed the procedure described in the literature with some modifications [28]. In a nutshell, the decapsulated testes were minced into 2–3 mm pieces on the icebox and dispersed in the DME/F-12 medium (Hyclone) for 15 min at 34 °C with gentle shaking. The suspension was repeatedly dissociated with a Pasteur pipette to break up large clumps, then dissolved in 0.05% collagenase I (Invitrogen) dissociation medium. Subsequently, the digestion was stopped by DMEM-F12 culture medium containing 9% bovine serum albumin, 1% horse serum, and 0.5% penicillin-streptomycin mixture (GIBCO), and the solution was filtered through a nylon mesh (70 µm). The gradient was centrifuged for 30 min at 800× *g* at 4 °C, and cells localized between Percoll gradient 70 and 58% were isolated (the second layer). After the repeating wash steps of the medium, the Leydig cells were incubated in the DMEM-F12 culture medium.

The purity of Leydig cells were determined by 3*β*-hydroxysteroid dehydrogenase (3*β*-HSD) histochemical staining [29]. Leydig cells were maintained in 24-well plates at 37 °C with 5% CO_2_. The staining solution contained PBS supplemented with 0.1 mg/mL nitro-blue tetrazolium (Biosharp), 1.0 mg/mL nicotinamide adenine dinucleotide (Sigma-Aldrich), 0.1 mg/mL dehydroepiandrosterone (Sigma-Aldrich, Burlington, MA, USA), and 0.1 mg/mL niacinamide for 90 min. The positive cells were stained a dark blue.

Animal experiments were approved by the Institutional Animal Care and Use Committee and the local experimental Ethics Committee (Laboratory Animal Certificate no. SYXK2017-0067). Male Sprague-Dawley rats were purchased from the Hubei Provincial Center for Disease Control and Prevention (SCXK 2015-0018; Wuhan, China).

#### 3.2.2. Cellular Viability and Testosterone Production

Purified Leydig cells (5 × 10^3^/mL) were cultured in 96-well plates at 37 °C with 5% CO_2_ for 48 h. The cells were afterward cultured in serum-free medium containing different doses of compounds, forskolin, HCG (1 IU/mL) for 24 h. Cellular viability was evaluated using the MTT proliferation assay. The MTT (Sigma-Aldrich) solution was maintained for 4 h, then 100 μL DMSO was added. Finally, the absorbance was measured at 570 nm by a microplate reader (Synergy HT). Testosterone secreted into the culture medium was measured using ELISA kits according to the manufacturer’s instructions (Nanjing Jiancheng Biological Technology, Nanjing, China).

## 4. Conclusions

This phytochemical work presented a study on ATSs leading to the isolation of 27 steroidal saponins, which facilitates understanding the structural composition of steroidal saponins as the main constituents in ATSs. The subsequent activity assay shows that nearly half of the isolated steroidal glycosides can remarkably promote the testosterone production of rat Leydig cells, proving that the steroidal saponin could be considered as the basis of active material of this traditional herb medicine for playing a role in treating both impotence and nocturnal emissions. The result of this work reveals the active substance basis of ASTs to some extent. Meanwhile, this work clarifies the structure of steroidal saponin in ASTs, establishing a foundation for the quality control research of this traditional medicine.

## Figures and Tables

**Table 1:** ^13^C NMR data for **1**–**8** (*δ* in pyridine-*d*_5_).

*POS.*	1	2	3	4	5	6	7	8
1	36.0	45.9	45.9	46.0	45.8	45.9	40.3	40.3
2	29.2	70.6	70.6	70.5	70.4	70.6	66.9	66.9
3	79.2	85.1	85.1	85.1	84.9	85.1	80.2	80.2
4	35.2	33.5	33.5	33.5	33.9	33.5	31.7	31.7
5	73.1	44.6	44.7	44.7	44.6	44.7	36.4	36.4
6	66.2	28.2	28.2	28.2	28.1	28.2	26.2	26.2
7	35.6	32.3	32.3	32.5	32.4	32.4	26.7	26.7
8	34.6	34.6	34.6	34.4	34.4	34.4	35.5	35.5
9	44.7	54.5	54.5	54.4	54.4	54.4	41.4	41.4
10	43.1	36.9	36.9	36.9	36.9	36.9	36.9	36.9
11	21.9	21.5	21.5	21.6	21.6	21.6	21.3	21.4
12	40.1	40.1	40.2	39.8	39.8	39.8	40.5	40.5
13	41.0	41.1	41.1	43.7	43.7	43.8	41.2	41.2
14	56.3	56.3	56.3	54.7	54.6	54.6	56.3	56.3
15	32.4	32.4	32.4	34.4	34.3	34.4	32.4	32.4
16	81.1	81.1	81.2	84.5	84.5	84.5	81.2	81.2
17	63.8	63.9	63.9	64.6	64.6	64.6	64.0	64.0
18	16.7	16.7	16.7	14.4	14.4	14.4	16.7	16.7
19	17.6	13.6	13.6	13.6	13.4	13.6	23.8	23.8
20	40.7	40.7	40.7	103.6	103.6	103.6	40.7	40.7
21	16.5	16.5	16.5	11.8	11.8	11.8	16.5	16.5
22	110.7	110.6	110.6	152.4	152.4	152.4	110.6	110.6
23	37.2	37.2	37.2	31.4	31.4	31.5	37.2	37.3
24	28.4	28.3	28.4	23.7	23.6	23.7	28.4	28.4
25	34.5	34.5	34.5	33.8	33.7	33.4	34.5	34.3
26	75.4	75.4	75.4	75.3	75.2	75.0	75.4	75.3
27	17.5	17.5	17.5	17.2	17.2	17.4	17.5	17.5
	3-*O*-Glc	3-*O*-Glc	3-*O*-Glc	3-*O*-Glc	3-*O*-Glc	3-*O*-Glc	3-*O*-Glc	3-*O*-Glc
1′	102.2	100.9	100.6	100.6	103.0	100.9	104.0	104.0
2′	74.8	77.9	77.3	77.3	75.3	77.9	75.0	75.0
3′	79.0	77.9	76.5	76.5	76.6	77.9	76.6	76.6
4′	71.6	78.5	81.4	81.4	78.3	78.7	78.1	78.1
5′	78.7	77.2	77.8	77.8	77.4	77.2	77.4	77.5
6′	62.4	61.1	61.4	61.4	61.3	61.1	61.3	61.3
	26-*O*-Glc	2′-*O*-Rha	2′-*O*-Rha	2′-*O*-Rha	4′-*O*-Rha	2′-*O*-Rha	4′-*O*-Rha	4′-*O*-Rha
1″	105.2	102.1	102.2	102.1	102.7	102.1	102.7	102.7
2″	75.3	72.5	72.4	72.4	72.6	72.5	72.6	72.7
3″	78.6	72.8	72.8	72.8	72.8	72.8	72.8	72.8
4″	71.7	74.1	74.1	74.1	74.0	74.1	74.0	74.0
5″	78.5	69.5	69.6	69.6	70.5	69.5	70.4	70.4
6″	62.8	18.6	18.6	18.6	18.6	18.6	18.6	18.6
		4′-*O*-Rha	4′-*O*-Xyl	4′-*O*-Xyl	26-*O*-Glc	4′-*O*-Rha	26-*O*-Glc	26-*O*-Glc
1‴		102.9	105.8	105.8	105.2	103.0	105.2	105.0
2‴		72.6	75.0	75.0	75.2	72.6	75.2	75.2
3‴		72.8	78.4	78.4	78.6	72.8	78.6	78.6
4‴		73.9	70.8	70.8	71.7	73.9	71.7	71.7
5‴		70.5	67.4	67.4	78.6	70.5	78.5	78.5
6‴		18.5			62.8	18.6	62.8	62.8
		26-*O*-Glc	26-*O*-Glc	26-*O*-Glc		26-*O*-Glc		
1‴′		105.2	105.2	105.2		104.9		
2‴′		75.3	75.3	75.3		75.2		
3‴′		78.6	78.7	78.7		78.7		
4‴′		71.7	71.7	71.7		71.7		
5‴′		78.6	78.5	78.6		78.6		
6‴′		62.8	62.8	62.9		62.9		

**Table 2:** ^13^C NMR data for **9**, **10** and **22**–**26** (*δ* in pyridine-*d*_5_).

*No.*	9	10	22	23	24	25	26
1	40.3	40.0	30.9	40.4	45.9	45.9	45.9
2	67.2	67.0	27.0	67.1	70.7	70.6	70.6
3	81.7	80.1	75.3	81.1	85.4	85.0	85.1
4	31.6	31.7	30.7	31.2	33.6	33.5	33.5
5	36.5	36.4	36.7	32.1	44.6	44.6	44.6
6	26.3	26.2	26.8	26.3	28.2	28.2	28.2
7	26.8	26.8	26.8	26.8	32.1	32.1	32.1
8	35.6	35.2	35.5	35.5	34.6	34.6	34.6
9	41.5	36.9	40.3	41.4	54.4	54.4	54.4
10	37.1	36.9	35.3	37.0	36.9	36.9	36.9
11	21.4	40.5	21.2	21.3	21.4	21.5	21.5
12	40.6	40.5	40.2	40.2	40.0	40.0	40.0
13	41.2	43.8	40.9	40.8	40.7	40.8	40.8
14	56.3	54.6	56.5	56.3	56.3	56.3	56.3
15	32.4	34.4	32.2	32.1	32.3	32.3	32.3
16	81.2	84.5	81.3	81.4	81.2	81.3	81.3
17	64.0	64.6	62.9	63.0	62.9	62.9	62.9
18	16.7	14.4	16.6	16.5	16.5	16.6	16.6
19	23.8	23.8	24.0	23.9	13.5	13.6	13.6
20	40.7	103.6	42.1	42.4	42.4	42.4	42.4
21	16.5	11.8	15.1	14.9	14.8	14.8	14.8
22	110.6	152.4	109.7	109.7	109.7	109.7	109.7
23	37.3	31.4	31.6	27.1	27.1	27.1	27.1
24	28.4	23.6	24.1	21.4	21.4	21.5	21.5
25	34.5	33.7	39.2	33.5	33.5	33.5	33.5
26	75.4	75.2	64.4	61.0	60.9	60.9	60.9
27	17.5	17.2	64.1	69.5	69.5	69.5	69.5
	3-*O*-Glc	3-*O*-Glc	3-*O*-Glc	3-*O*-Glc	3-*O*-Glc	3-*O*-Glc	3-*O*-Glc
1′	102.7	104.0	101.9	102.3	101.2	100.8	100.5
2′	83.1	75.4	82.8	82.3	78.1	77.9	77.8
3′	78.1	76.6	77.1	77.1	79.6	78.0	76.5
4′	71.4	78.1	77.3	77.3	71.9	78.5	81.4
5′	78.4	77.5	76.4	76.3	78.4	77.2	77.3
6′	62.4	61.3	61.3	61.0	62.5	61.1	61.5
	2′-*O*-Glc	4′-*O*-Rha	2′-*O*-Glc	2′-*O*-Glc	2′-*O*-Rha	2′-*O*-Rha	2′-*O*-Rha
1″	106.2	102.7	105.7	105.6	102.2	102.2	102.2
2″	77.1	72.7	77.1	77.0	72.5	72.5	72.4
3″	77.9	72.8	77.9	78.0	72.8	72.8	72.8
4″	71.8	74.0	71.8	71.9	74.2	74.1	74.1
5″	78.5	70.4	78.6	78.5	69.5	69.6	69.6
6″	62.8	18.6	63.2	62.9	18.6	18.6	18.6
	26-*O*-Glc	26-*O*-Glc	4′-*O*-Rha	4′-*O*-Rha	27-*O*-Glc	4′-*O*-Rha	4′-*O*-Xyl
1‴	105.2	105.2	102.4	102.4	105.0	103.0	105.8
2‴	75.3	75.0	72.6	72.5	75.3	72.6	75.4
3‴	78.7	78.6	72.8	72.8	78.6	72.8	78.4
4‴	71.7	71.7	74.0	74.0	71.8	74.0	70.8
5‴	78.6	78.6	70.3	70.3	78.6	70.5	67.4
6‴	62.8	62.8	18.5	18.6	62.8	18.5	
				27-*O*-Glc		27-*O*-Glc	27-*O*-Glc
1‴′				105.0		105.1	105.1
2‴′				75.3		75.4	75.0
3‴′				78.6		78.7	78.6
4‴′				71.7		71.8	71.8
5‴′				78.6		78.7	78.5
6‴′				62.9		62.8	62.8

**Table 3:** Roles of compounds 1-27 in promoting testosterone production of rat Leydig cells.

Compound	EC_50_ (μM)	Compounds	EC_50_ (μM)	Compound	EC_50_ (μM)	Compounds	EC_50_ (μM)
**1**	1.0	**8**	1.6	**15**	1.1	**22**	>50
**2**	2.6	**9**	>50	**16**	>50	**23**	2.1
**3**	>50	**10**	1.4	**17**	>50	**24**	>50
**4**	>50	**11**	>50	**18**	>50	**25**	>50
**5**	>50	**12**	1.8	**19**	>50	**26**	4.5
**6**	>50	**13**	1.0	**20**	2.0	**27**	>50
**7**	4.4	**14**	1.6	**21**	>50	Forskolin	0.3

## Supplementary Materials

NMR spectra of compounds **1**–**27** are available.
