# Supplementary material for: Steroidal Glycosides from Allium tuberosum Seeds and Their Roles in Promoting Testosterone Production of Rat Leydig Cells
Source: Molecules. 2020 Nov 22;25(22):5464. doi: 10.3390/molecules25225464 (PMC7700350; doi:10.3390/molecules25225464)
Supplement: Supplementary file 1 [file molecules-25-05464-s001.pdf]

Article

Steroidal glycosides from *Allium Tuberosum* seeds and their roles in promoting testosterone production of rat leydig cells

Da-Bing Zhang[1,2] and Xian-Yong Wei [1,3,]

[1.] Key Laboratory of Coal Processing and Efficient Utilization, Ministry of Education, China University of Mining & Technology, Xuzhou, Jiangsu 221116, China

[2.] Jiangsu Hanbon Science & Technology Co., Ltd, Huaian, Jiangsu 223005, China

[3.] State Key Laboratory of High-efficiency Coal Utilization and Green Chemical Engineering,Ningxia University, Yinchuan 750021, Ningxia, China

∗ Correspondence: wei_xianyong@163.com (X.-Y. Wei).

Abstract: A systematic phytochemical study on the components in the seeds of *Allium tuberosum* was performed, leading to the isolation of 27 steroidal glycosides (1–27). The structures of all compounds were established mainly by NMR and MS experiments as well as the necessary chemical evidence, of which 1–10 and 22–26 (Allituberosides A–O) are new steroidal saponin analogues. An in vitro bioassay indicated that 1, 2, 7, 8, 10, 13–15, 20, 23, and 26 displayed the promotional roles in testosterone production of rat Leydig cells with the EC50 values of 1.0 to 4.5 μM, respectively.

Table 1. ^{13}C NMR data for compounds **11–16** and **27** (150 MHz, δ in pyridine-d_5).

POS.	11	12	13	14	15	16
1	45.7	45.9	45.9	37.2	45.9	45.9
2	70.4	70.7	70.7	29.9	70.7	70.5
3	85.0	85.5	85.5	77.2	85.4	85.0
4	33.9	33.7	33.7	34.5	33.7	33.4
5	44.7	44.7	44.7	44.6	44.7	44.6
6	28.1	28.2	28.2	29.0	28.2	28.2
7	32.3	32.3	32.3	32.4	32.4	32.4
8	34.6	34.6	34.6	35.3	34.4	34.4
9	54.4	54.4	54.4	54.5	54.4	54.4
10	36.9	36.9	36.9	35.9	36.9	36.9
11	21.5	21.5	21.5	21.3	21.6	21.6
12	40.1	40.1	40.1	40.2	39.8	39.8
13	41.1	41.1	41.1	41.1	43.7	43.7
14	56.3	56.3	56.3	56.4	54.7	54.6
15	32.4	32.4	32.4	32.5	34.4	34.3
16	81.1	81.1	81.1	81.1	84.5	84.5
17	63.9	63.9	63.9	64.0	64.6	64.6
18	16.7	16.7	16.7	16.7	14.4	14.4
19	13.4	13.5	13.5	12.4	13.5	13.5
20	40.7	40.7	40.7	40.7	103.6	103.6
21	16.5	16.5	16.5	16.5	11.8	11.8
22	110.6	110.6	110.6	110.6	152.4	152.4

23	37.2	37.2	37.2	37.2	31.4	31.4
24	28.4	28.3	28.3	28.4	23.7	23.7
25	34.4	34.4	34.4	34.5	33.6	33.8
26	75.2	75.2	75.2	75.4	75.2	75.0
27	17.5	17.5	17.5	17.5	17.2	17.2
	3-*O*-Glc	3-*O*-Glc	3-*O*-Glc	3-*O*-Glc	3-*O*-Glc	3-*O*-Glc
1	103.0	101.3	101.3	99.9	101.3	100.8
2'	75.4	78.0	78.0	78.0	78.0	77.9
3'	76.6	79.6	79.6	78.7	79.6	78.7
4'	78.3	71.9	71.9	78.1	71.9	77.9
5'	77.4	78.4	78.4	77.0	78.5	77.0
6'	61.3	62.5	62.5	61.3	62.5	61.1
	4'-*O*-Rha	2'-*O*-Rha	2'-*O*-Rha	2'-*O*-Rha	2'-*O*-Rha	2'-*O*-Rha
1''	102.7	102.2	102.2	102.2	102.2	102.1
2''	72.6	72.5	72.5	72.5	72.5	72.4
3''	72.8	72.8	72.8	72.8	72.8	72.7
4''	74.0	74.2	74.2	74.1	74.0	74.1
5''	70.5	69.5	69.5	69.5	69.5	69.5
6''	18.6	18.6	18.6	18.7	18.6	18.6
	26-*O*-Glc	26-*O*-Glc	26-*O*-Glc	4'-*O*-Rha	26-*O*-Glc	4'-*O*-Rha
1'''	105.2	105.2	105.2	102.9	105.2	102.9
2'''	75.2	75.4	75.4	72.6	75.4	72.5
3'''	78.6	78.6	78.6	72.8	78.6	72.8
4'''	71.7	71.7	71.7	74.0	71.7	73.9
5'''	78.5	78.5	78.5	70.4	78.6	70.5
6'''	62.8	62.8	62.8	18.5	62.8	18.5
				26-*O*-Glc		26-*O*-Glc
1''''				105.2		105.2
2''''				75.3		75.3
3''''				78.6		78.6
4''''				71.7		71.7
5''''				78.5		78.5
6''''				62.8		62.9

Table 2. ^{13}C NMR data for compounds **17–21** and **27** (150 MHz, δ in pyridine-d_5).

POS.	**17**	**18**	**19**	**20**	**21**	**27**
1	37.6	30.9	30.9	37.5	37.6	45.9
2	30.2	27.0	27.0	30.2	30.2	70.6
3	77.1	75.4	75.3	77.8	77.7	85.0
4	34.5	30.7	30.7	39.0	39.0	33.5
5	44.6	36.8	36.8	140.8	140.8	44.6
6	29.0	26.8	26.8	121.9	121.8	28.2
7	32.6	26.8	26.8	32.4	32.4	32.1
8	35.0	35.5	35.5	31.7	31.5	34.6
9	54.4	40.4	40.4	50.4	50.3	54.4

10	35.9	35.3	35.3	37.2	37.1	36.9
11	21.5	21.2	21.2	21.1	21.3	21.5
12	39.9	40.3	40.3	39.9	39.7	40.1
13	43.7	40.7	40.7	40.8	43.4	40.8
14	54.8	56.5	56.5	56.6	55.0	56.3
15	34.4	32.5	32.5	32.5	34.5	32.3
16	84.5	81.3	81.3	81.1	84.5	81.2
17	64.6	64.1	64.1	63.8	64.5	63.7
18	14.4	16.8	16.8	16.5	14.1	16.6
19	12.4	24.0	24.0	19.4	19.4	13.6
20	103.6	41.3	41.3	40.7	103.6	42.0
21	11.8	16.5	16.5	16.5	11.8	15.0
22	152.4	110.7	110.7	110.7	152.4	109.5
23	31.4	37.2	37.2	37.2	31.4	31.3
24	23.7	28.4	28.4	28.3	23.7	24.0
25	33.7	34.5	34.5	34.5	33.5	36.7
26	75.2	75.3	75.3	75.4	75.2	62.9
27	17.2	17.5	17.5	17.5	17.4	72.0
	3-*O*-Glc	3-*O*-Glc	3-*O*-Glc	3-*O*-Glc	3-*O*-Glc	3-*O*-Glc
1	99.8	101.9	101.9	100.3	100.3	100.8
2'	78.0	82.9	82.9	77.8	78.0	78.0
3'	78.7	77.1	77.1	78.6	78.7	78.6
4'	78.0	77.3	77.3	78.0	78.0	77.9
5'	77.0	76.5	76.5	77.0	77.0	77.2
6'	61.3	61.3	61.3	61.3	61.3	61.1
	2'-*O*-Rha	2'-*O*-Rha	2'-*O*-Rha	2'-*O*-Rha	2'-*O*-Rha	2'-*O*-Rha
1''	102.2	105.7	105.7	102.0	102.0	102.2
2''	72.5	77.1	77.1	72.7	72.6	72.5
3''	72.8	78.0	78.0	72.8	72.8	72.8
4''	74.1	71.9	71.9	74.0	74.2	74.1
5''	69.5	78.6	78.6	70.4	69.5	69.5
6''	18.7	63.0	63.0	18.7	18.7	18.6
	4'-*O*-Rha	4'-*O*-Rha	4'-*O*-Rha	4'-*O*-Rha	4'-*O*-Rha	4'-*O*-Rha
1'''	102.9	102.5	102.5	102.9	102.9	103.0
2'''	72.6	72.6	72.6	72.6	72.6	72.6
3'''	72.8	72.8	72.8	72.9	72.9	72.8
4'''	74.0	74.1	74.1	74.2	74.0	74.0
5'''	70.4	70.3	70.3	70.4	70.4	70.5
6'''	18.5	18.6	18.6	18.5	18.5	18.6
	26-*O*-Glc	26-*O*-Glc	26-*O*-Glc	26-*O*-Glc	26-*O*-Glc	27-*O*-Glc
1''''	104.9	105.2	105.0	105.2	104.9	105.1
2''''	75.2	75.4	75.4	75.3	75.2	75.2
3''''	78.6	78.7	78.7	78.5	78.6	78.6
4''''	71.7	71.7	71.7	71.7	71.7	71.7
5''''	78.6	78.6	78.6	78.5	78.6	78.6
6''''	62.9	62.8	62.8	62.8	62.9	62.9

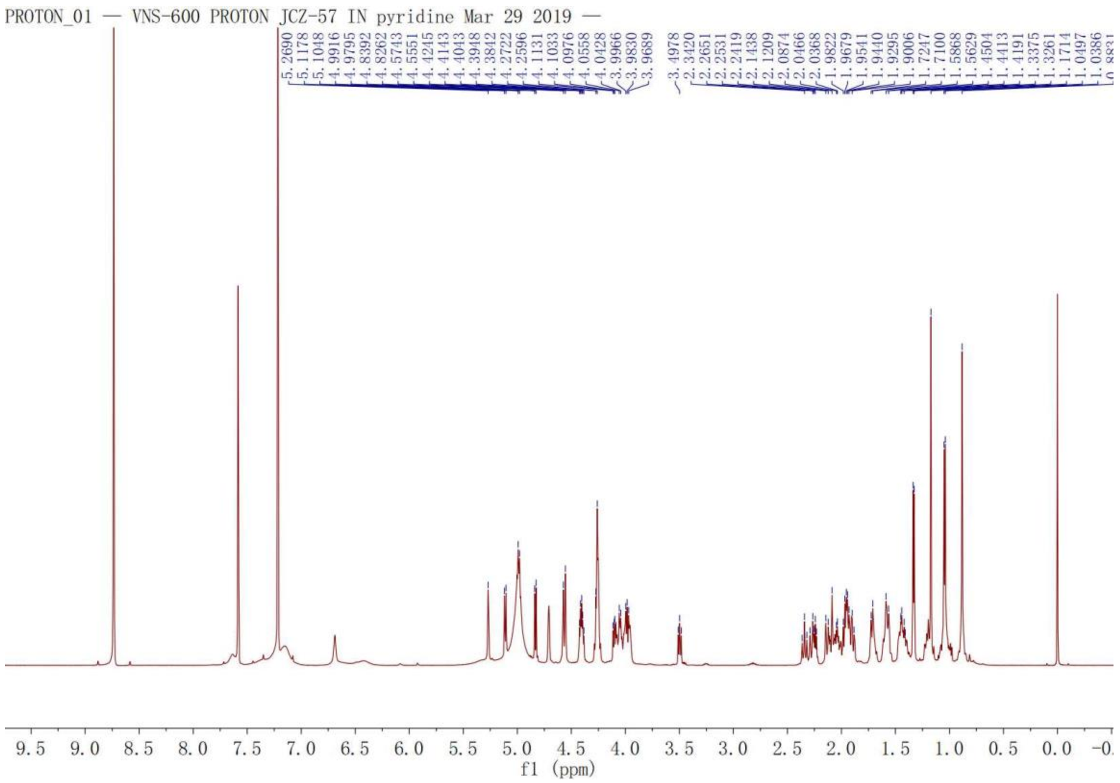

^{1}H NMR spectrum of 1.

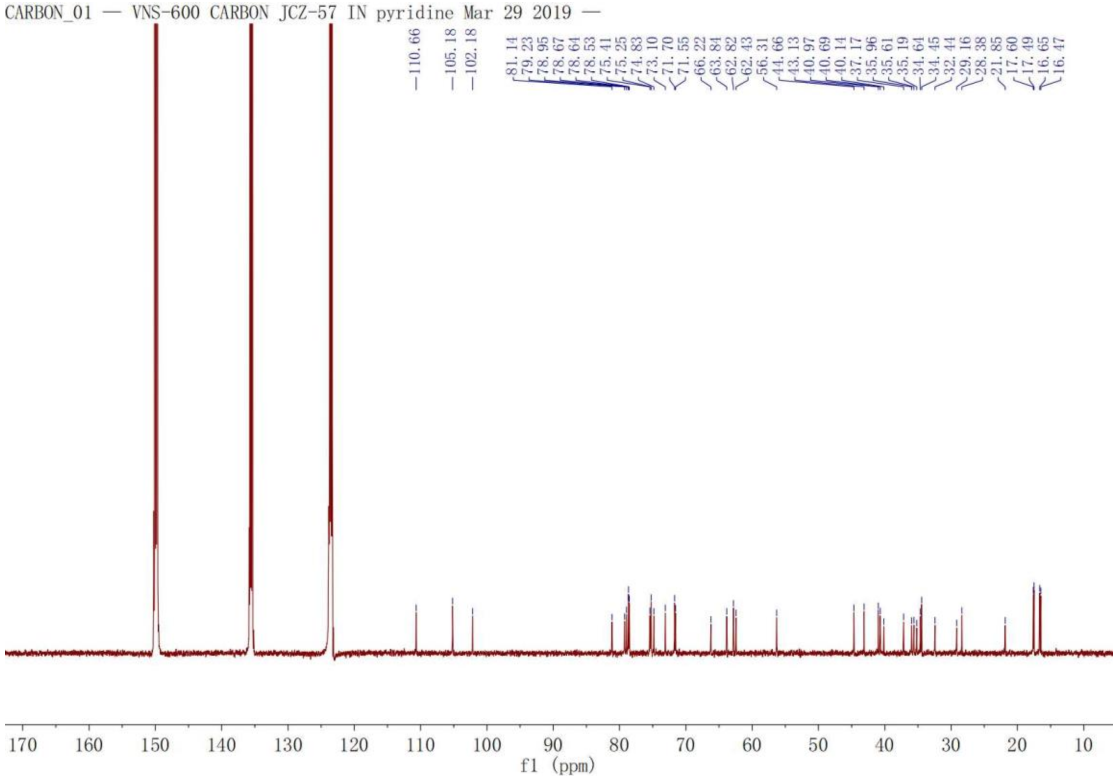

^{13}C NMR spectrum of 1.

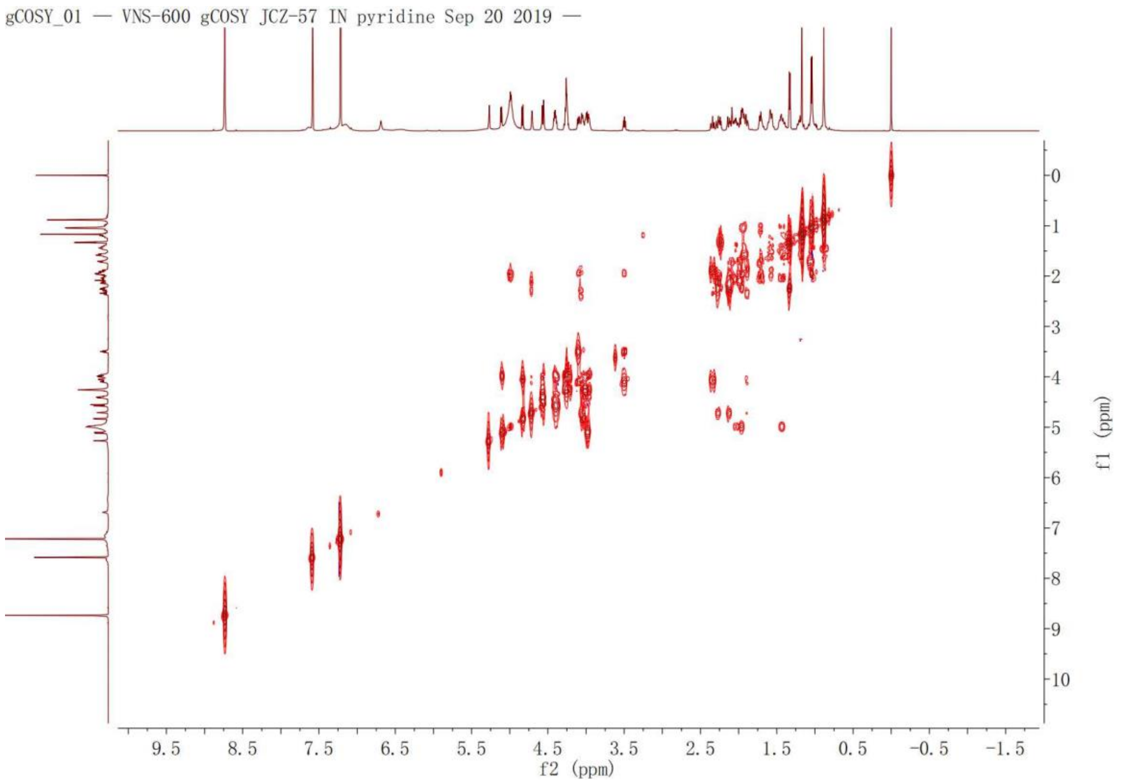

^1H-^1H COSY spectrum of 1.

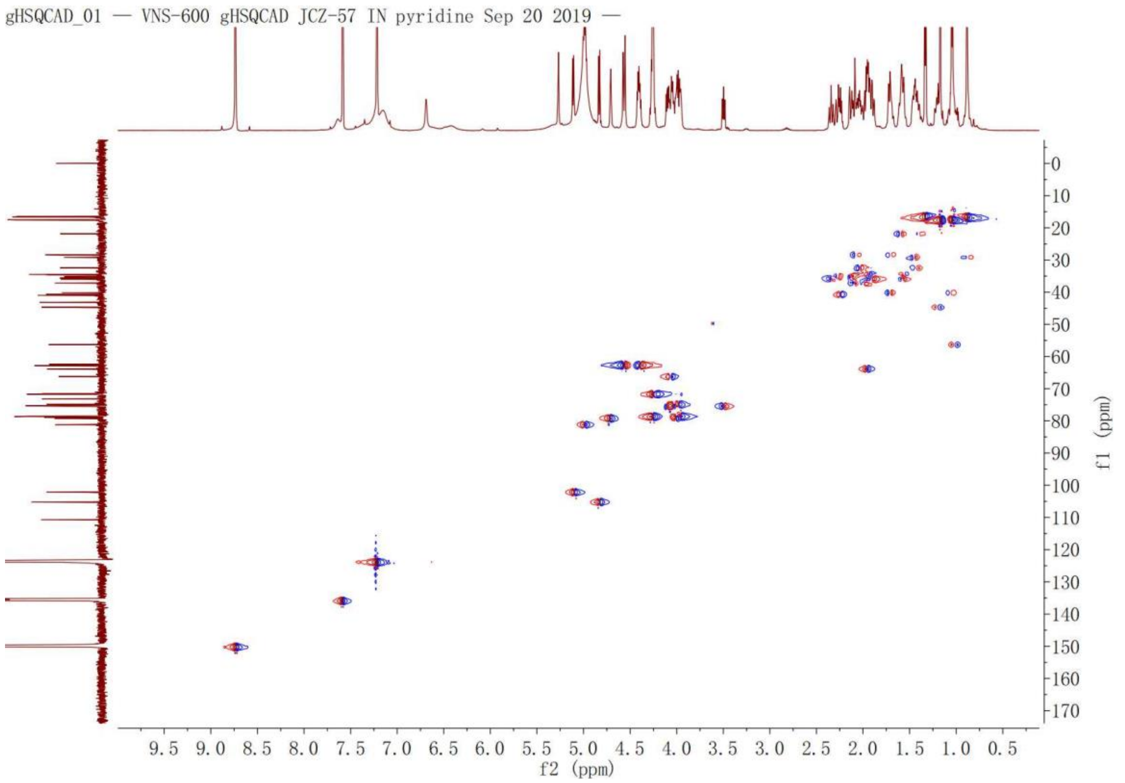

HSQC spectrum of 1.

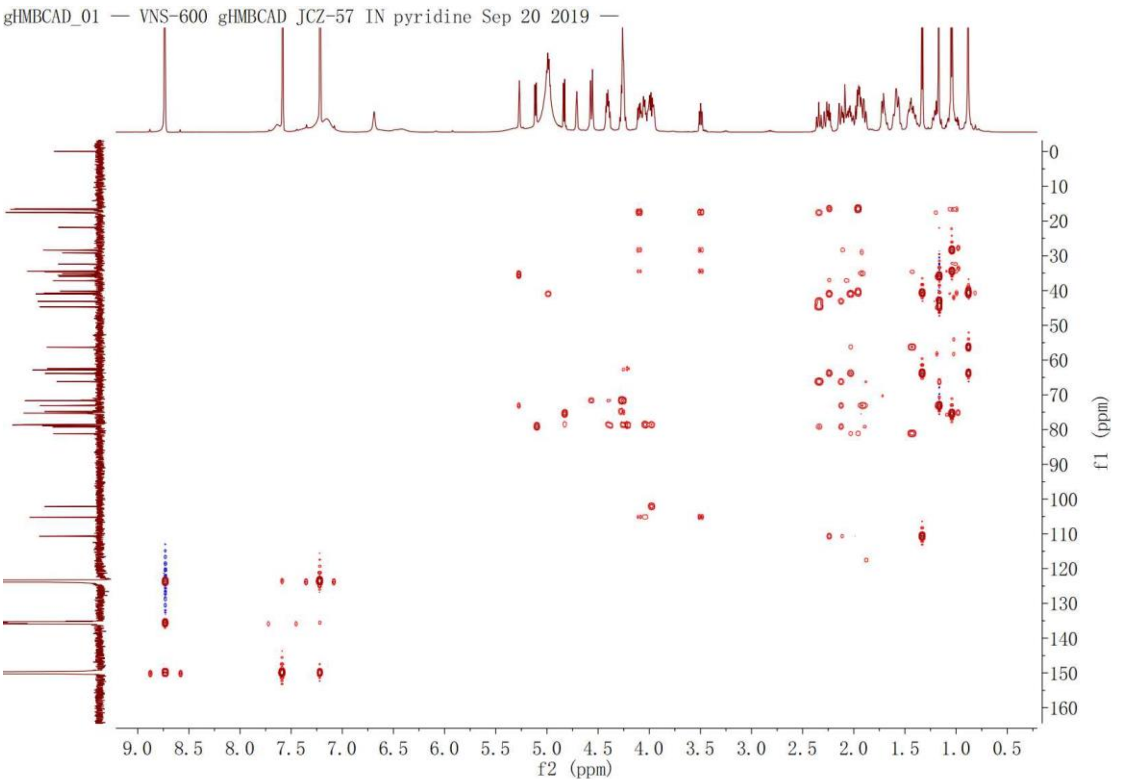

HMBC spectrum of 1.

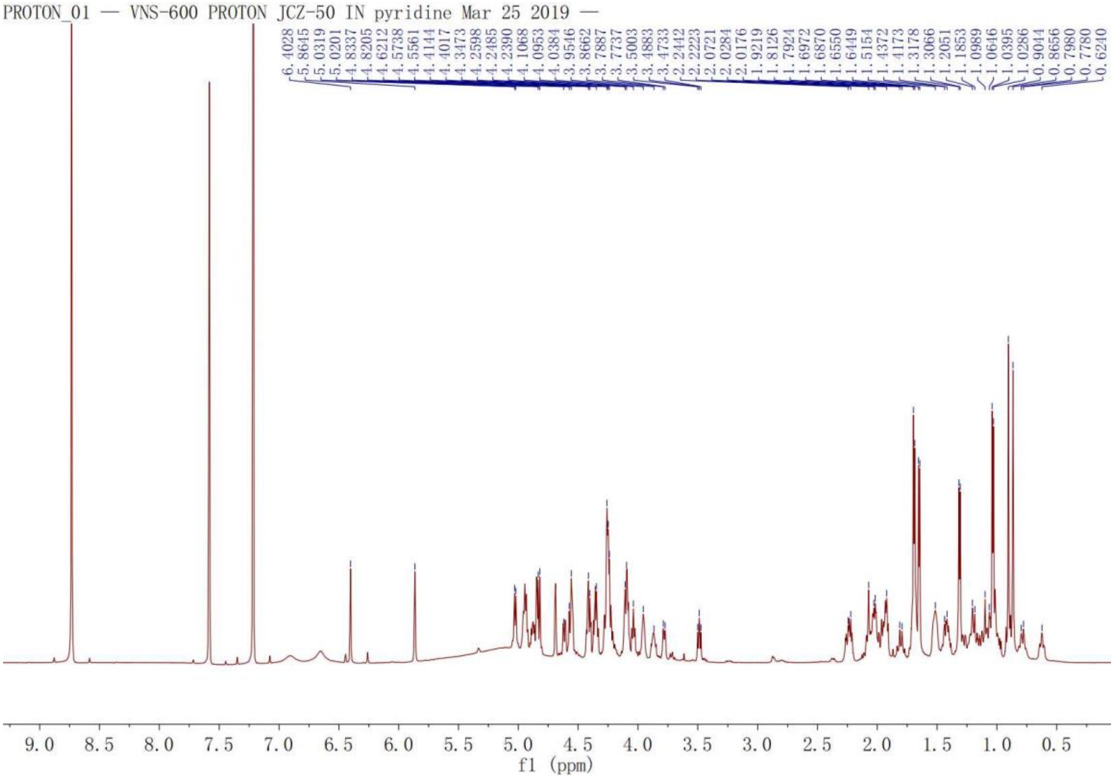

^{1}H NMR spectrum of 2.

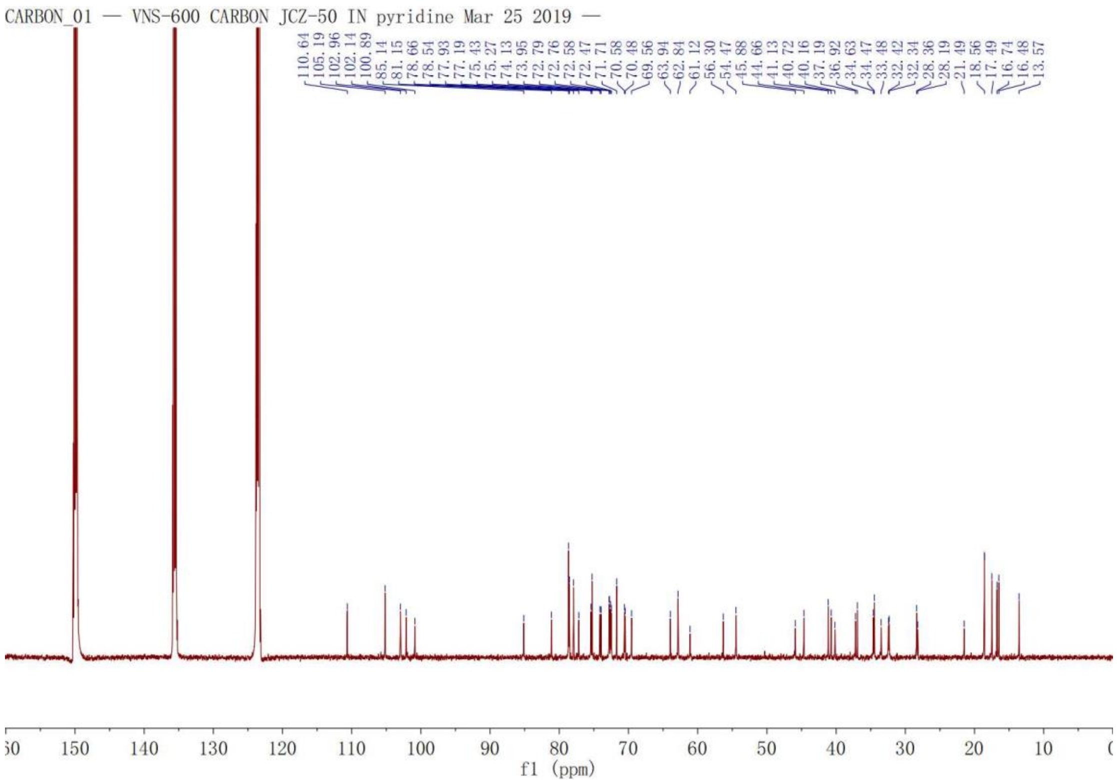

^{13}C NMR spectrum of 2.

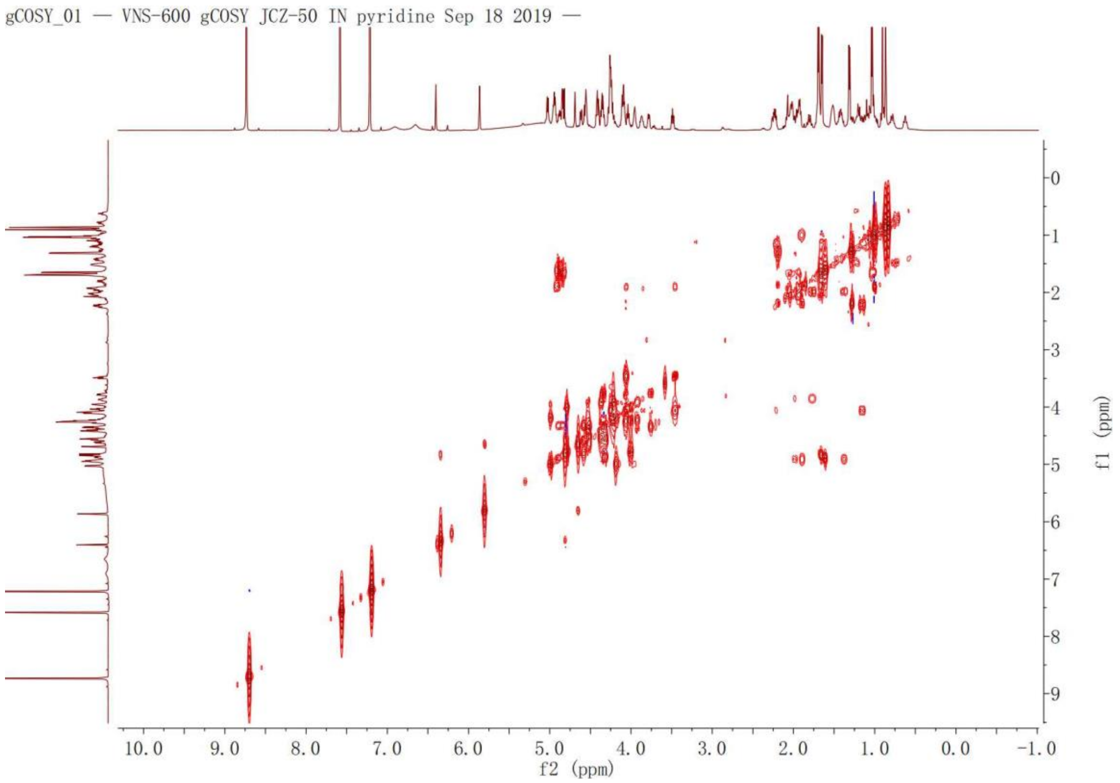

^1H-^1H COSY spectrum of 2.

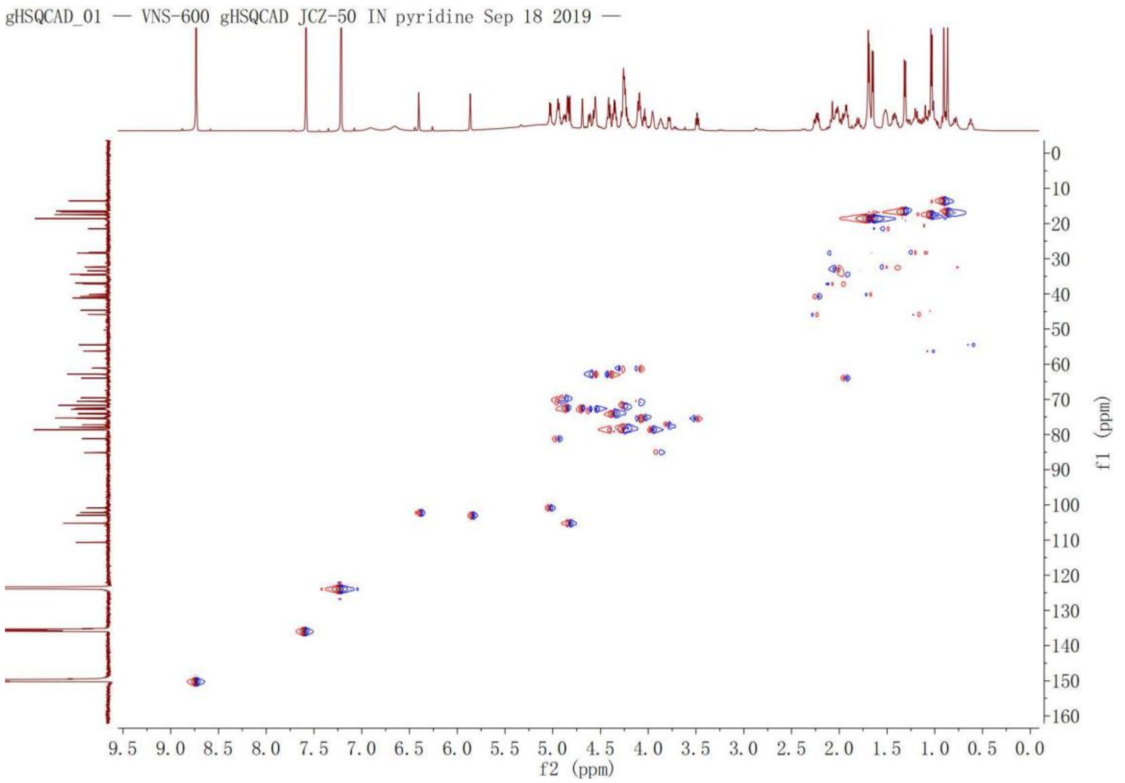

HSQC spectrum of 2.

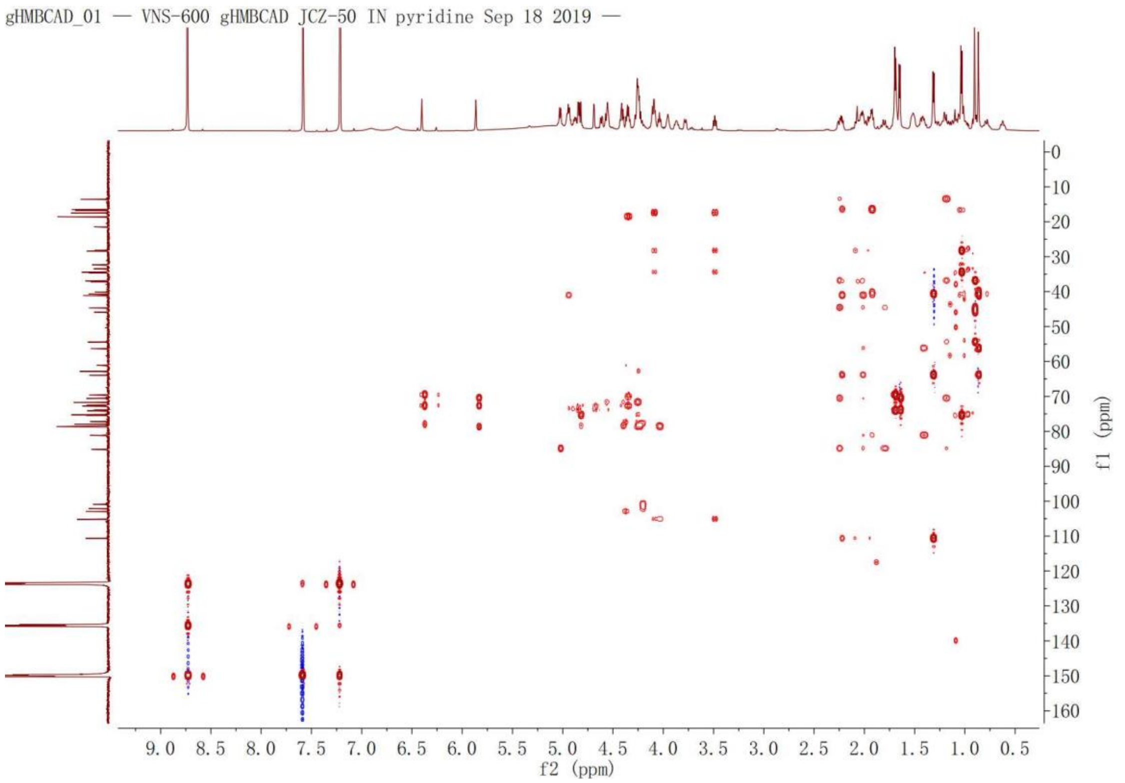

HMBC spectrum of 2.

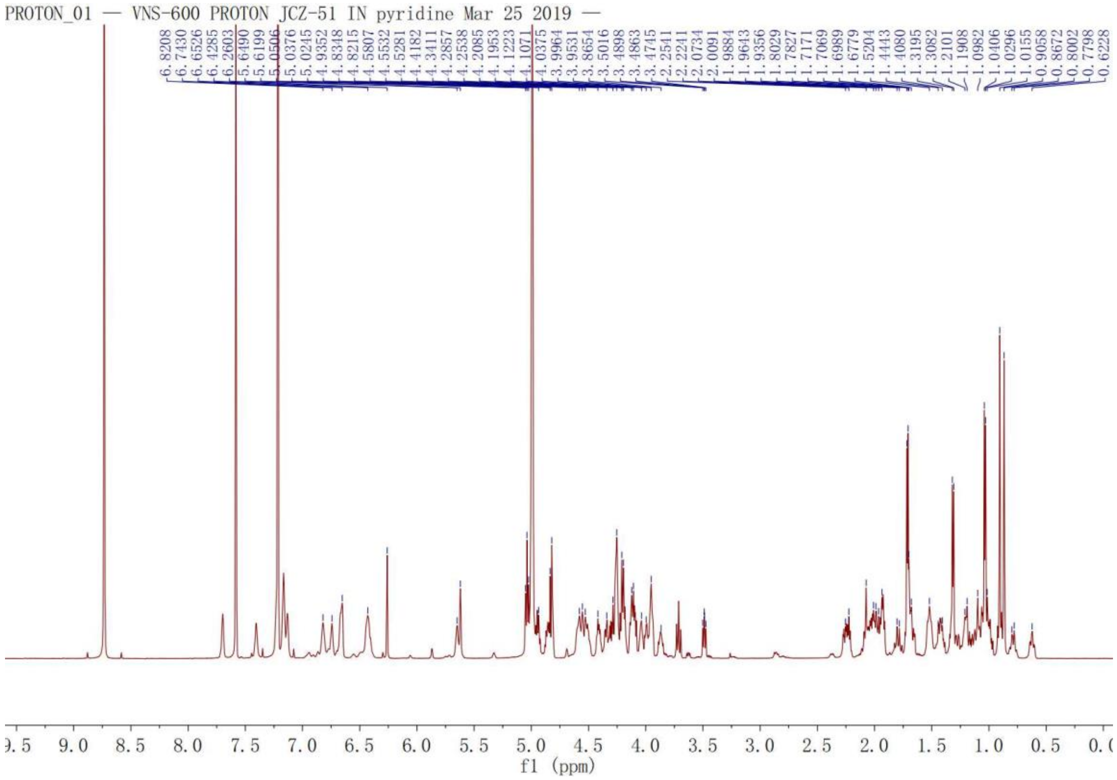

^{1}H NMR spectrum of 3.

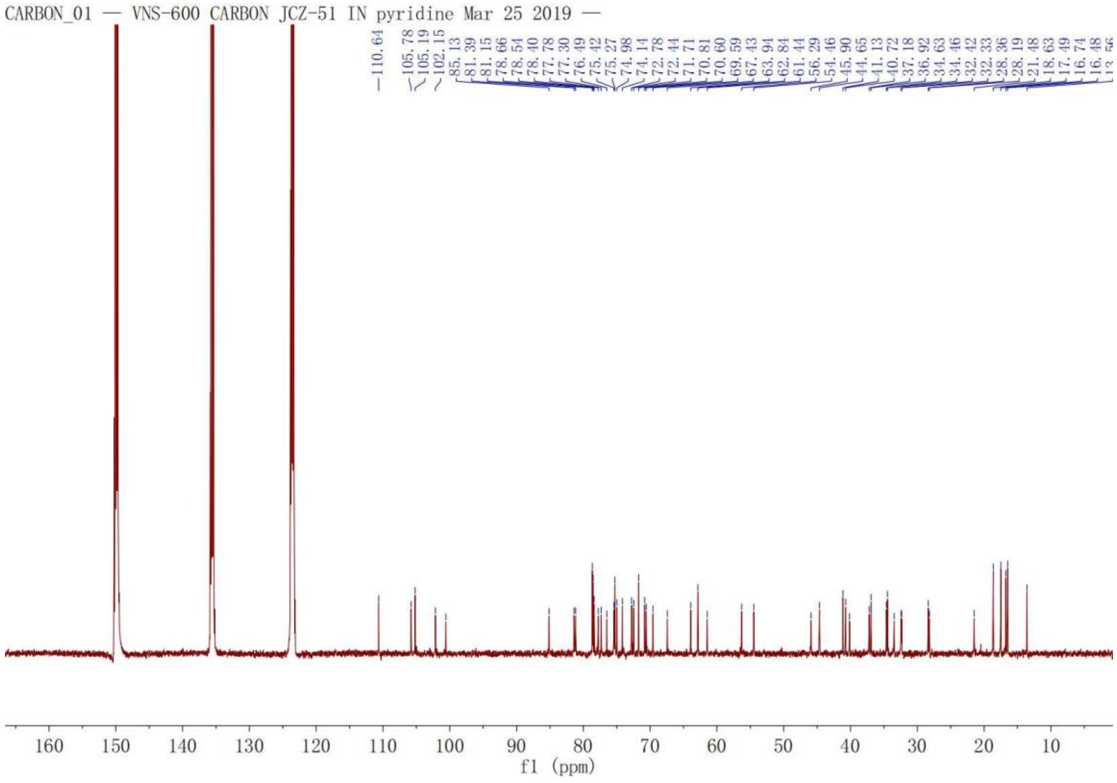

^{13}C NMR spectrum of 3.

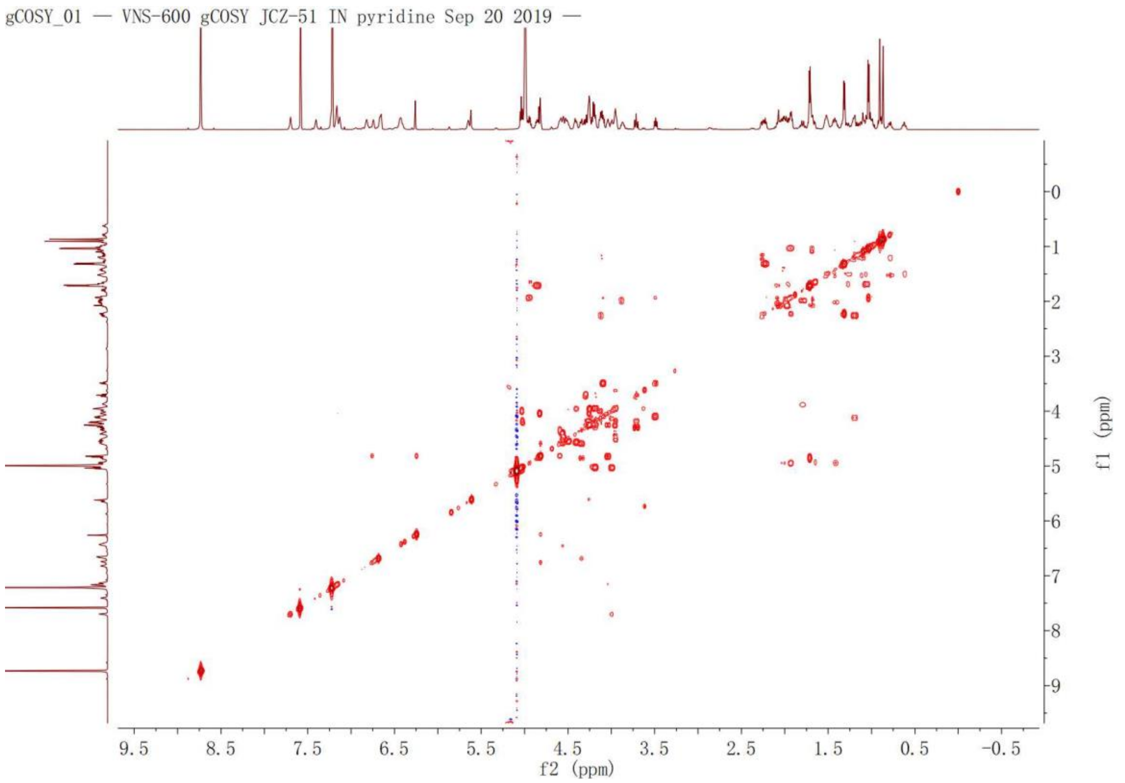

¹H-¹H COSY spectrum of 3.

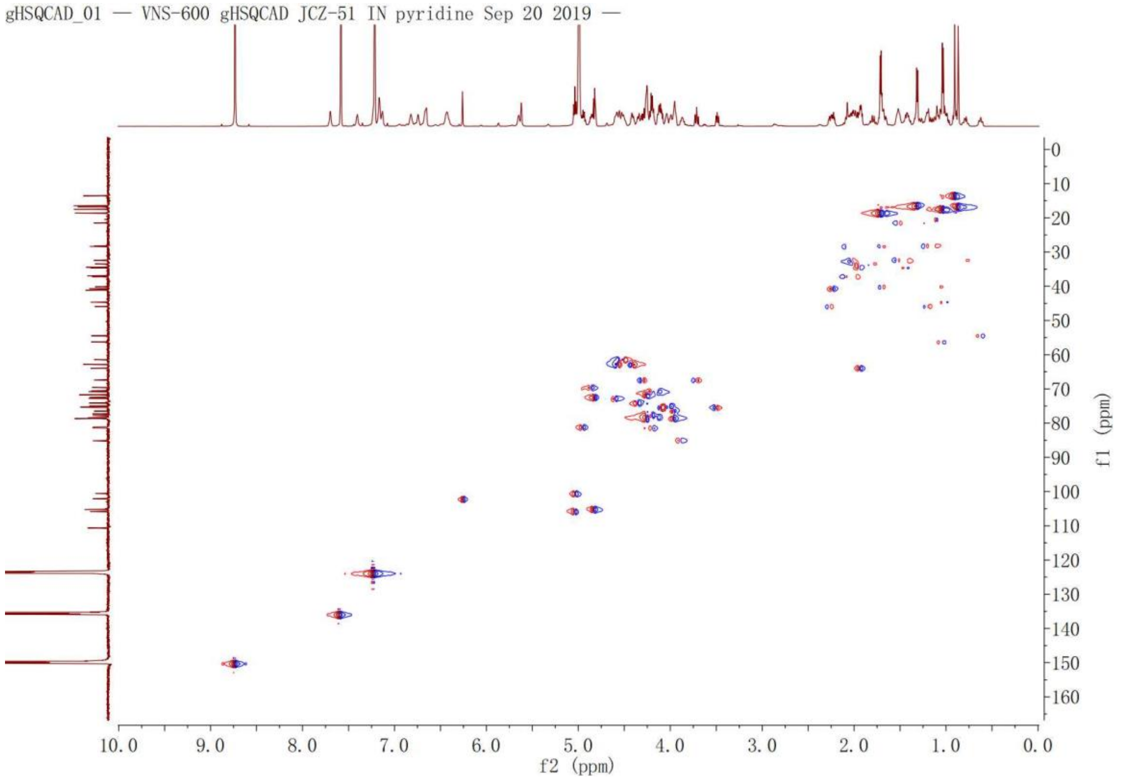

HSQC spectrum of 3.

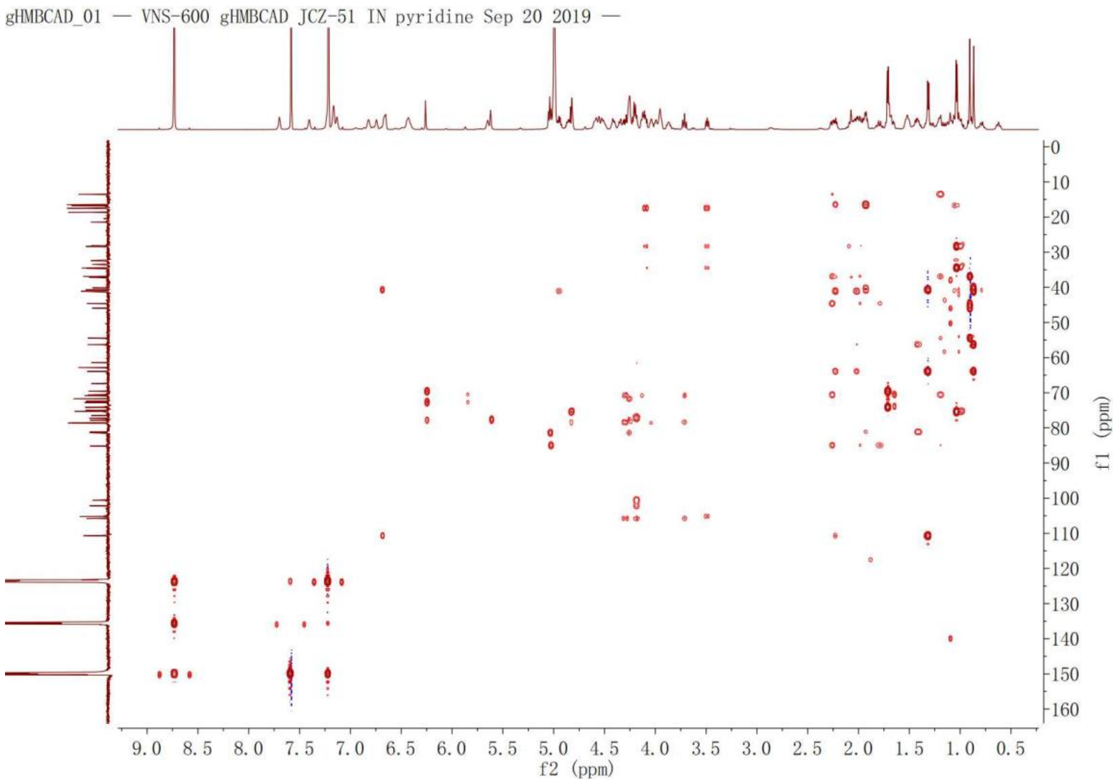

HMBC spectrum of 3.

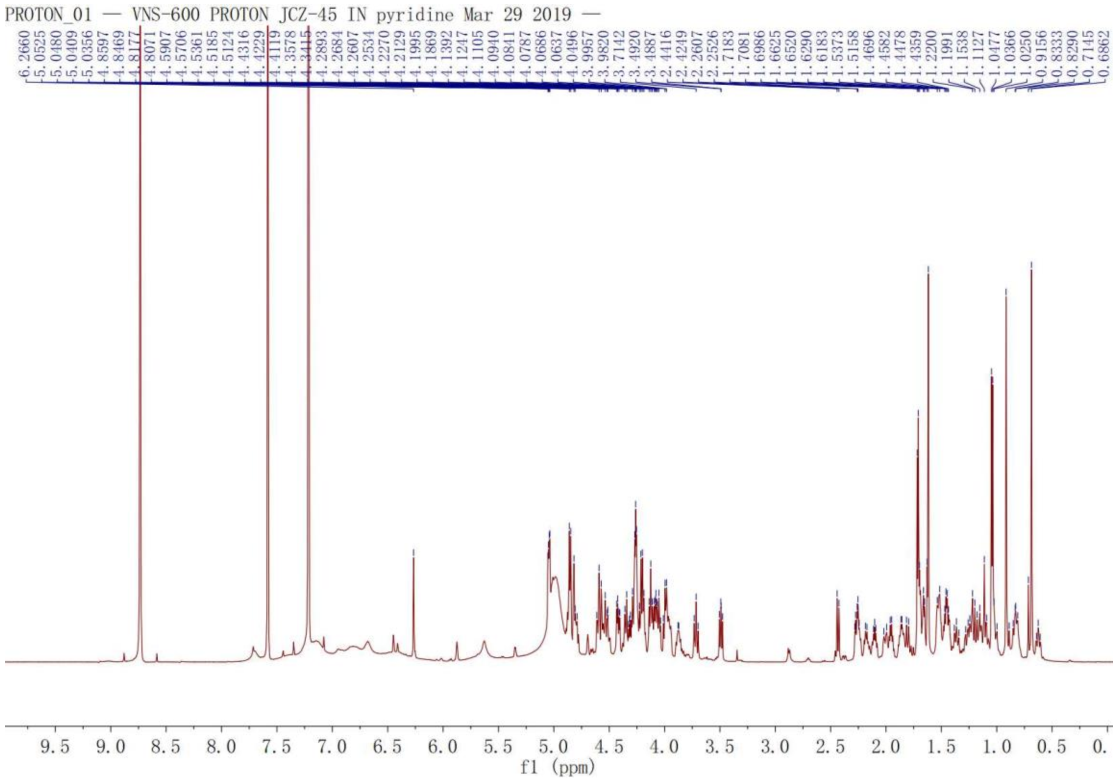

^{1}H NMR spectrum of 4.

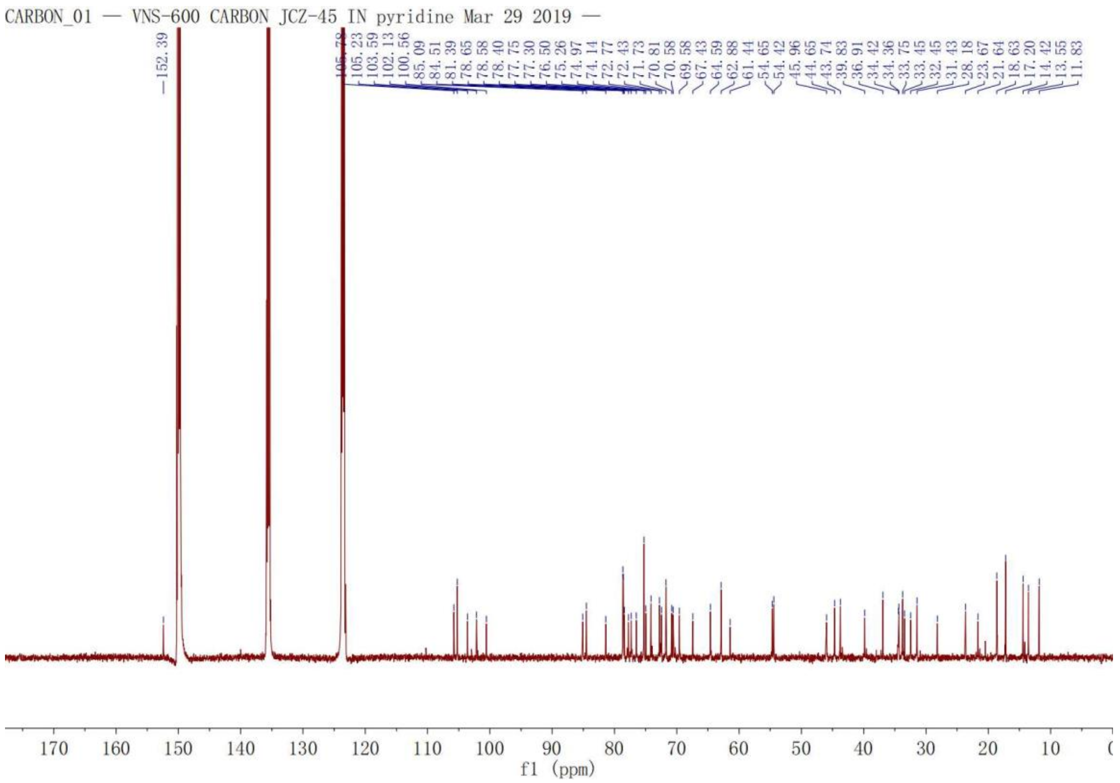

^{13}C NMR spectrum of 4.

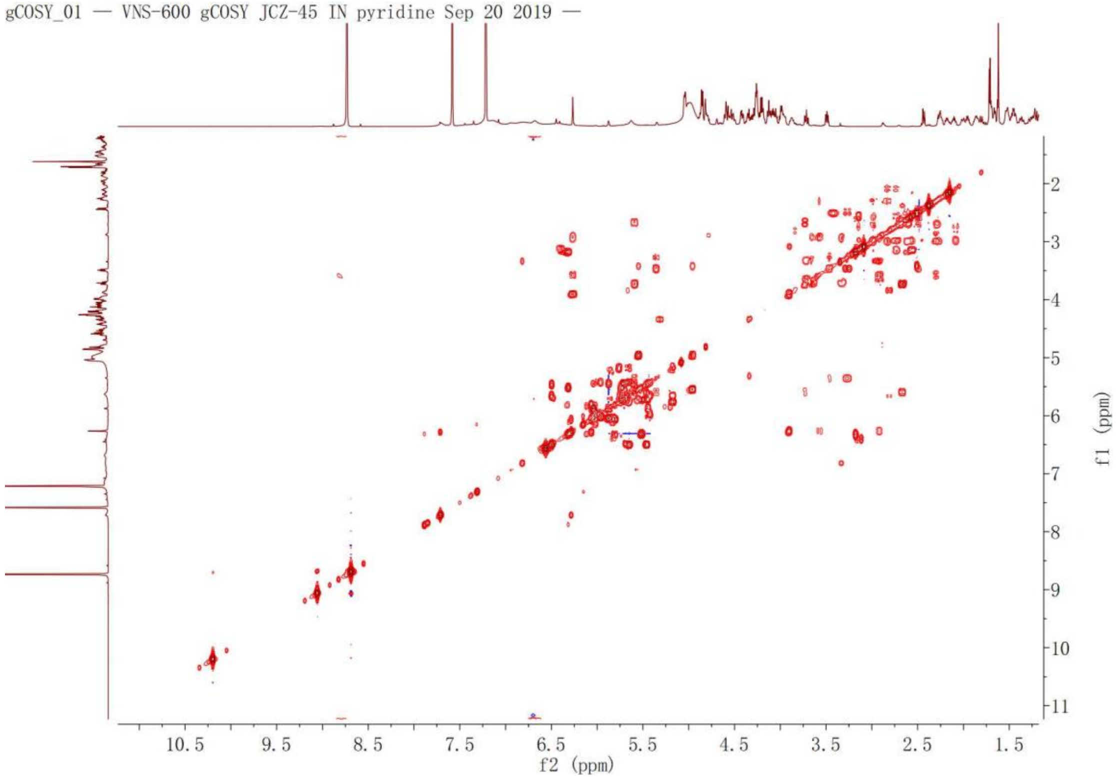

^{1}H-^{1}H COSY spectrum of 4.

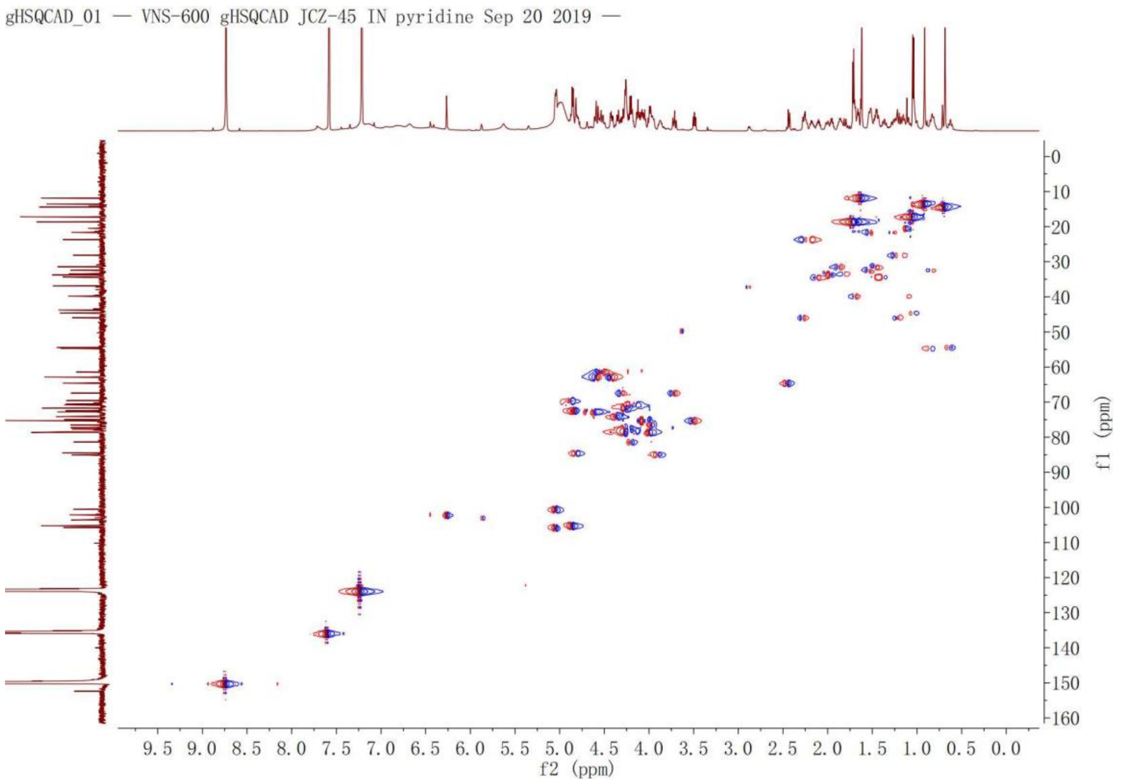

HSQC spectrum of 4.

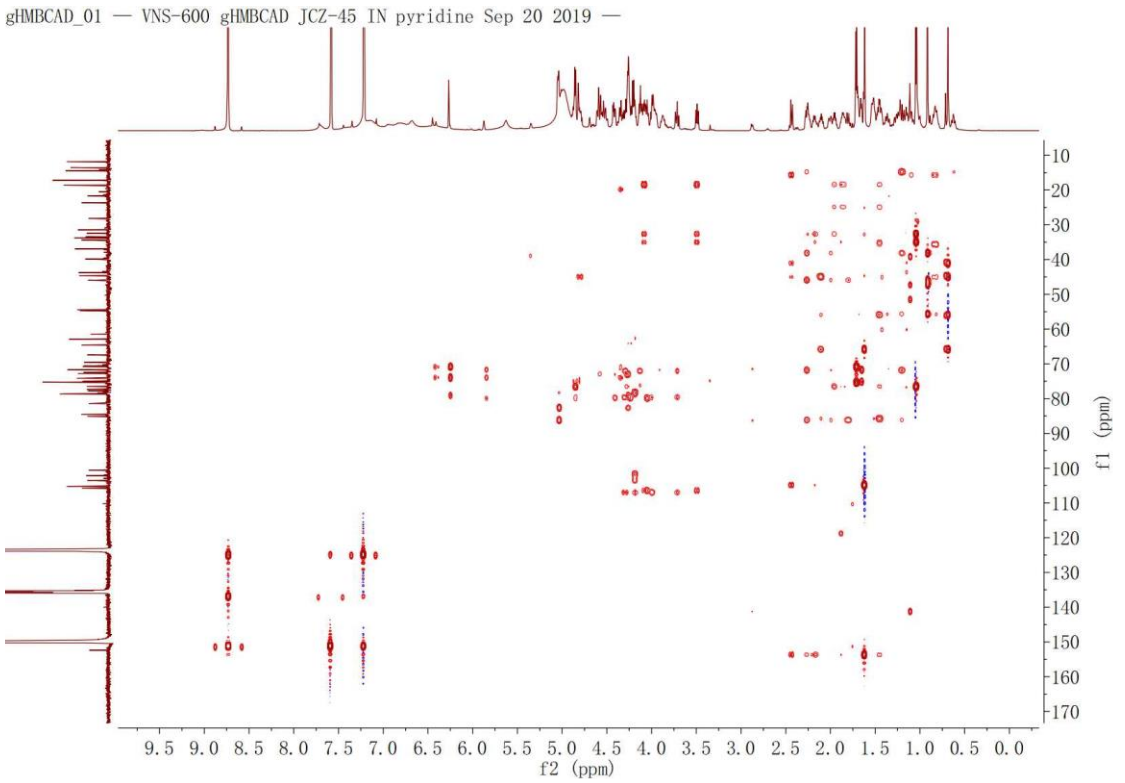

HMBC spectrum of 4.

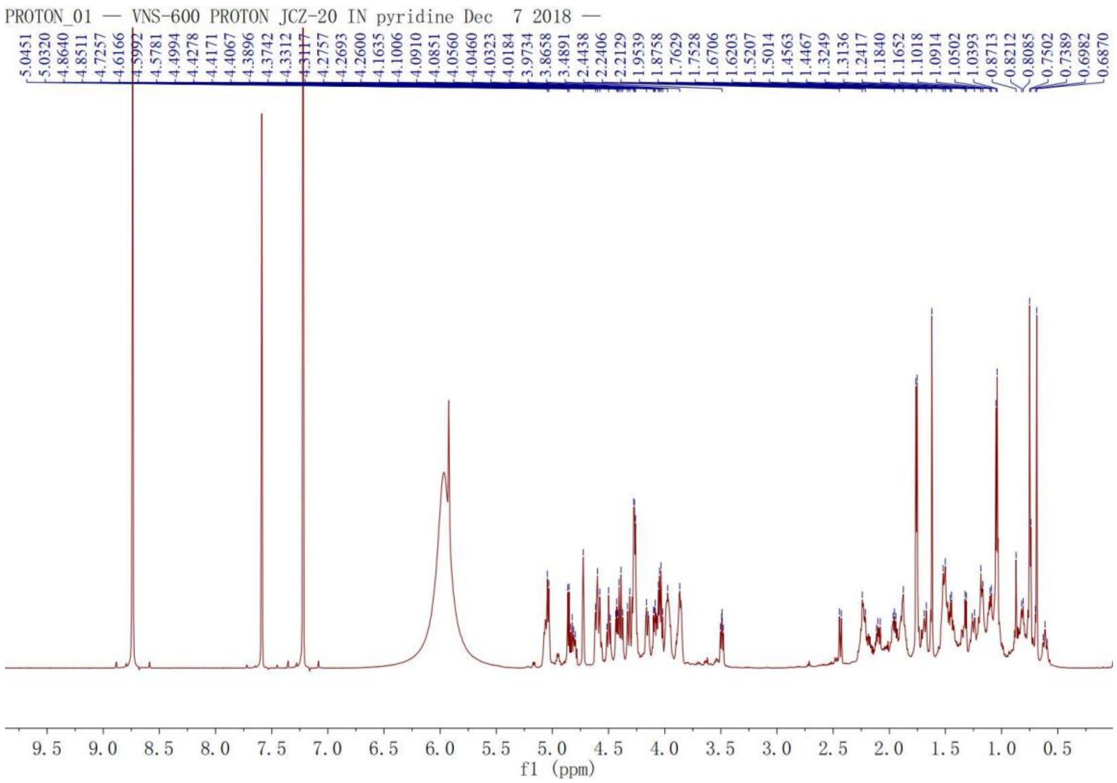

^{1}H NMR spectrum of 5.

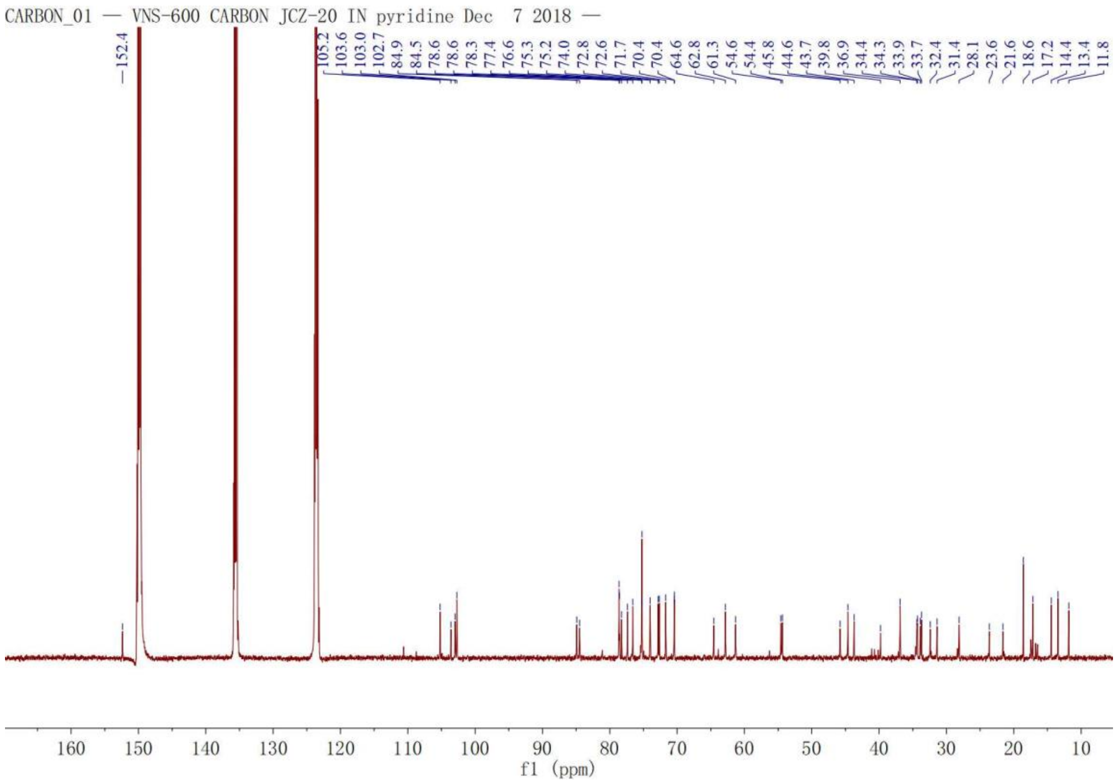

^{13}C NMR spectrum of 5.

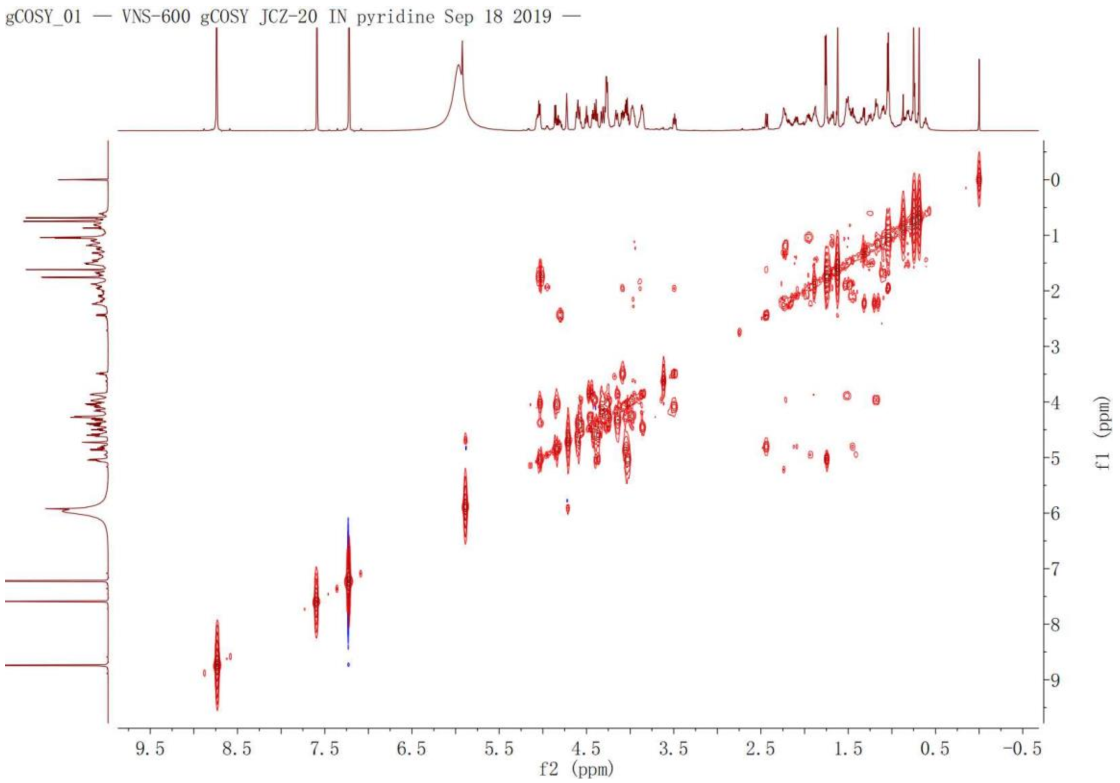

¹H-¹H COSY spectrum of 5.

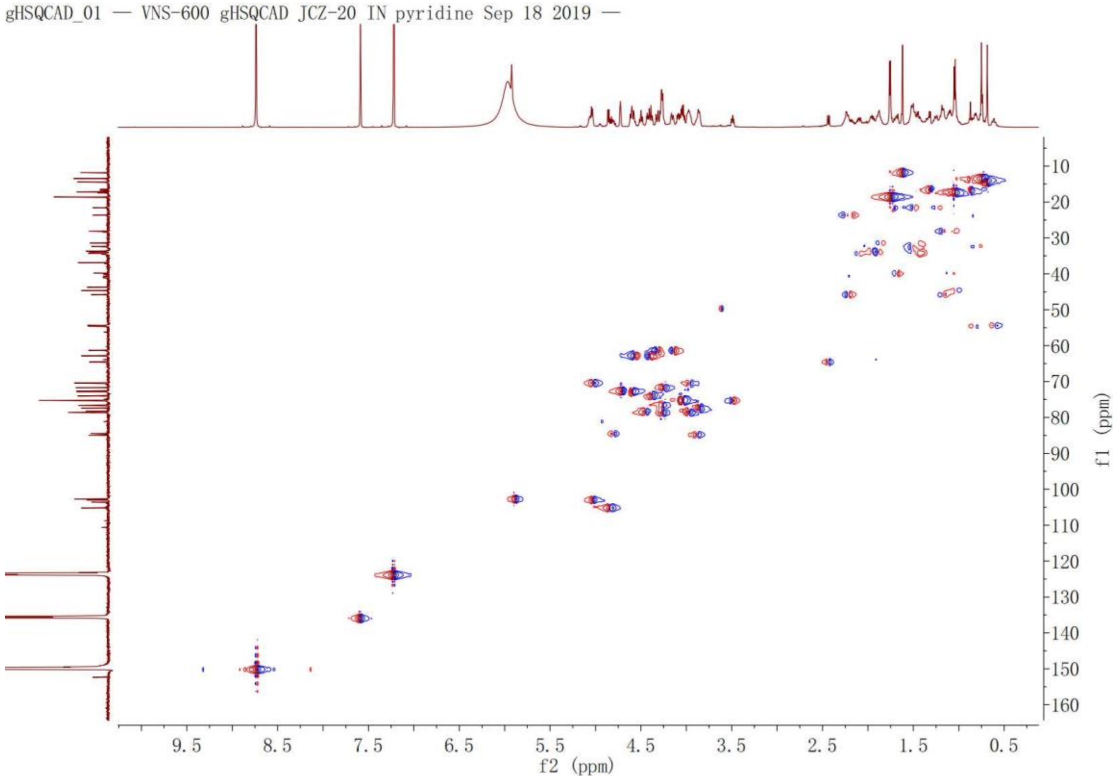

HSQC spectrum of 5.

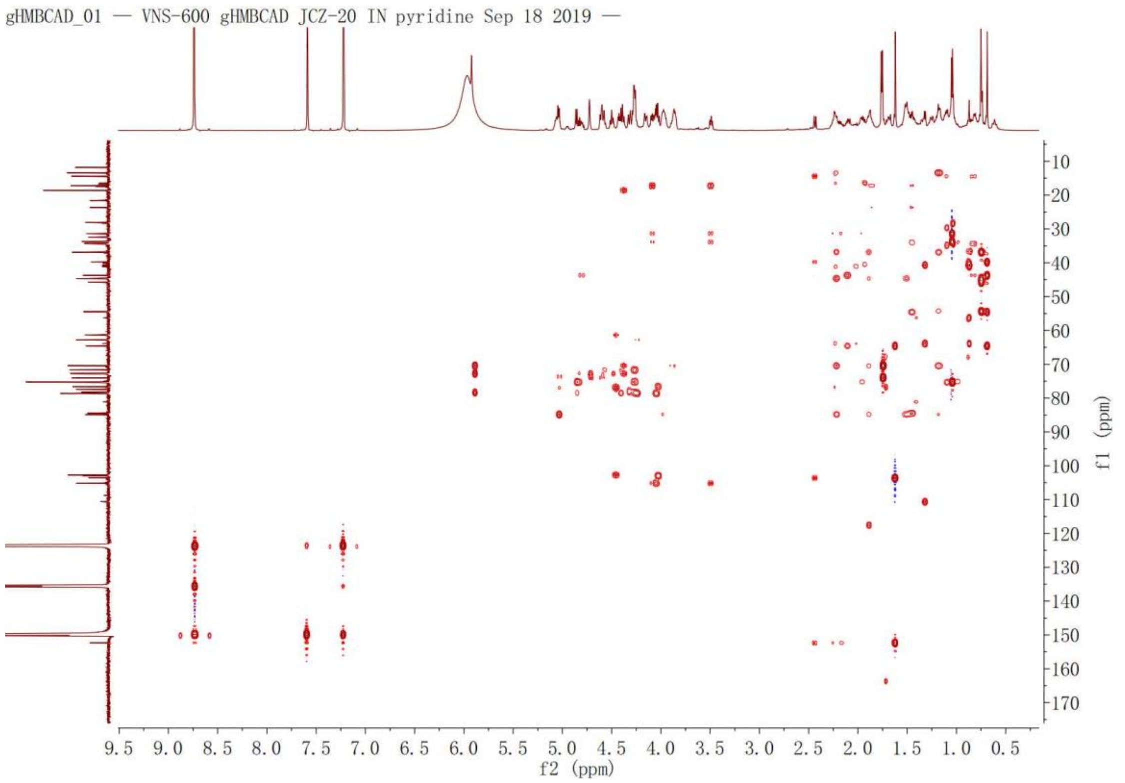

HMBC spectrum of **5**

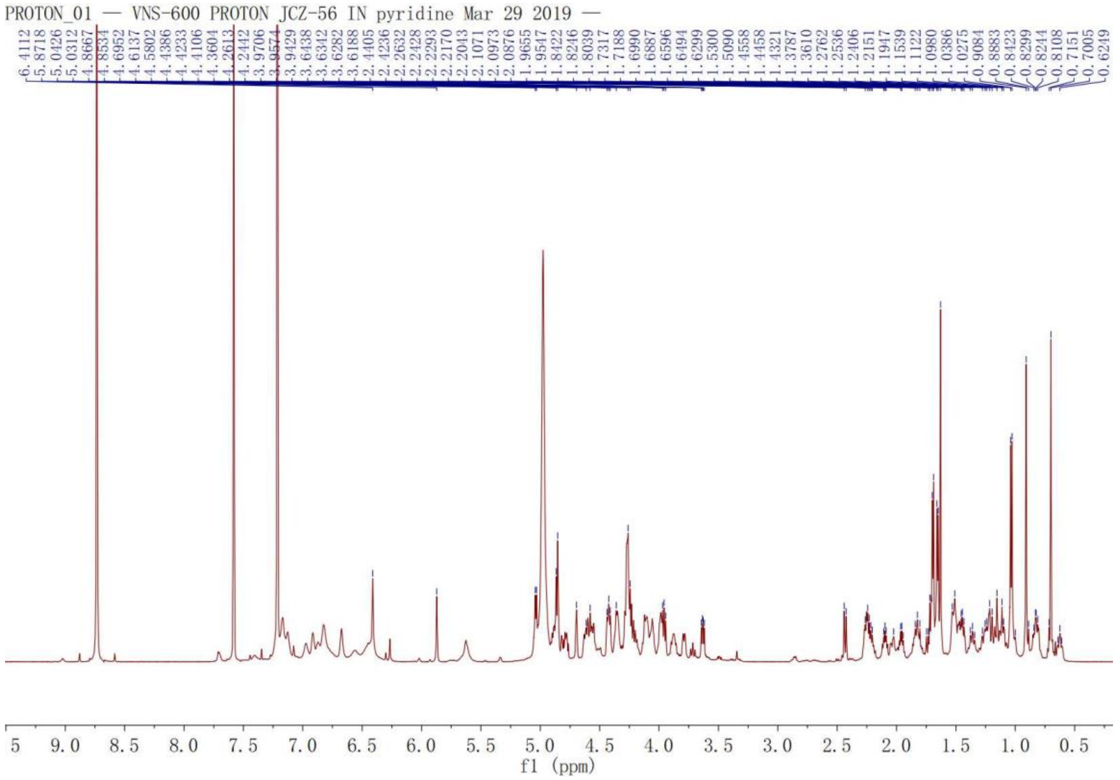

^{1}H NMR spectrum of 6.

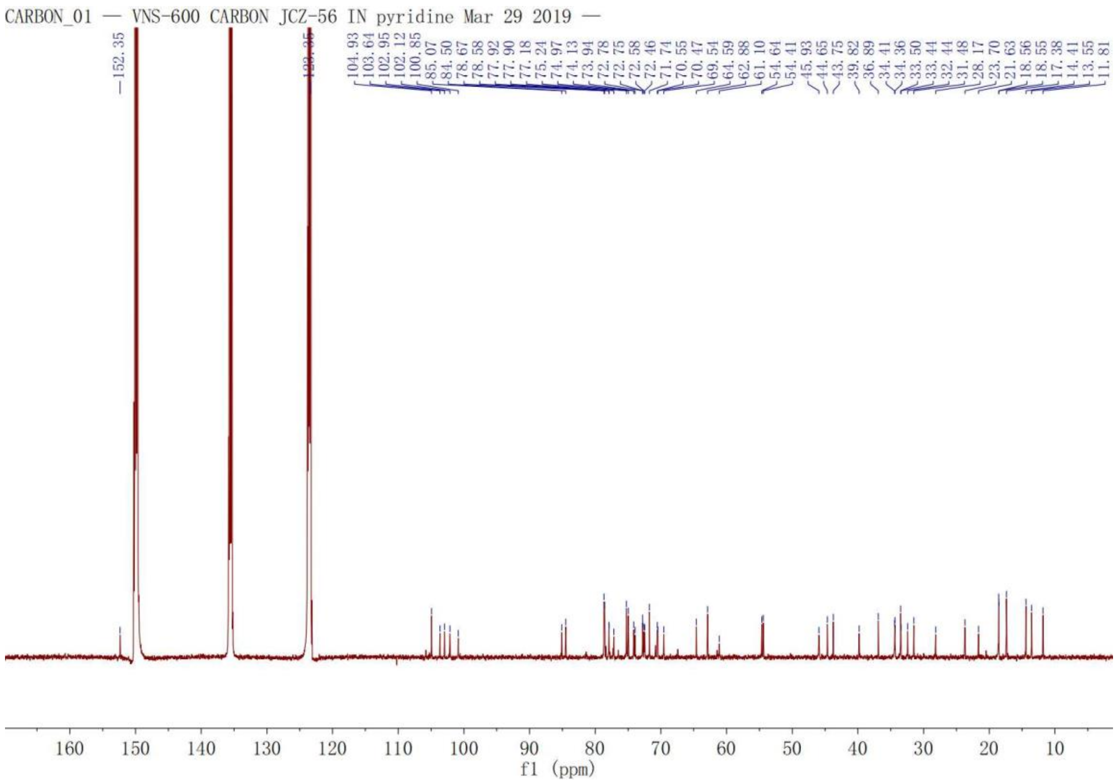

^{13}C NMR spectrum of **6**

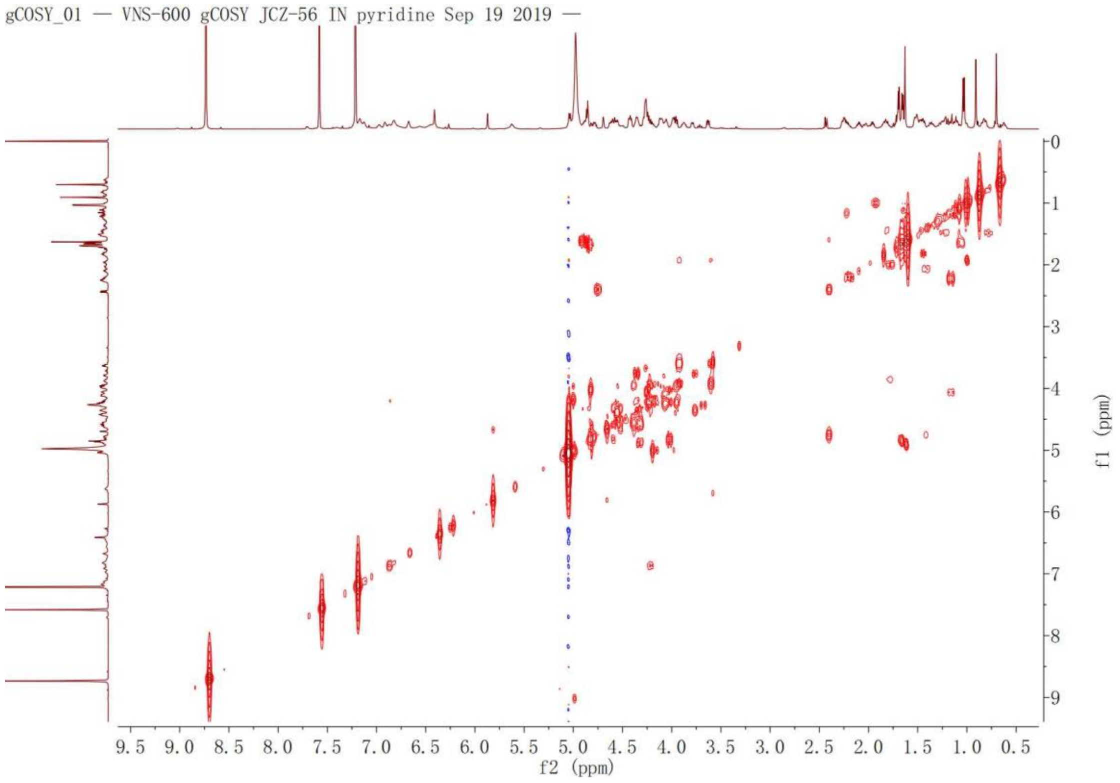

^{1}H-^{1}H COSY spectrum of 6.

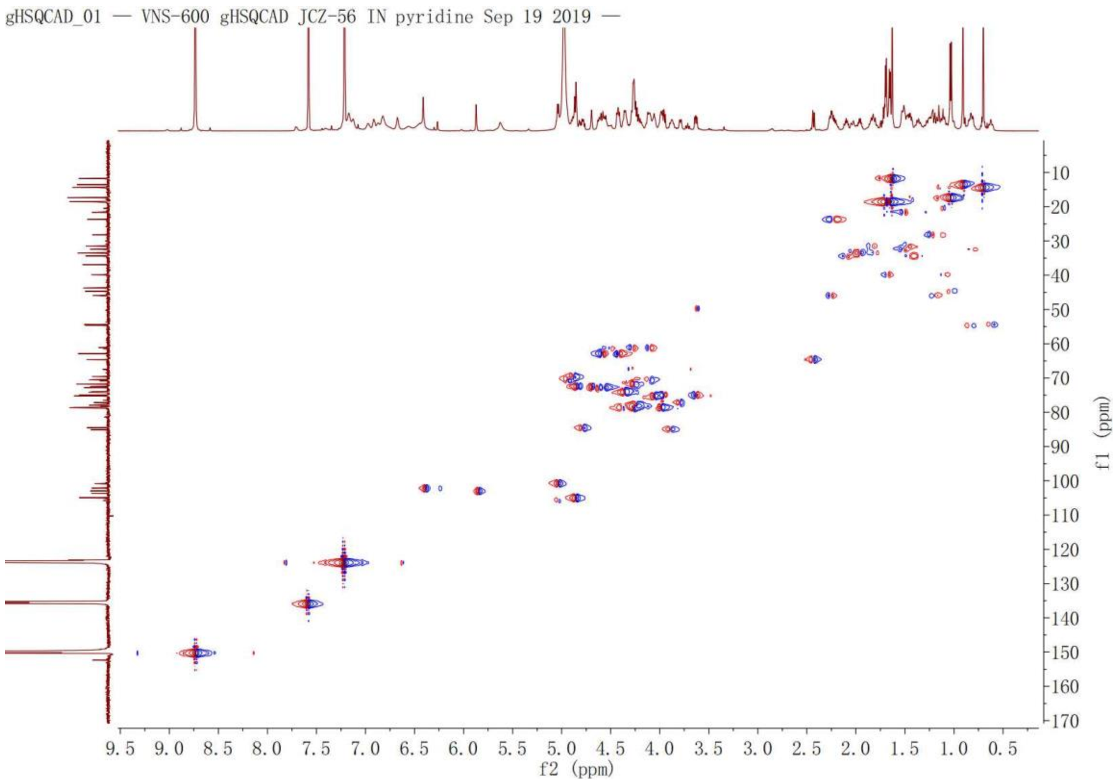

HSQC spectrum of **6**

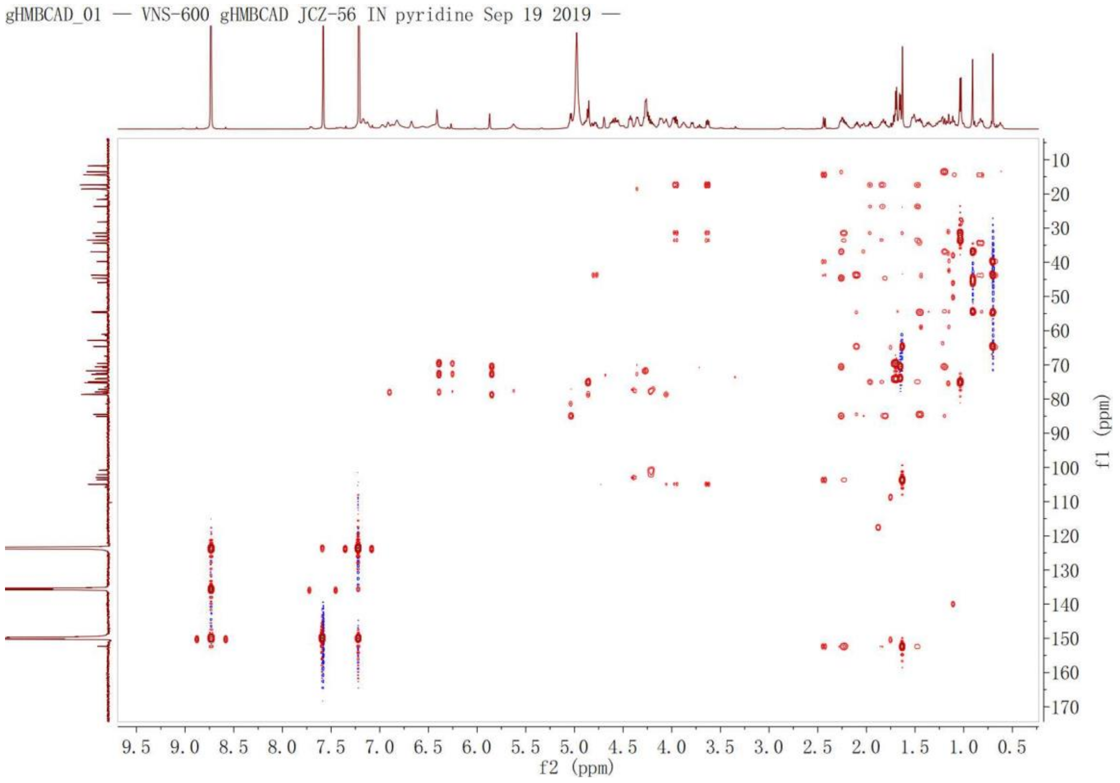

HMBC spectrum of 6.

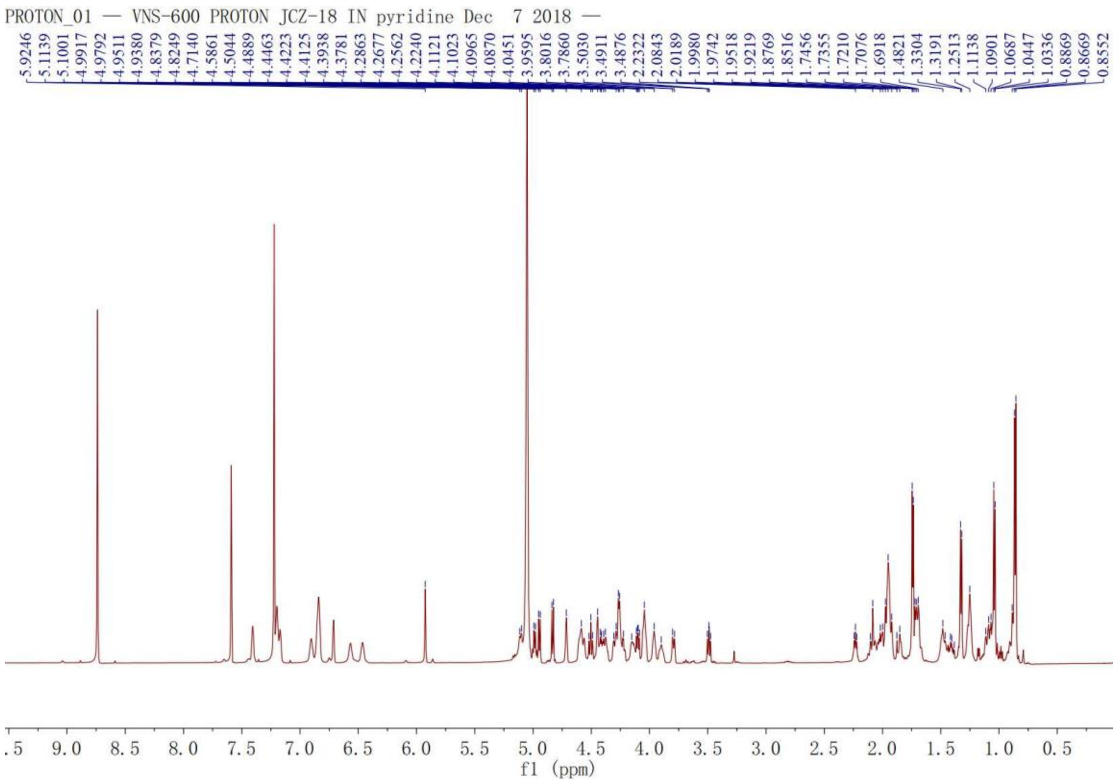

^{1}H NMR spectrum of 7.

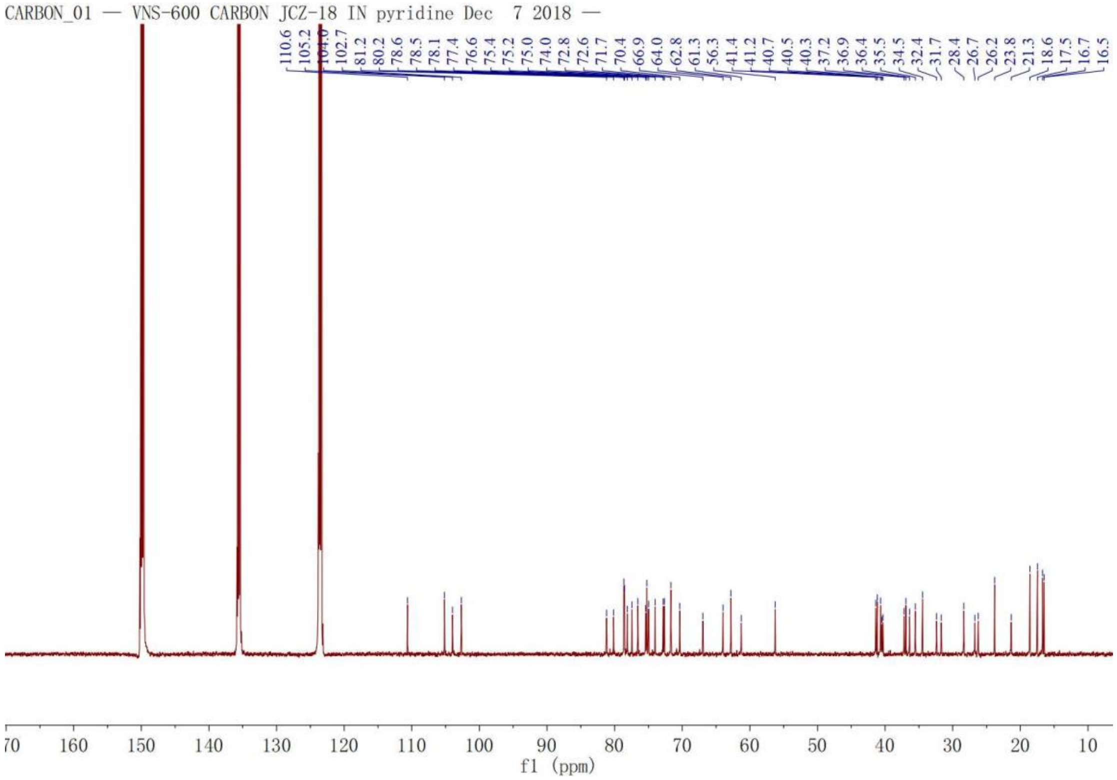

^{13}C NMR spectrum of 7.

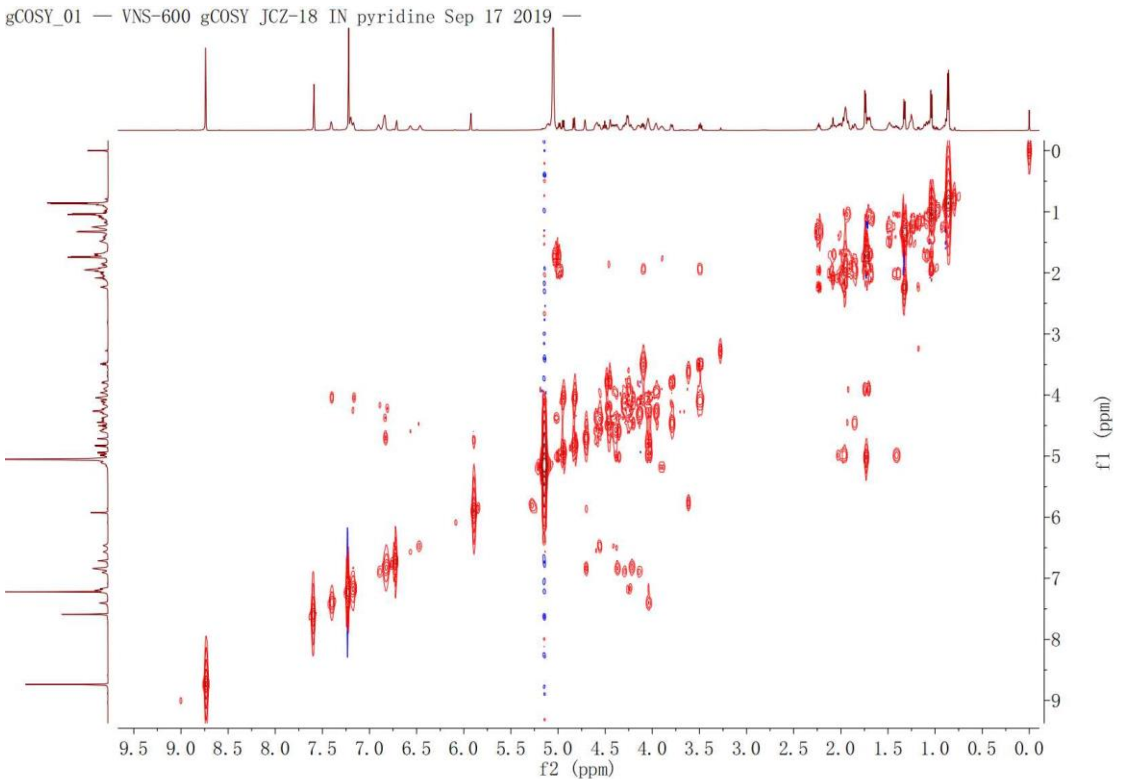

^{1}H-^{1}H COSY spectrum of 7.

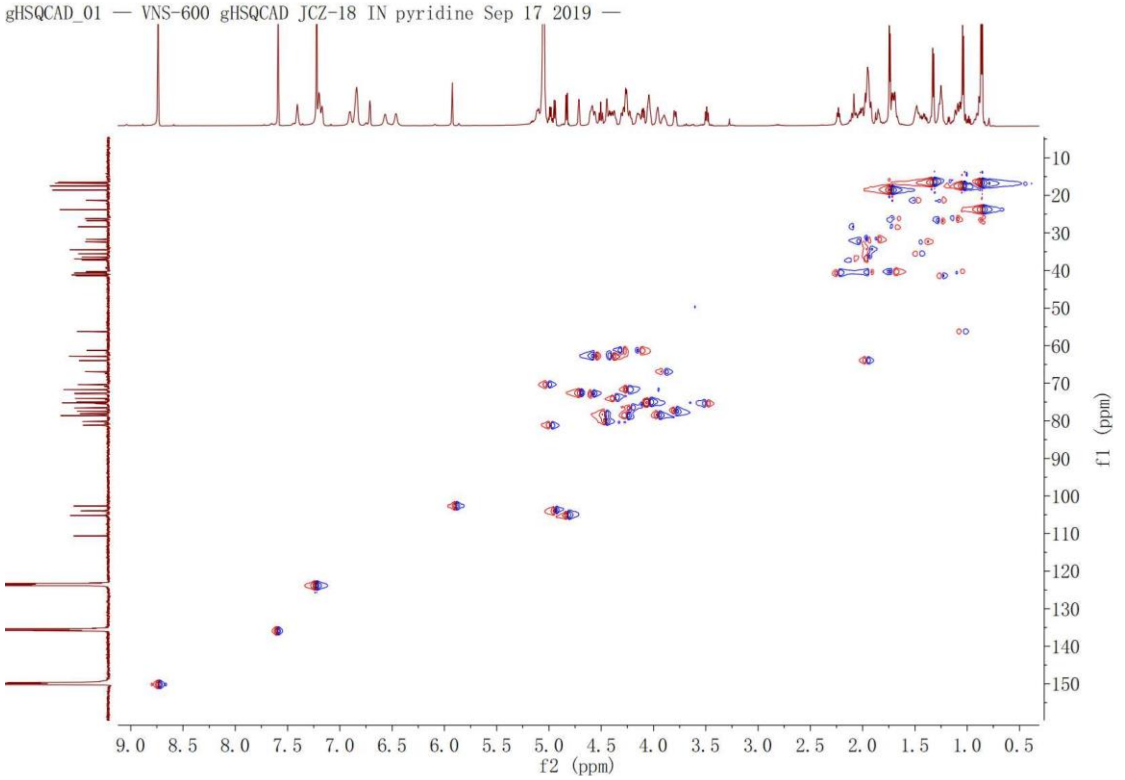

HSQC spectrum of 7.

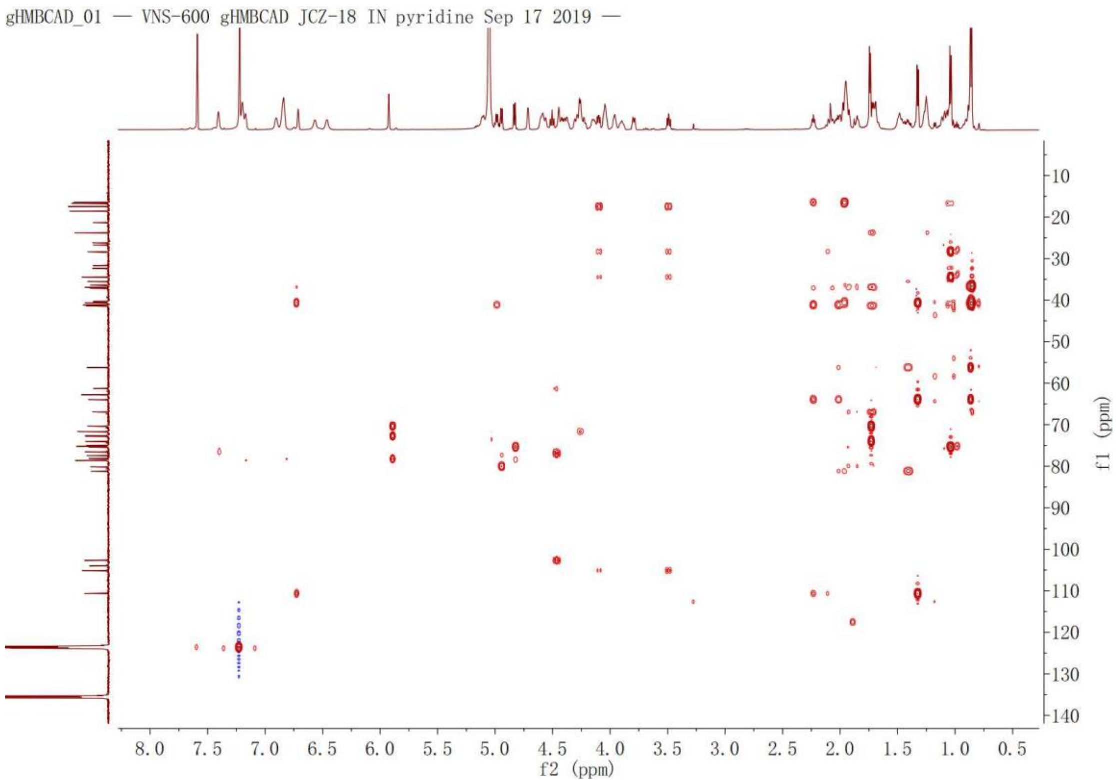

HMBC spectrum of 7.

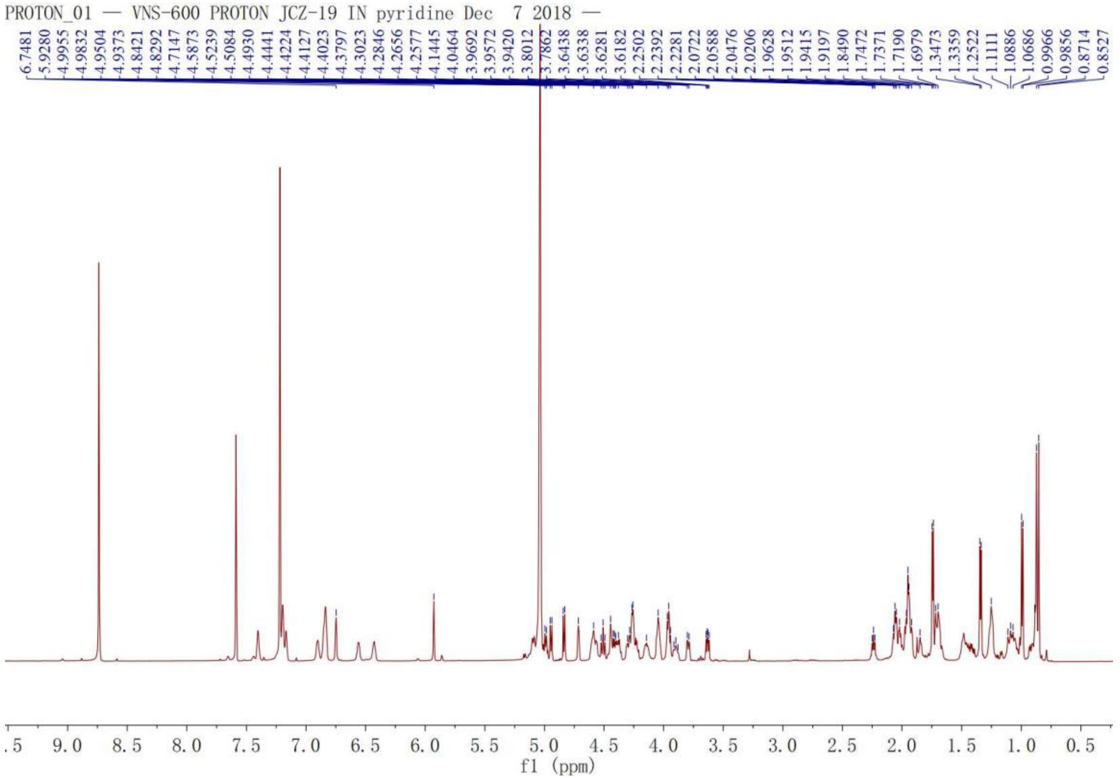

^{1}H NMR spectrum of 8.

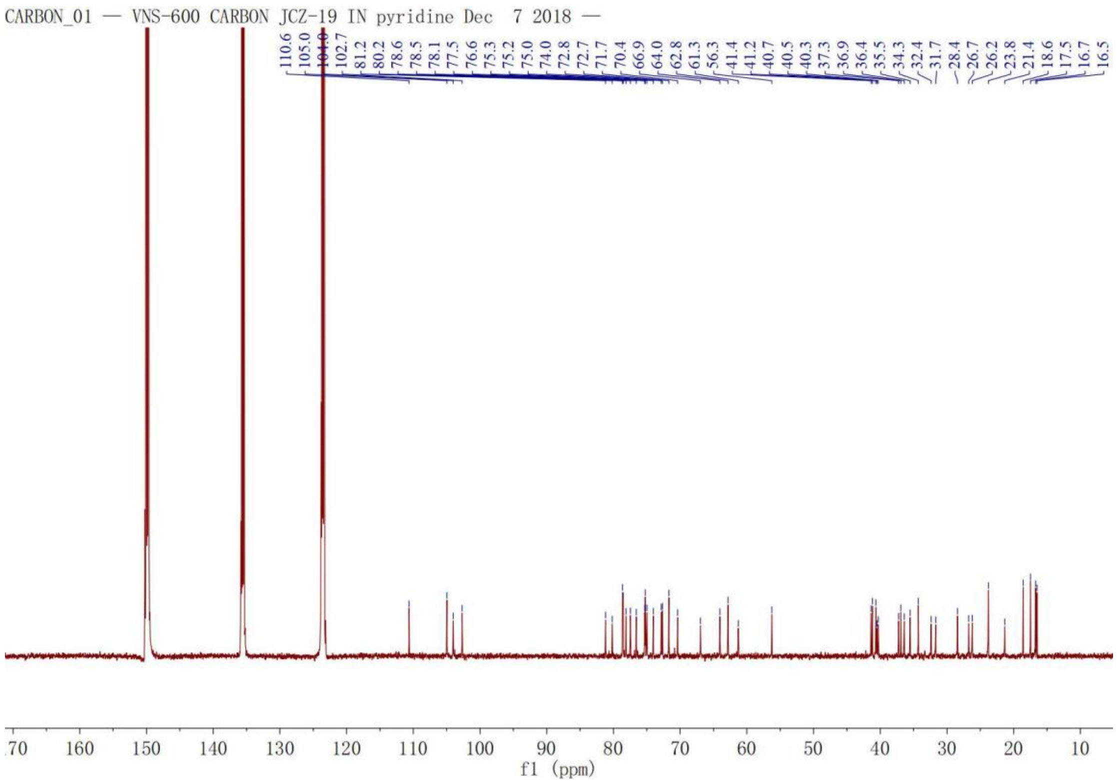

^{13}C NMR spectrum of 8.

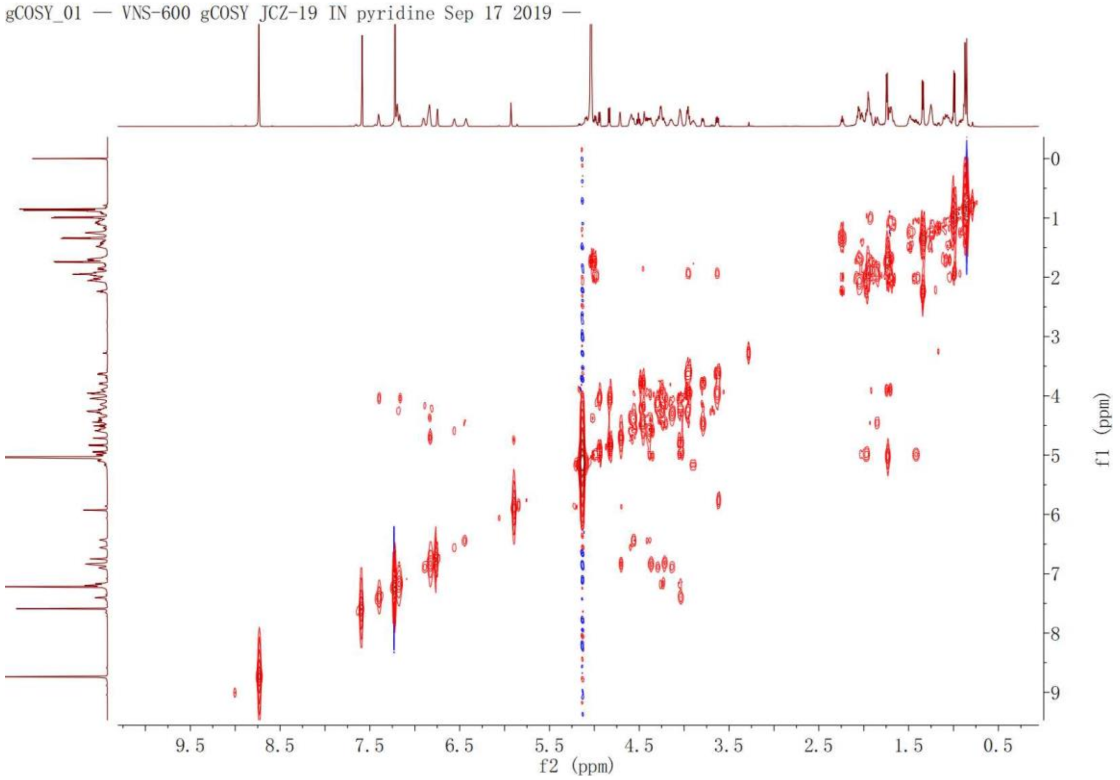

^{1}H-^{1}H COSY spectrum of 8.

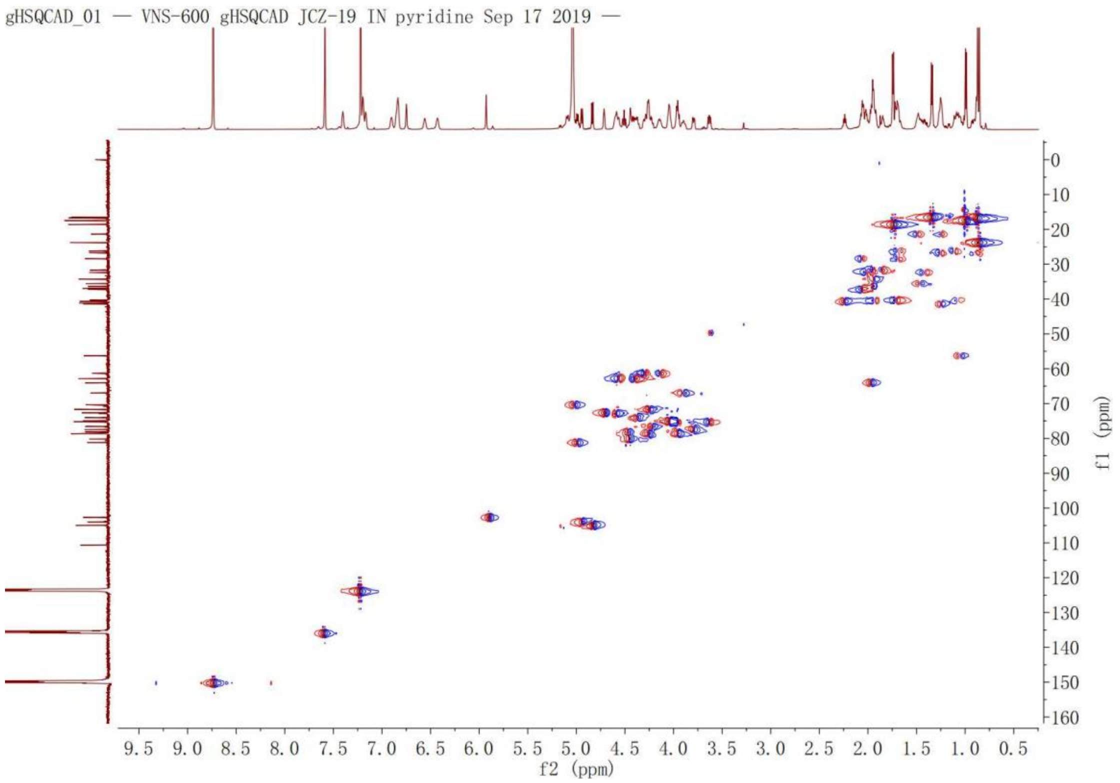

HSQC spectrum of 8.

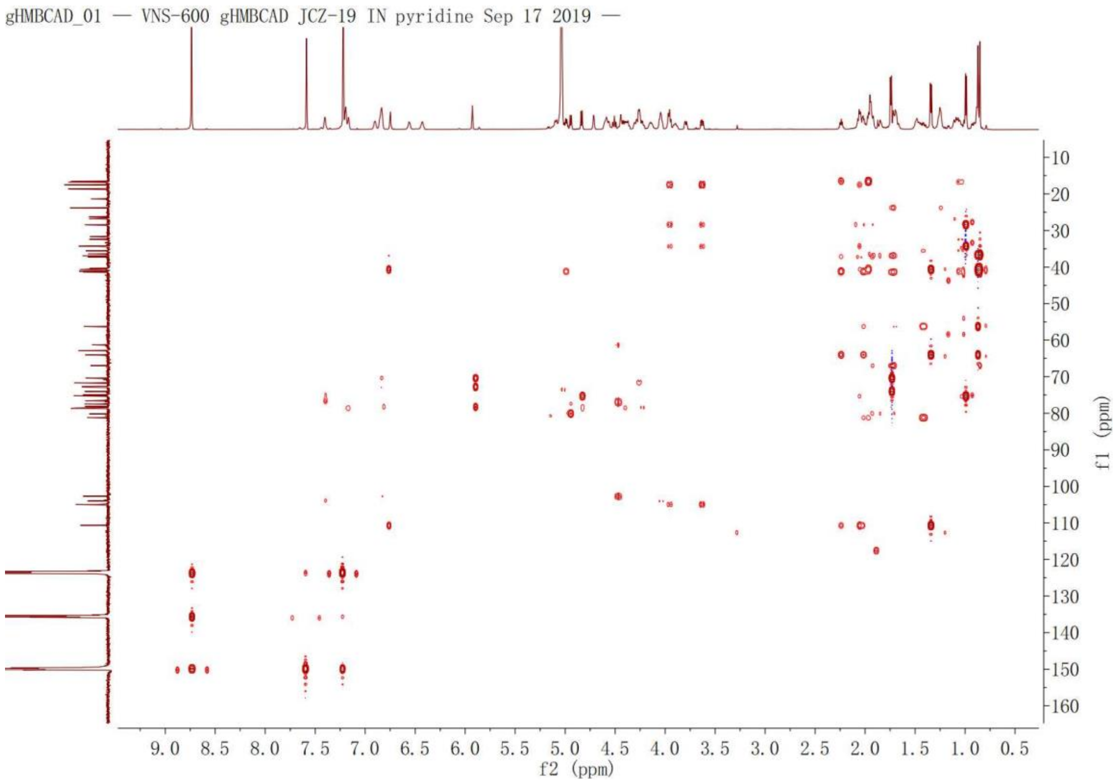

HMBC spectrum of 8.

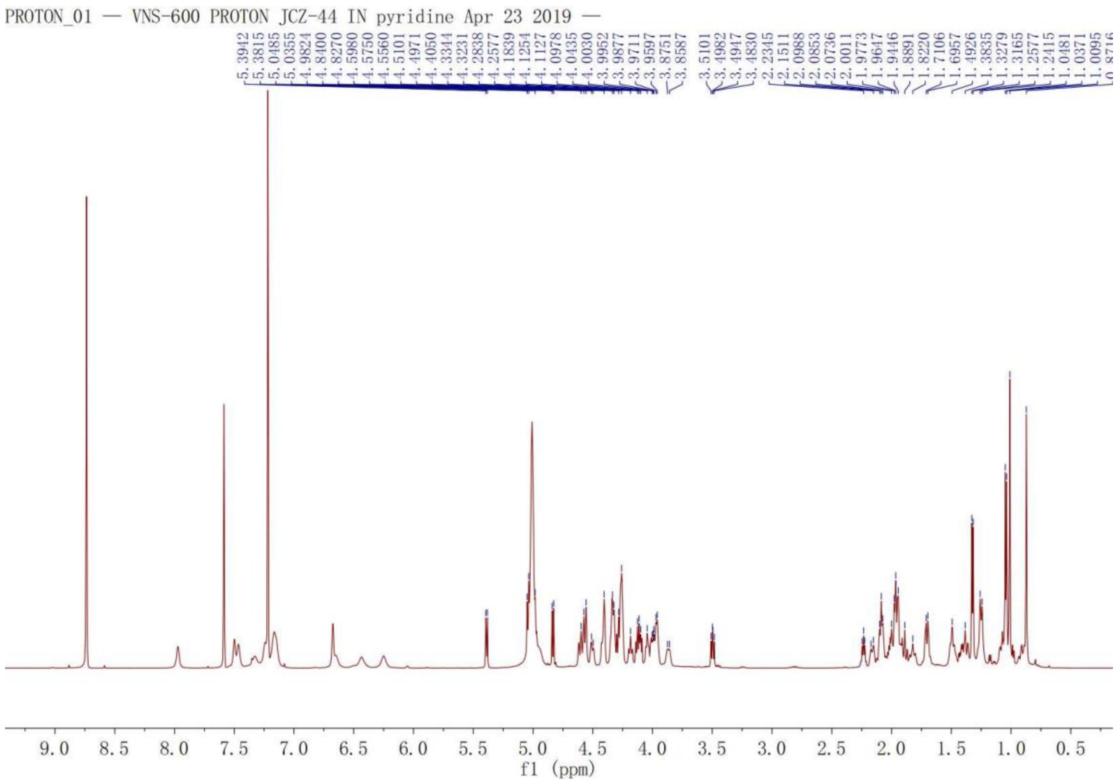

^{1}H NMR spectrum of 9.

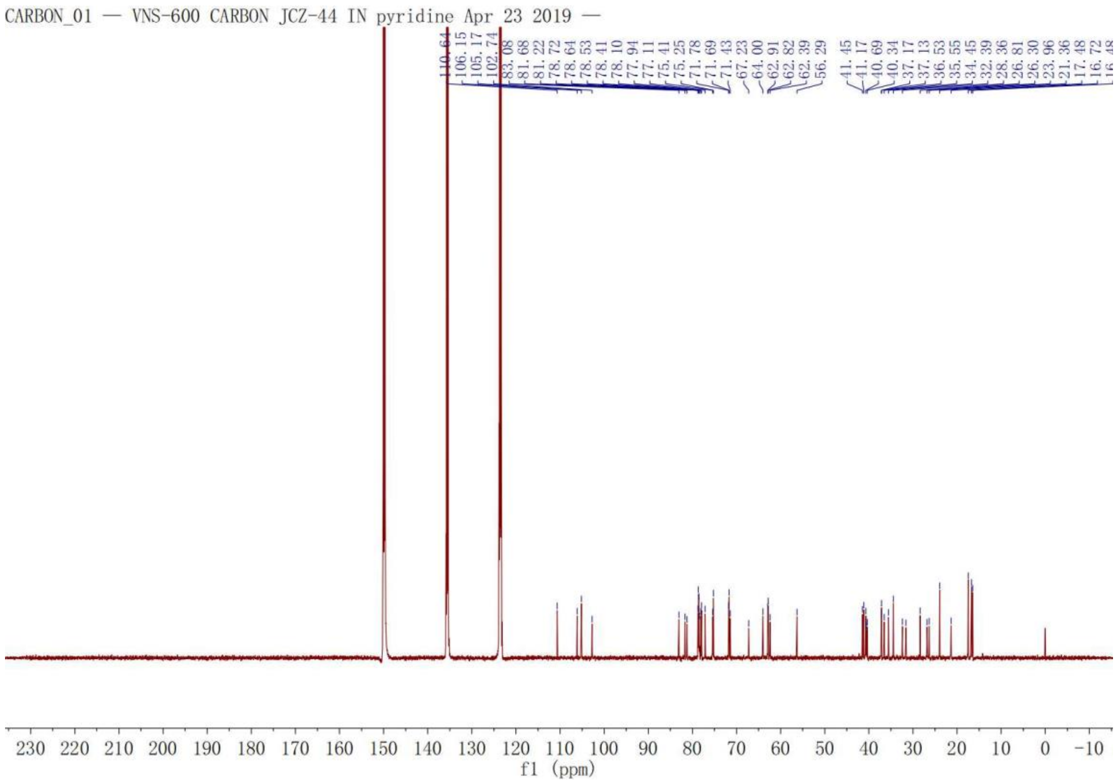

^{13}C NMR spectrum of 9.

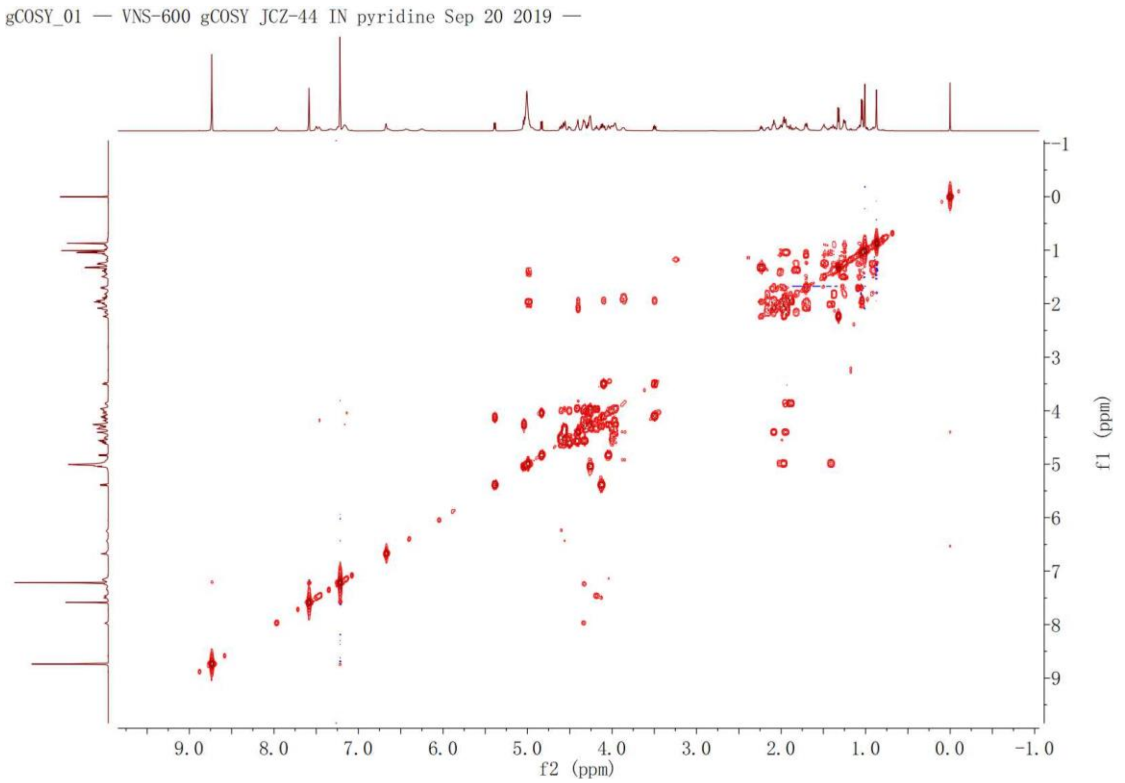

^{1}H-^{1}H COSY spectrum of 9.

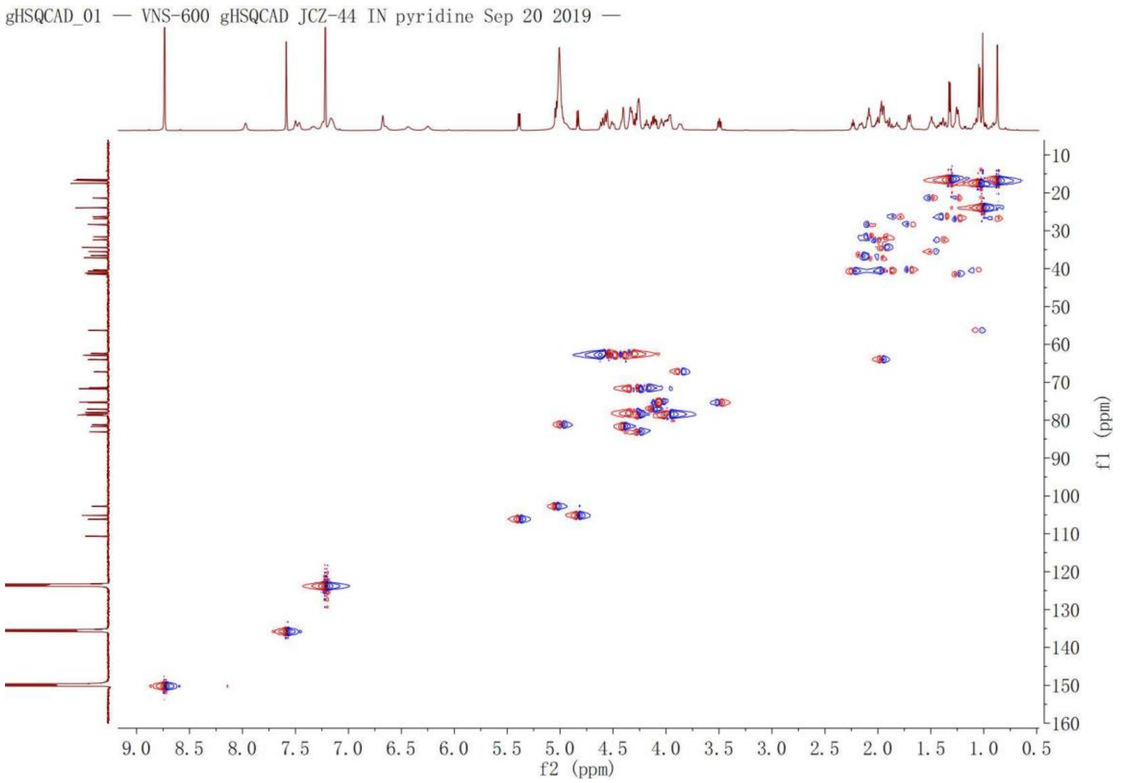

HSQC spectrum of 9.

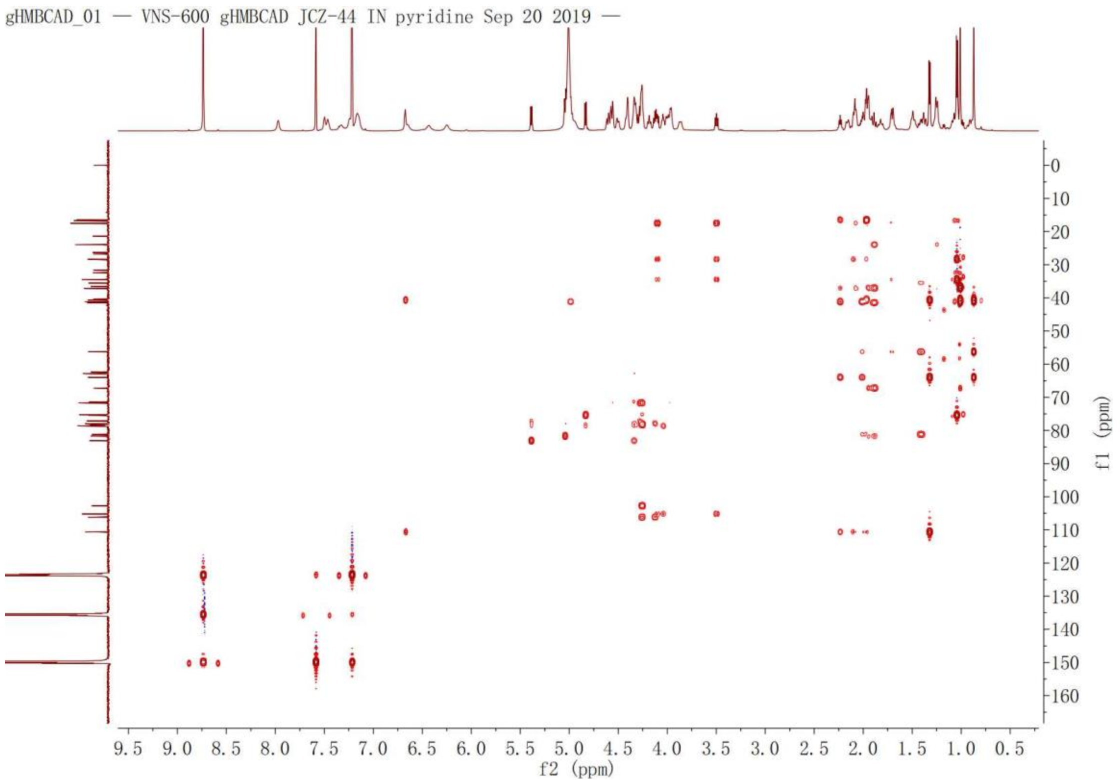

HMBC spectrum of 9.

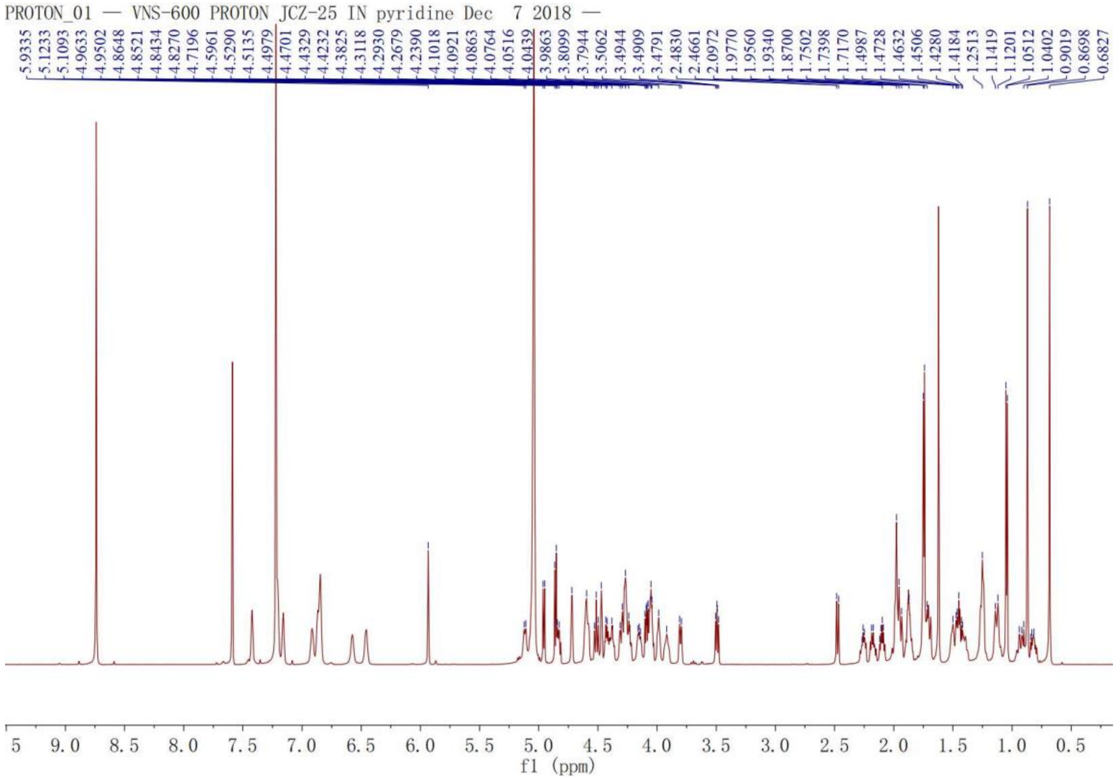

^{1}H NMR spectrum of 10.

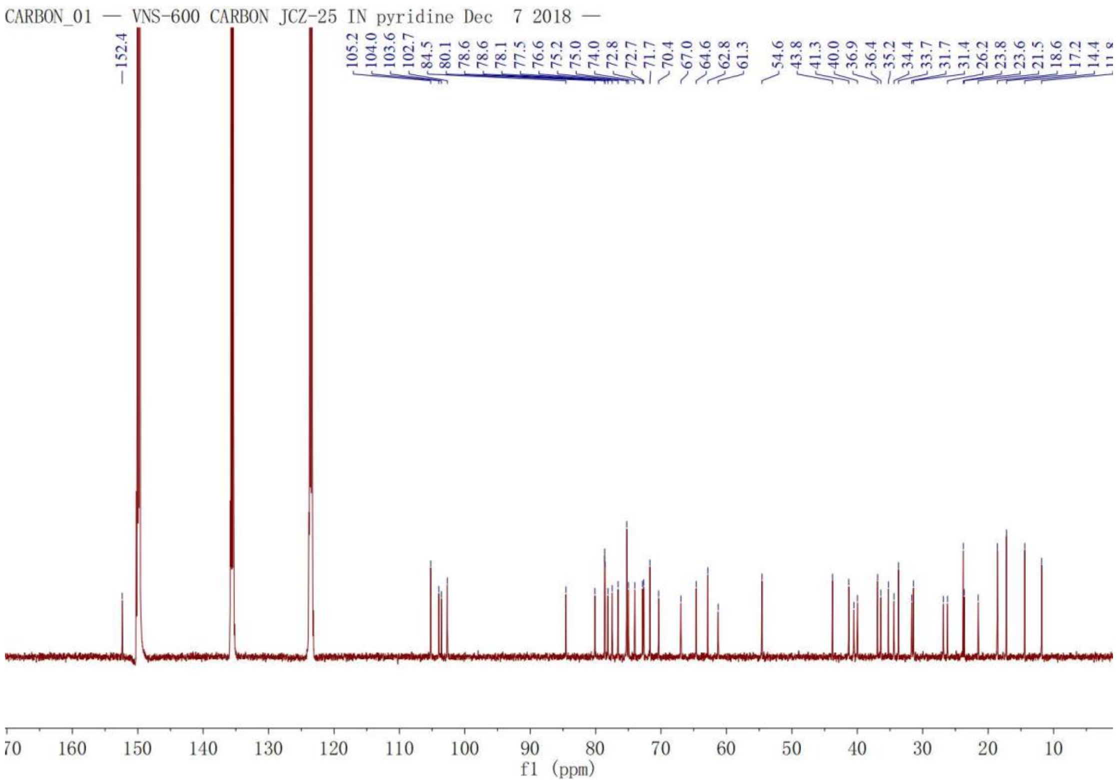

^{13}C NMR spectrum of 10.

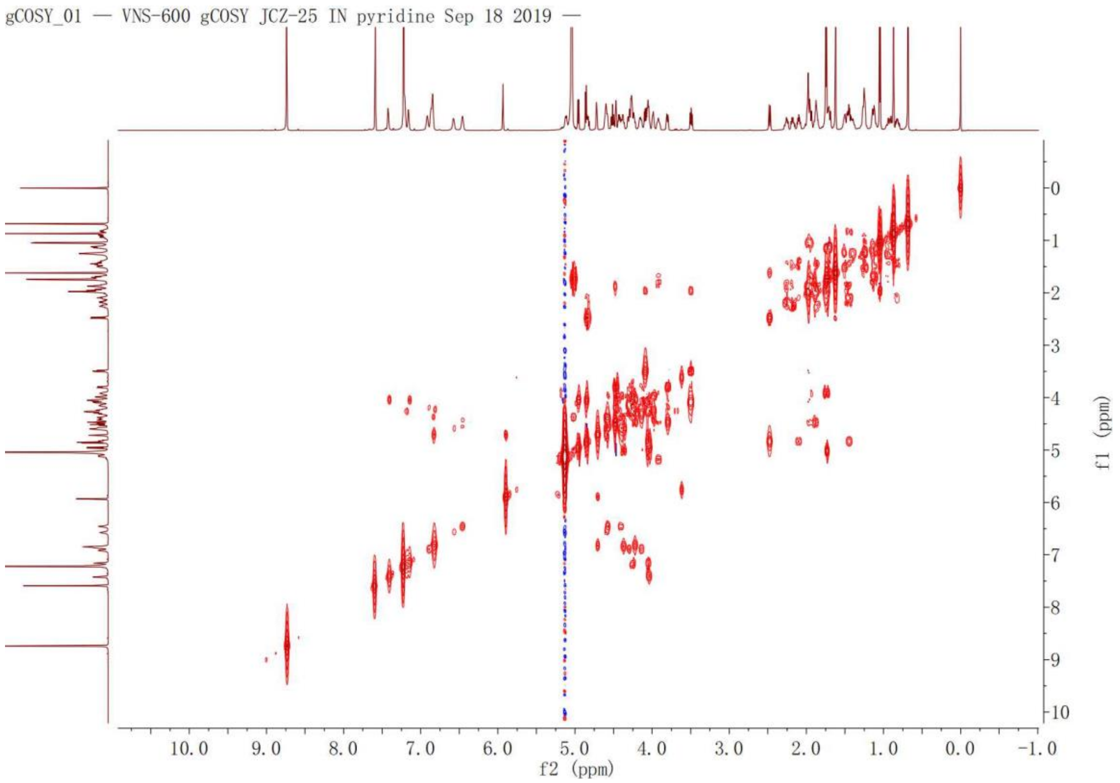

^{1}H-^{1}H COSY spectrum of 10.

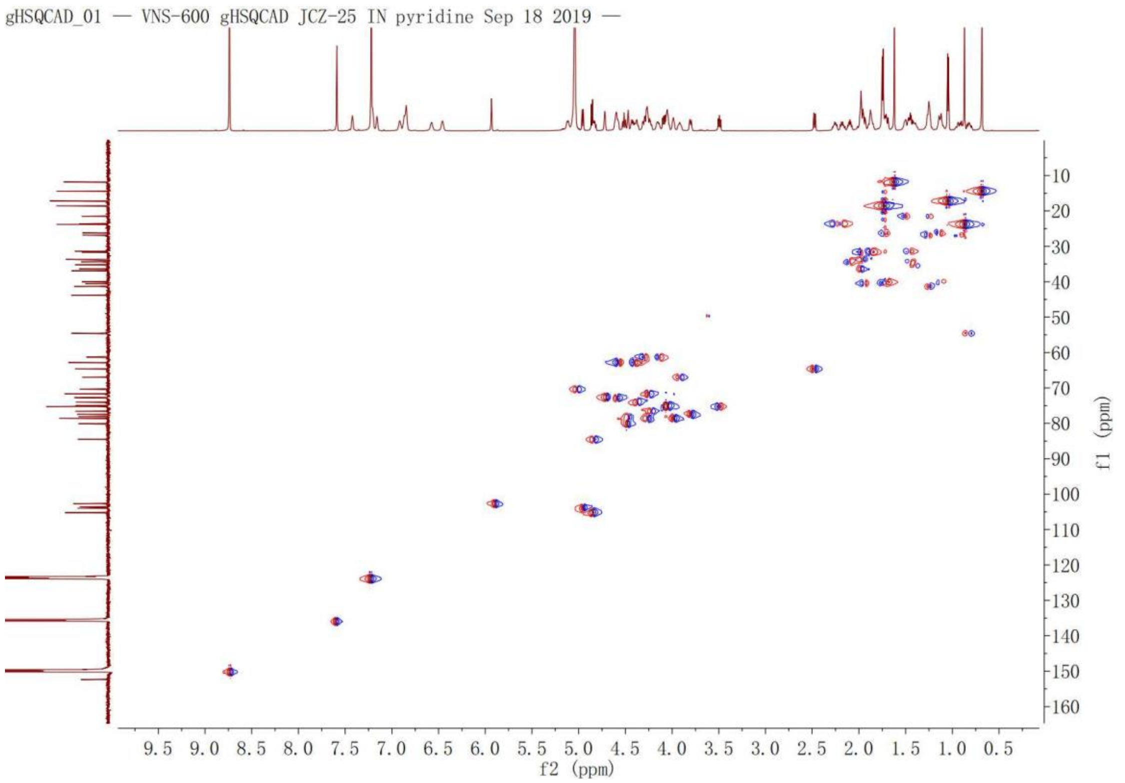

HSQC spectrum of 10.

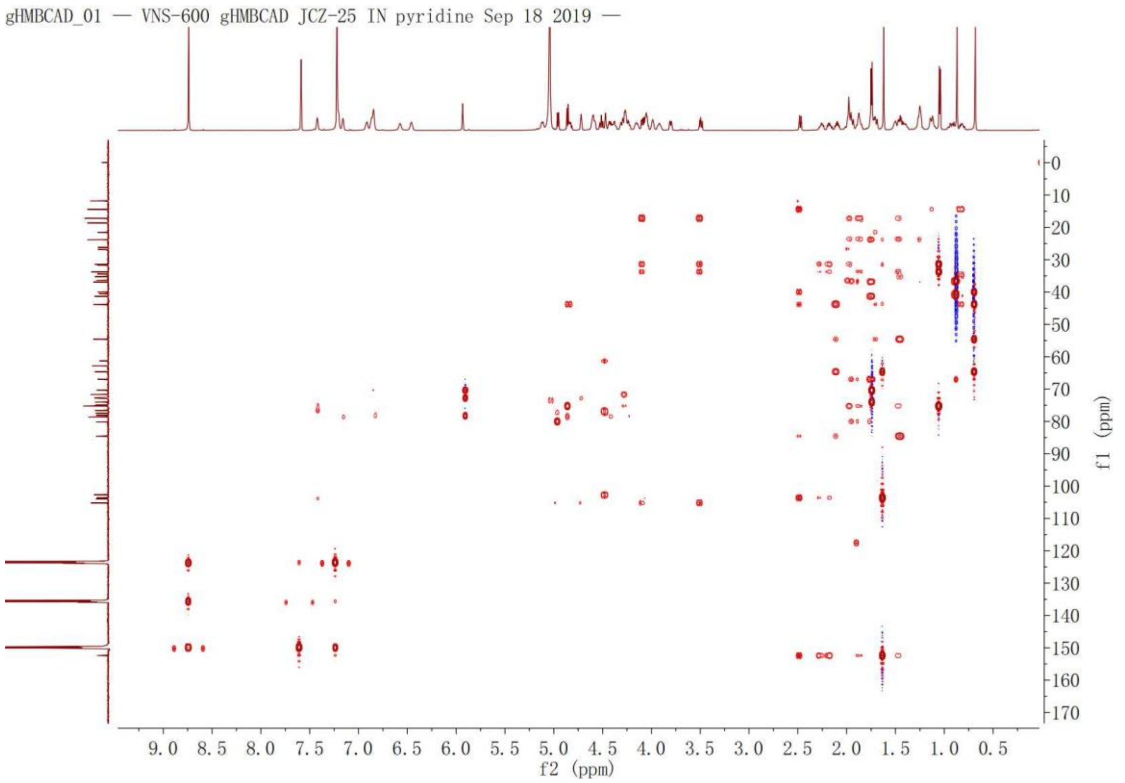

HMBC spectrum of 10.

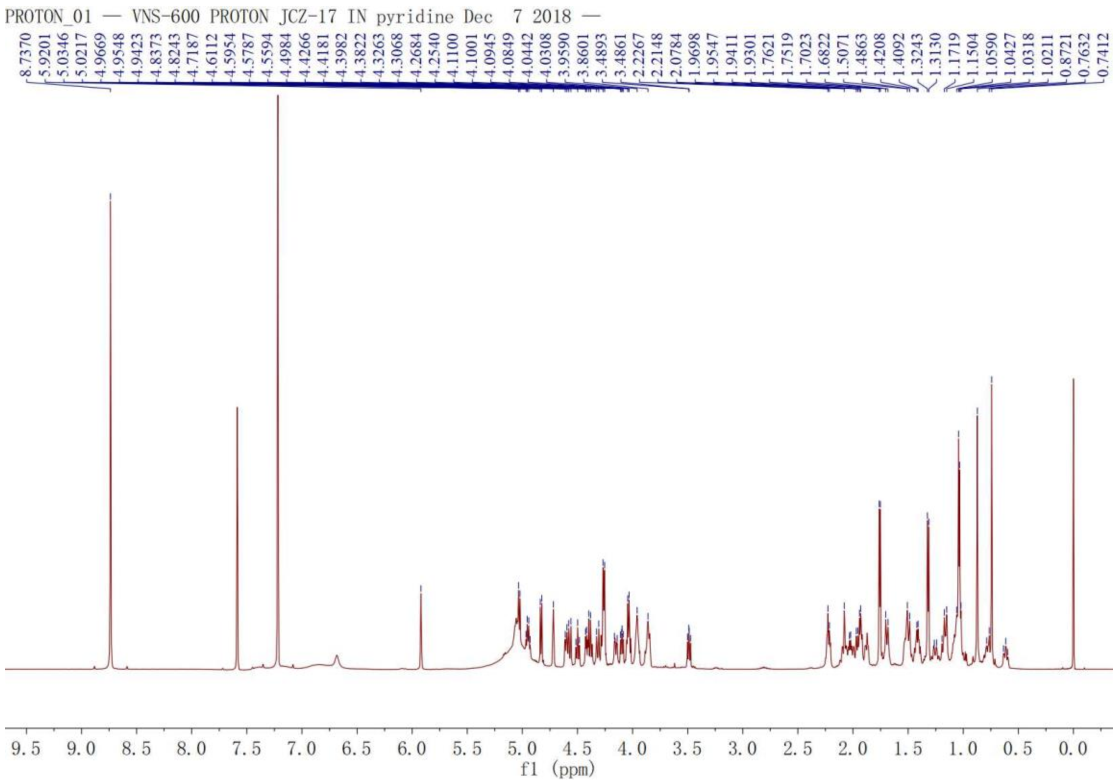

^{1}H NMR spectrum of 11.

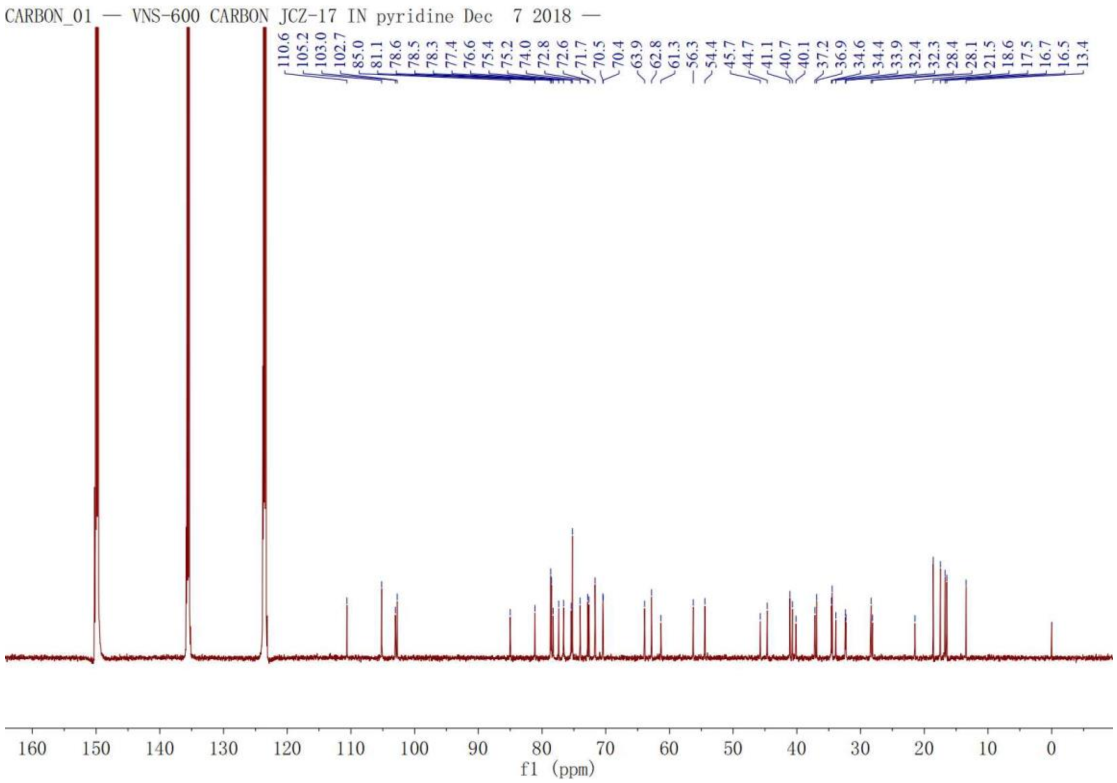

^{13}C NMR spectrum of 11.

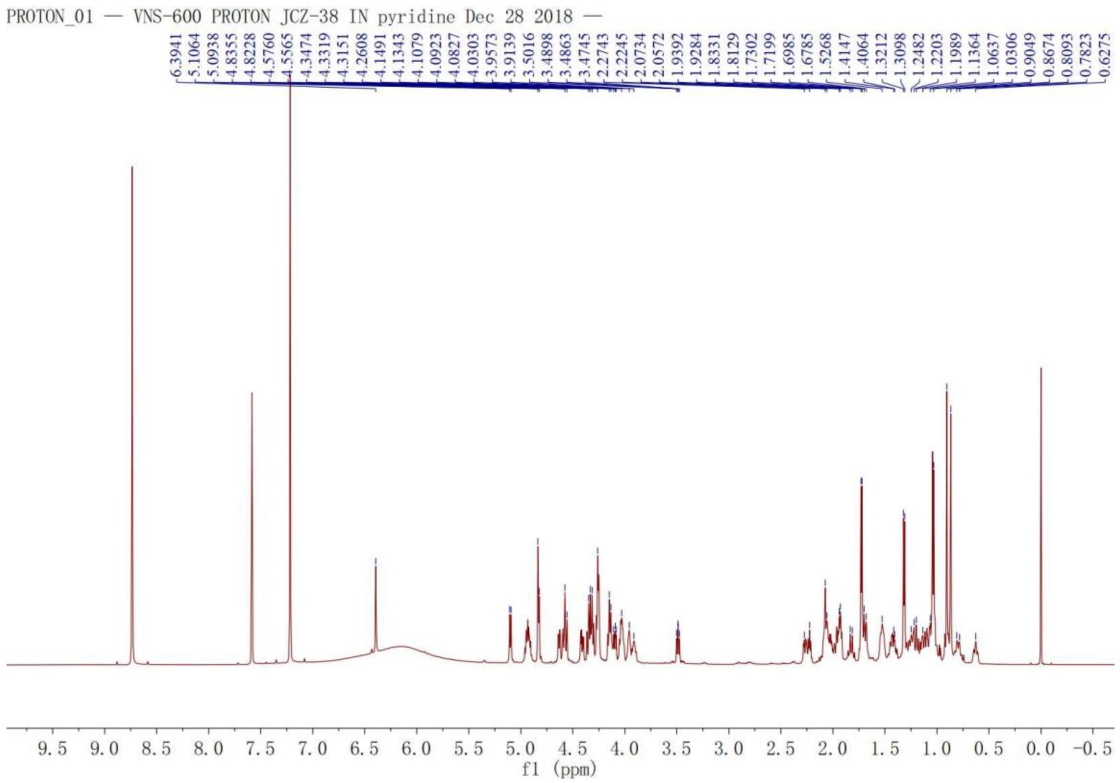

^{1}H NMR spectrum of 12.

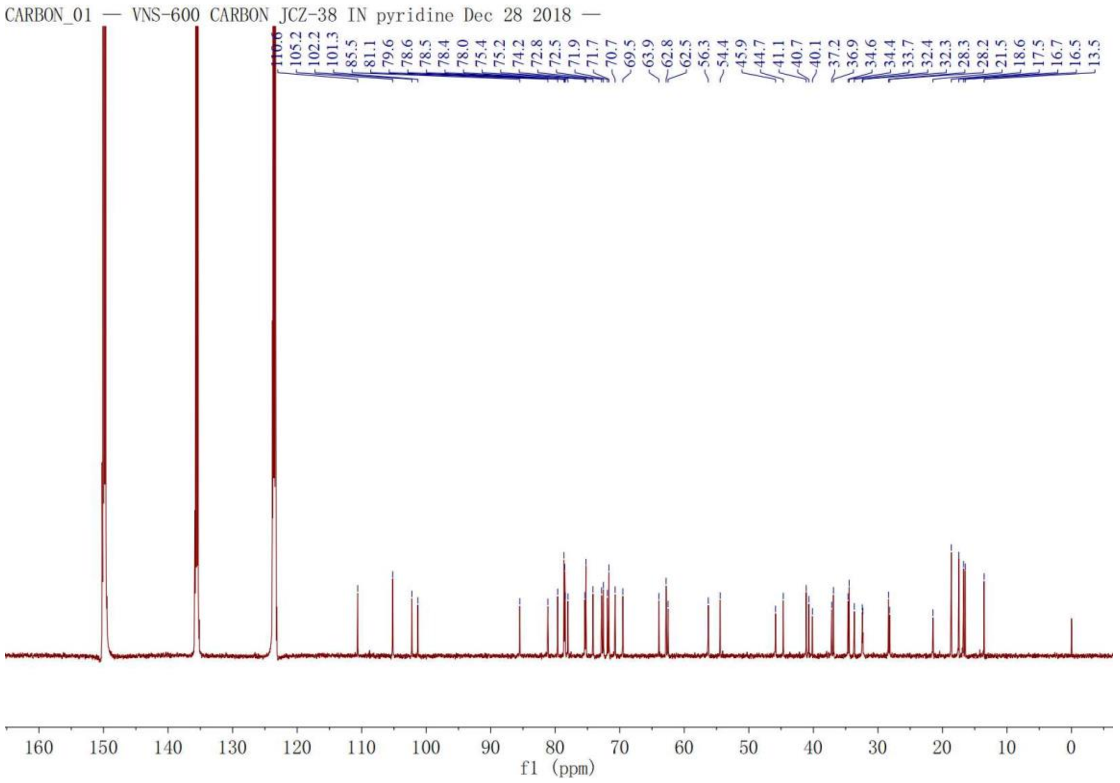

^{13}C NMR spectrum of 12.

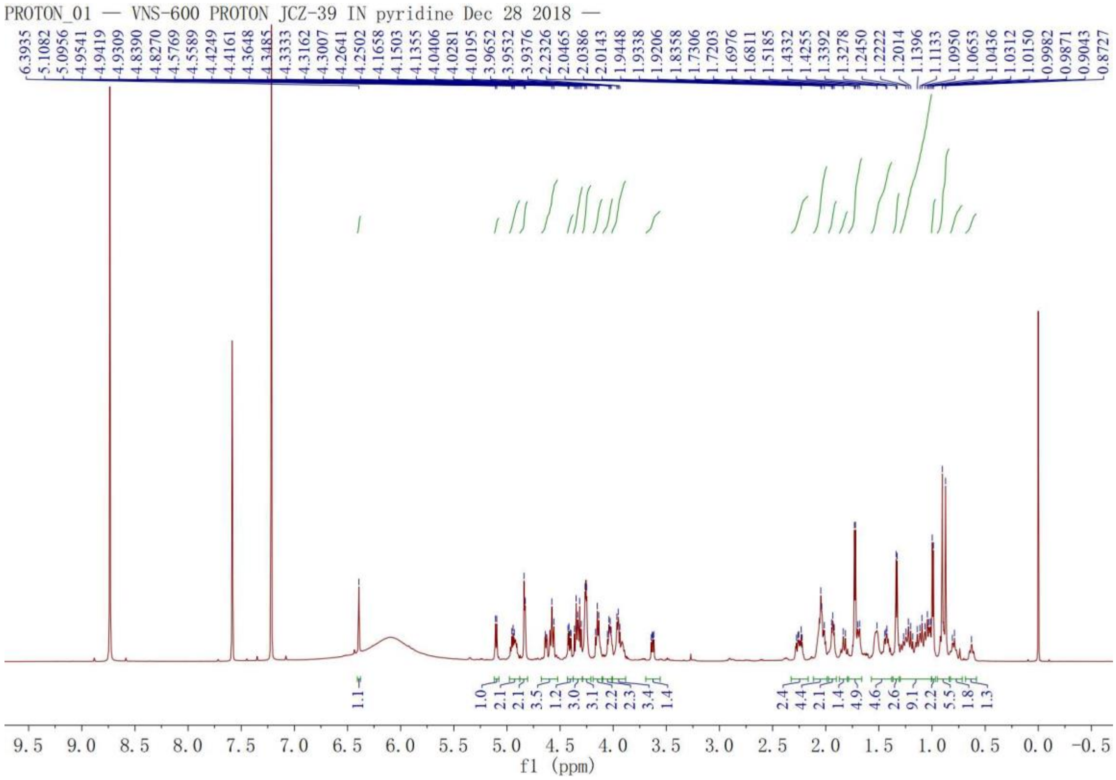

^{1}H NMR spectrum of 13.

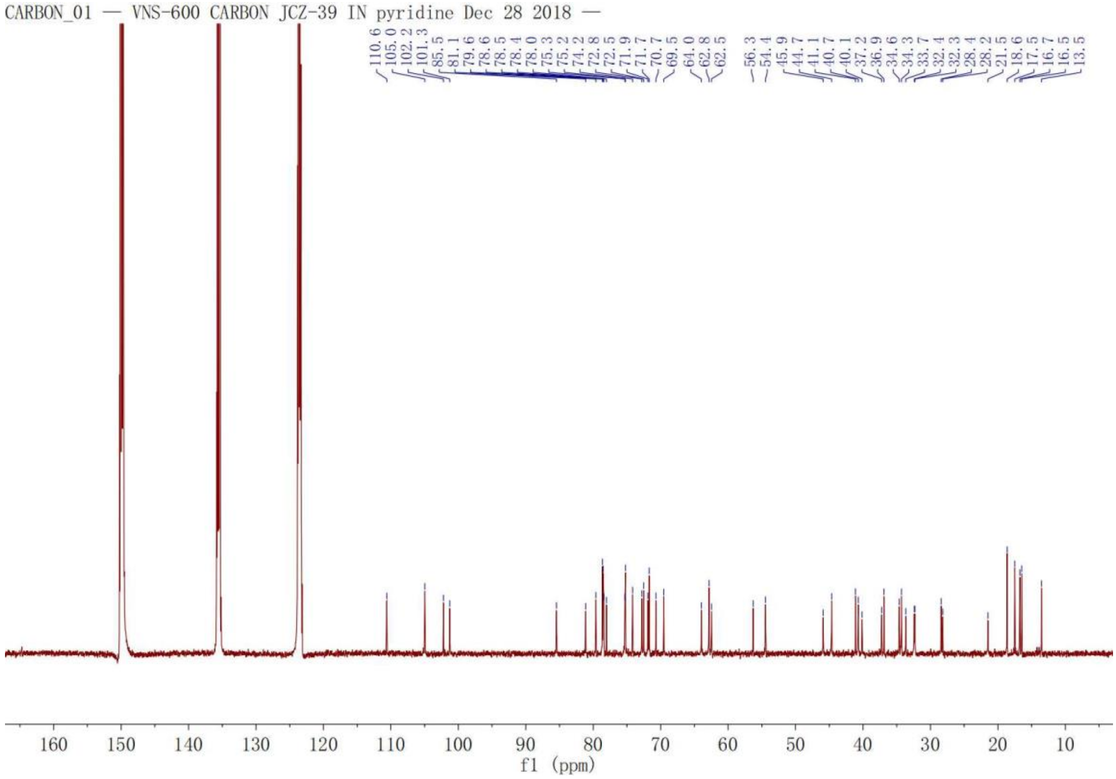

^{13}C NMR spectrum of 13.

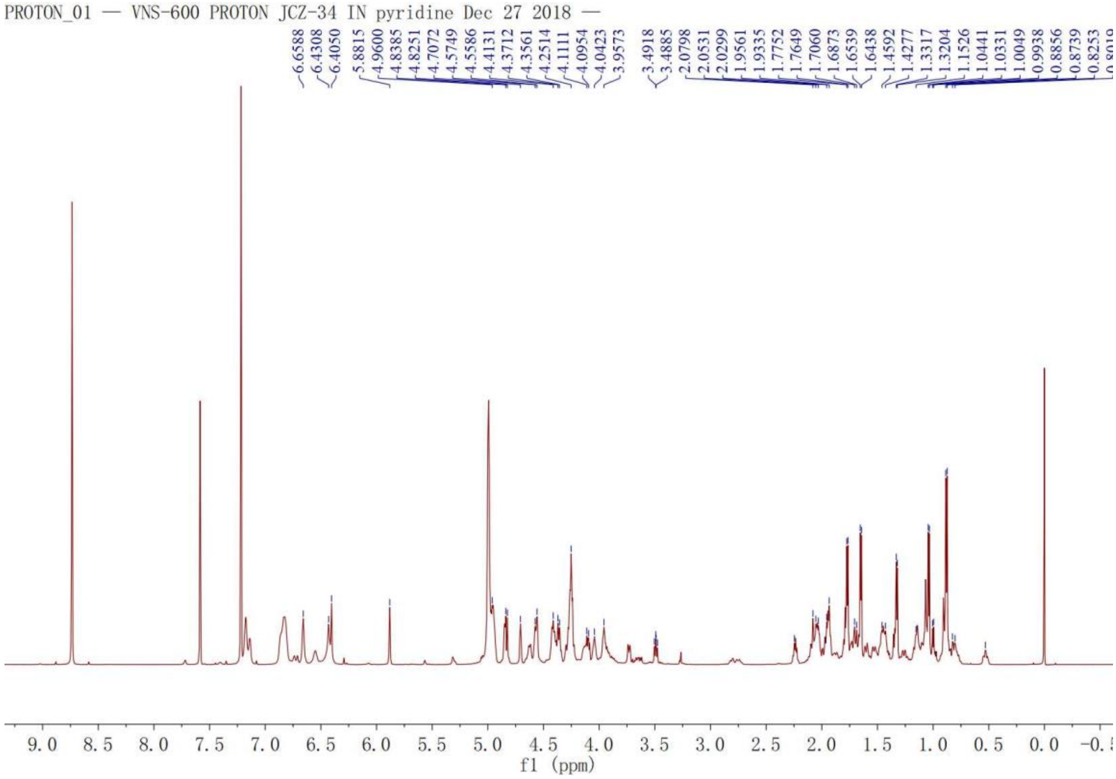

^{1}H NMR spectrum of **14**

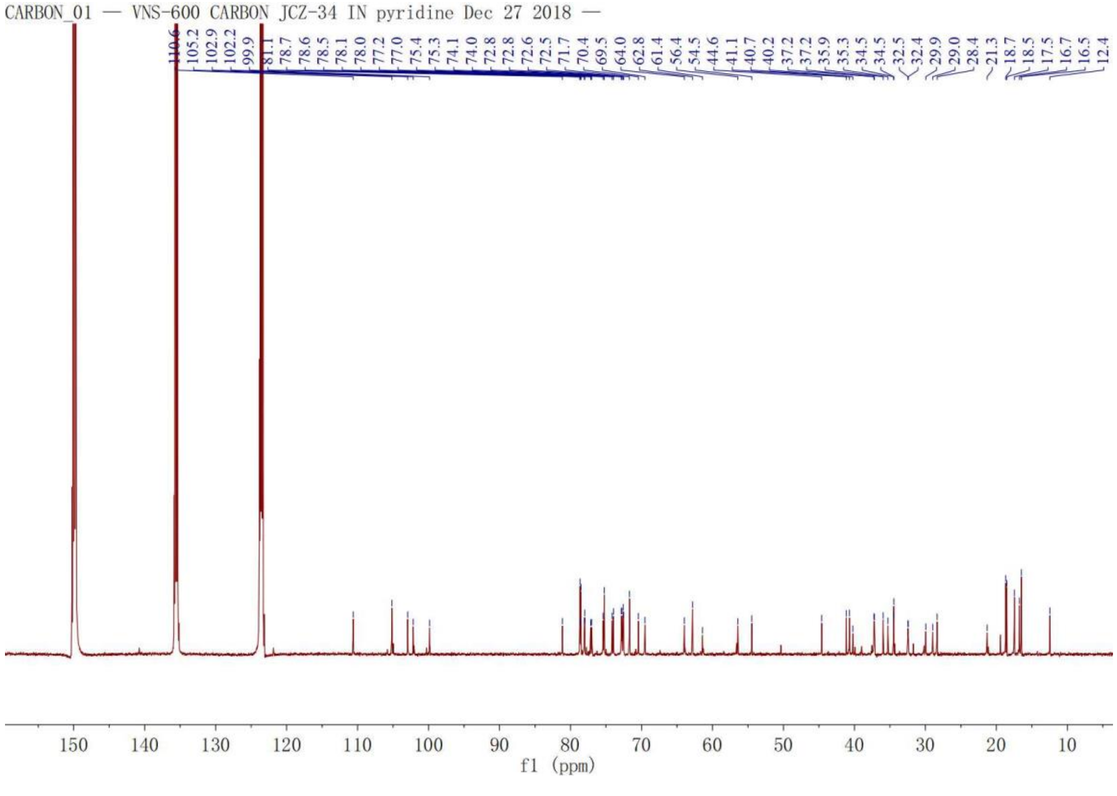

^{13}C NMR spectrum of 14.

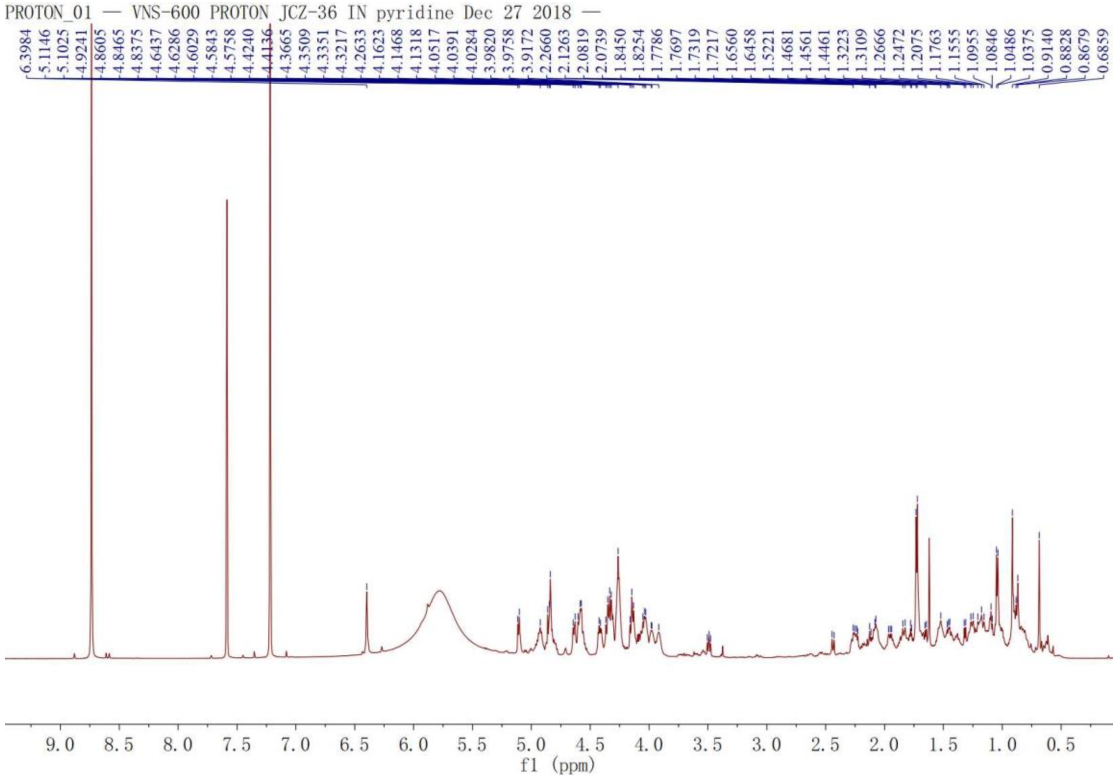

^{1}H NMR spectrum of 15.

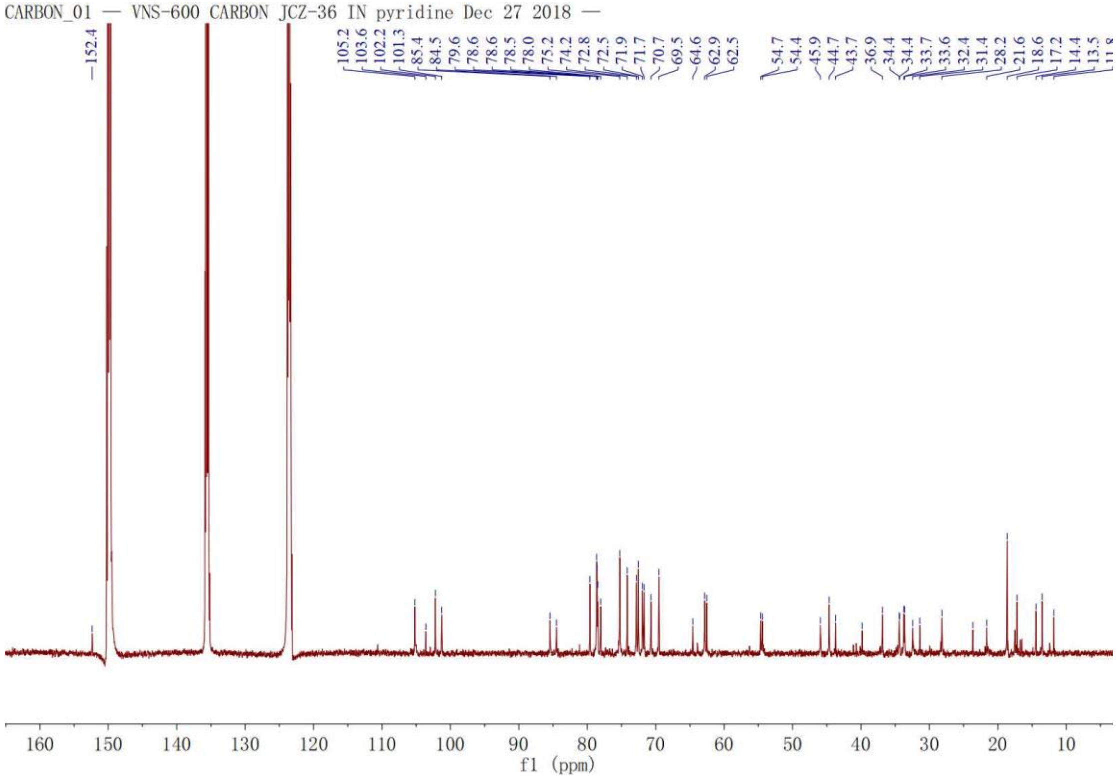

^{13}C NMR spectrum of 15.

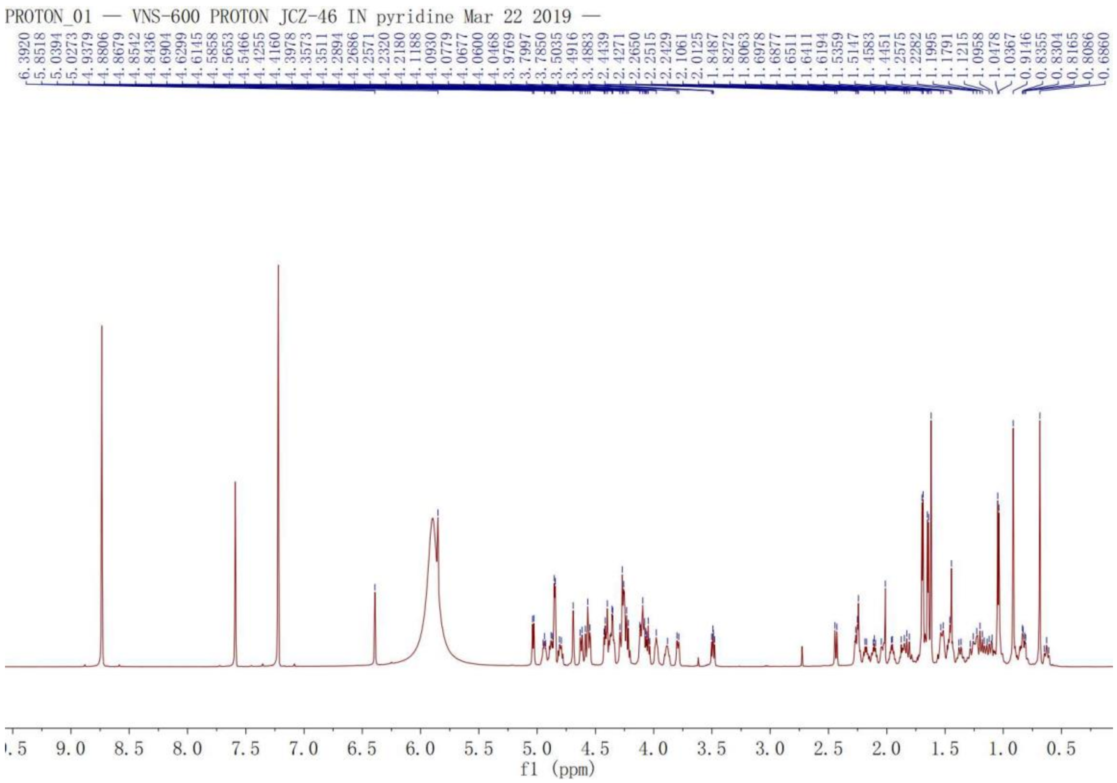

^{1}H NMR spectrum of 16.

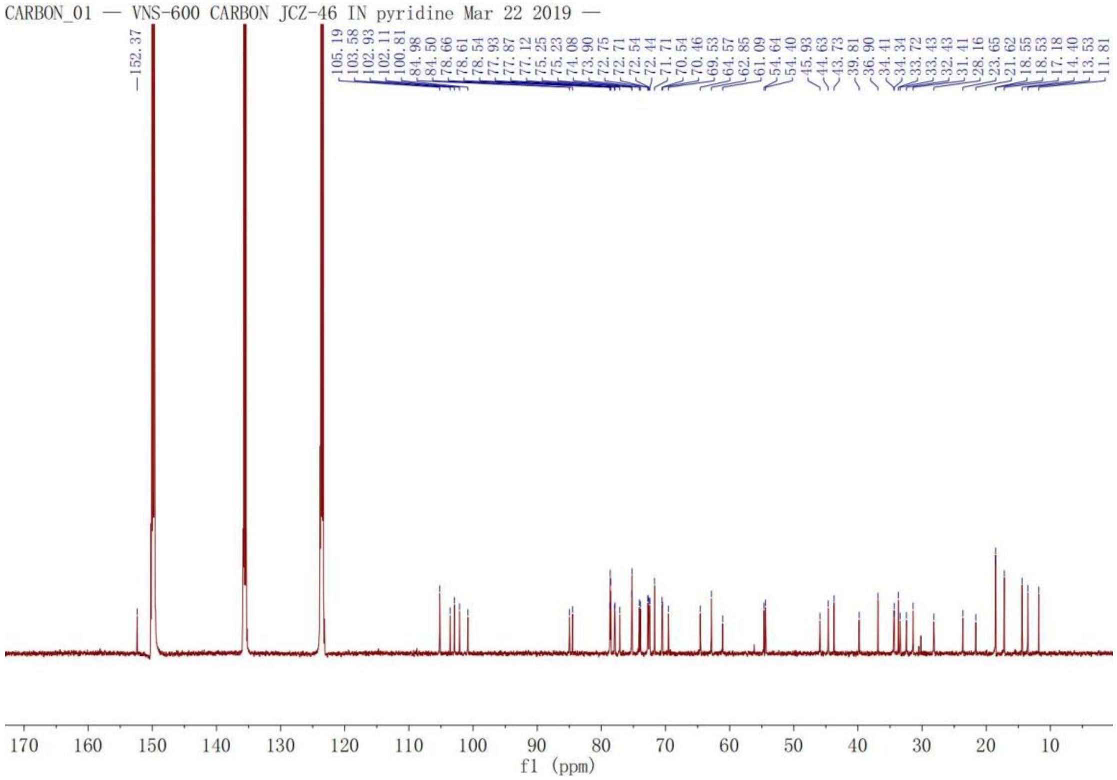

^{13}C NMR spectrum of 16.

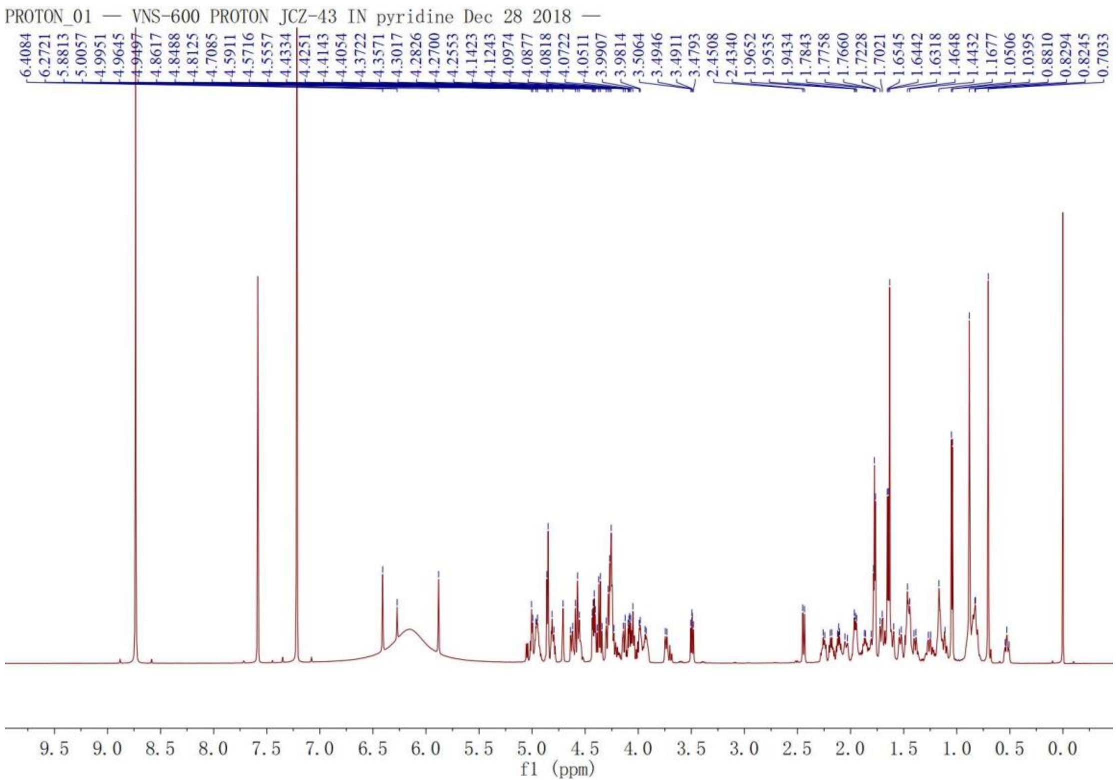

^{1}H NMR spectrum of 17.

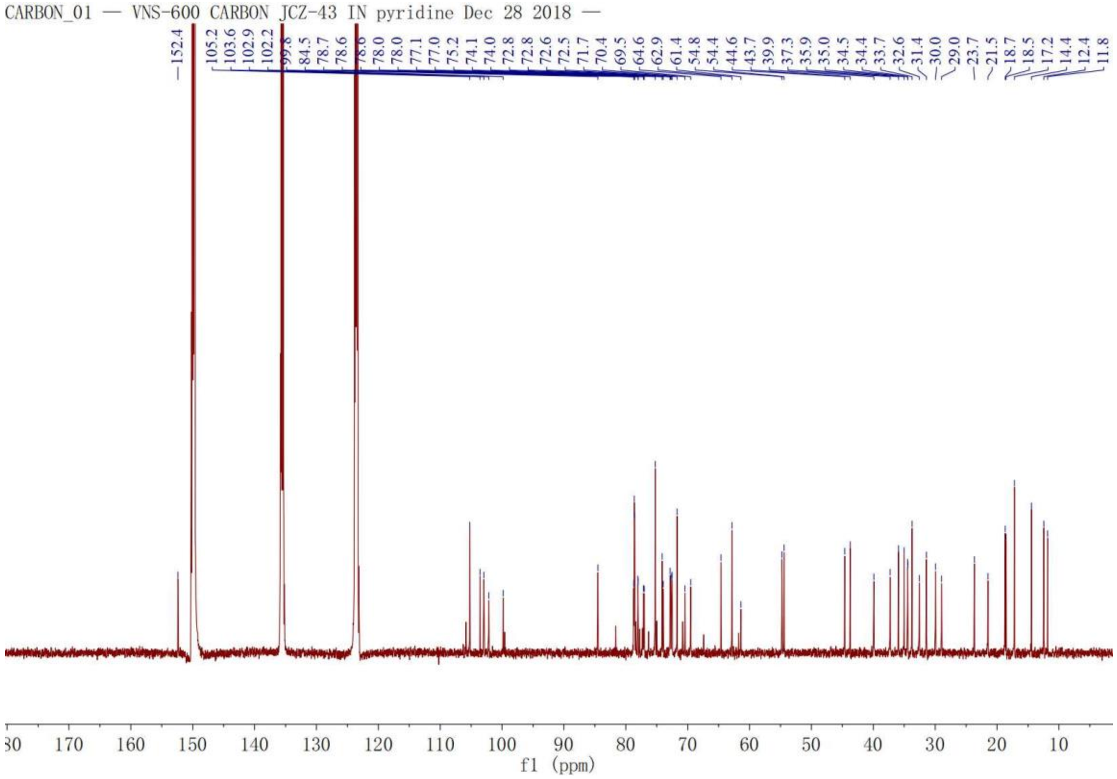

^{13}C NMR spectrum of 17.

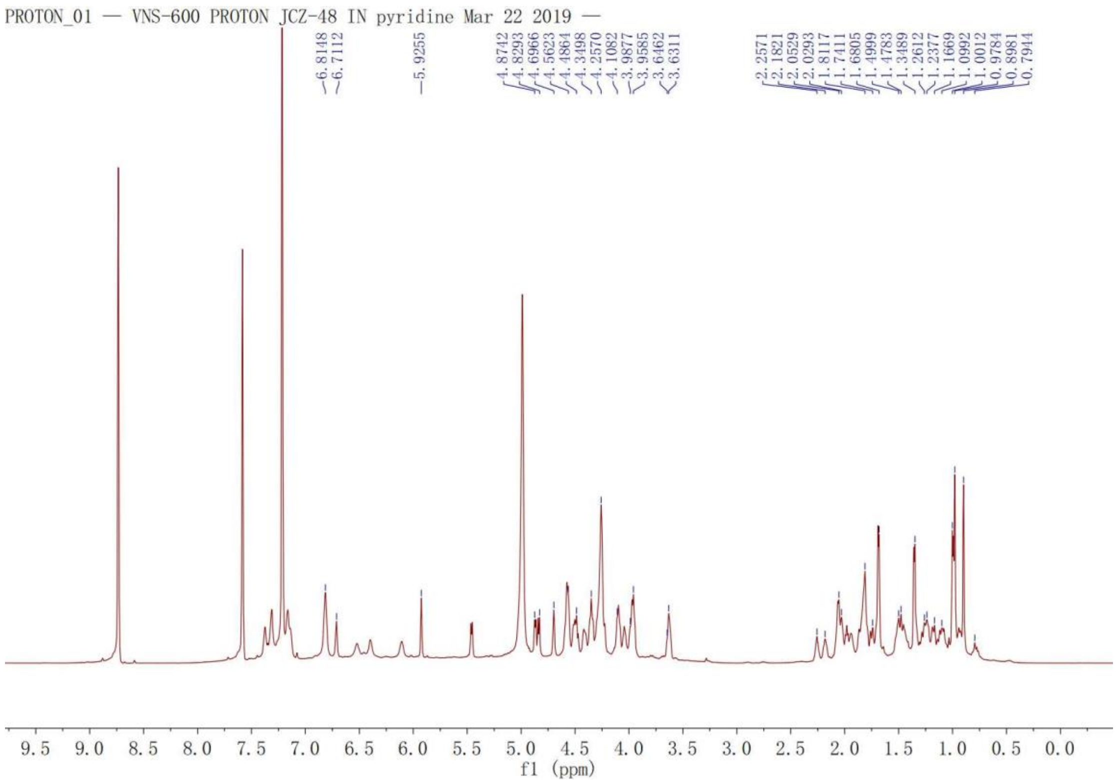

^{1}H NMR spectrum of 18.

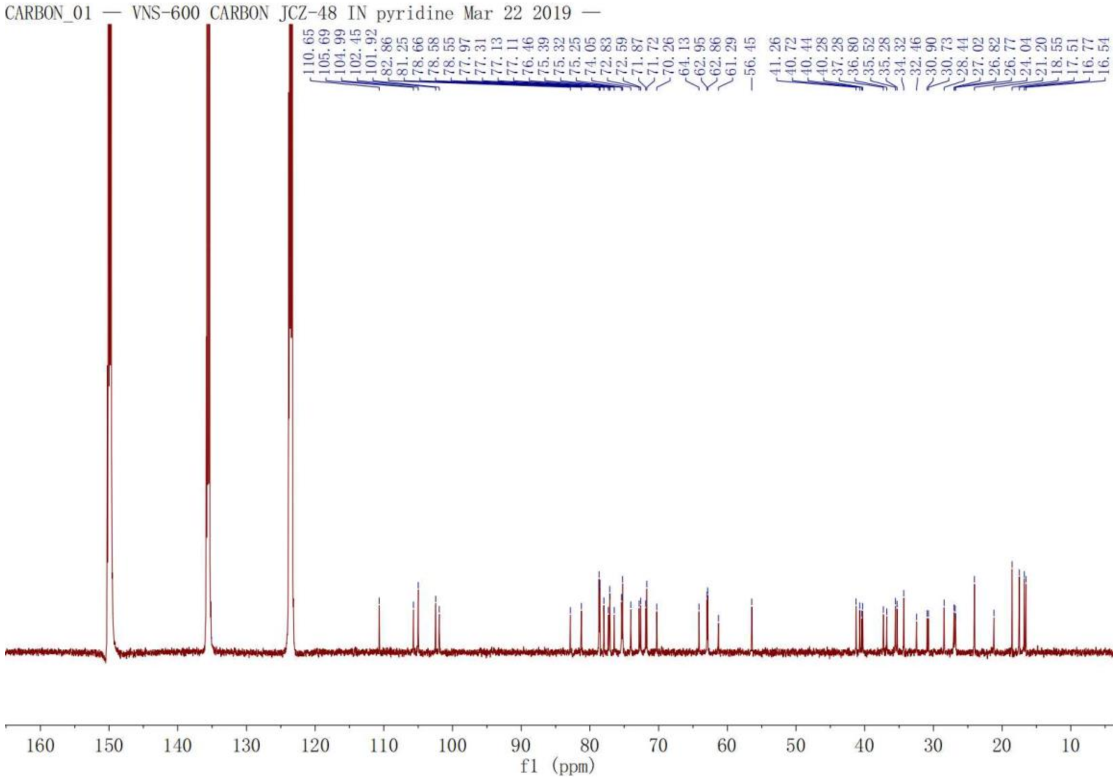

^{13}C NMR spectrum of 18.

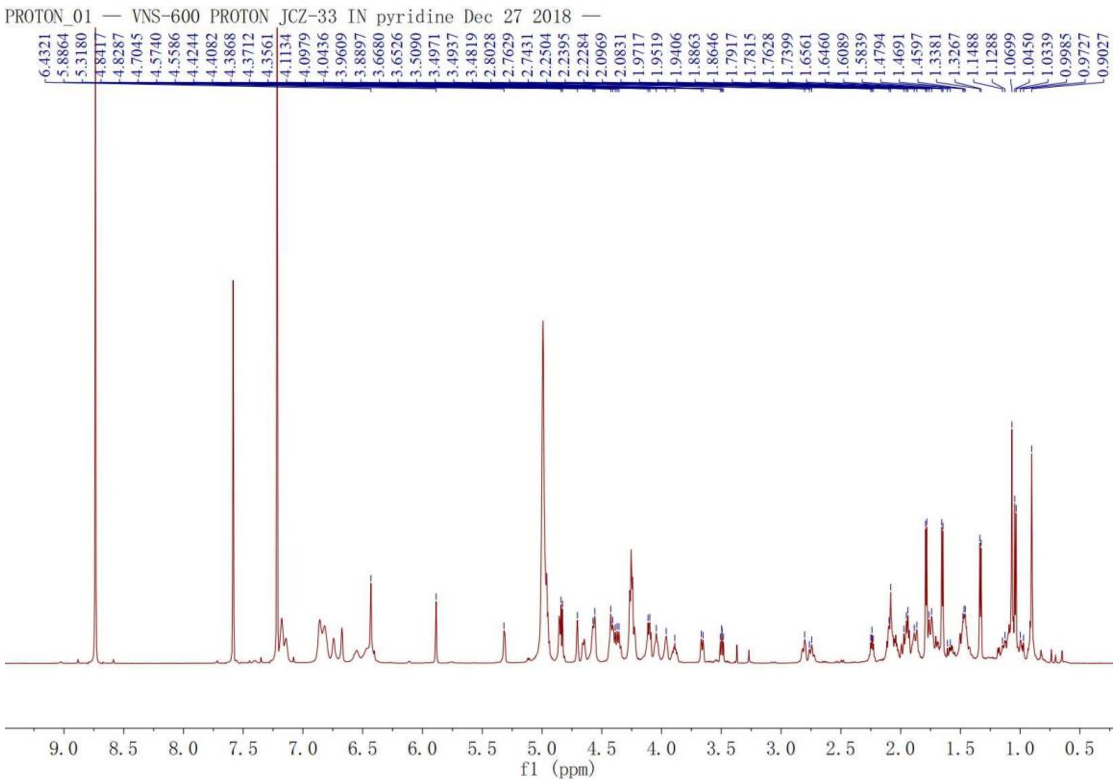

^{1}H NMR spectrum of 19.

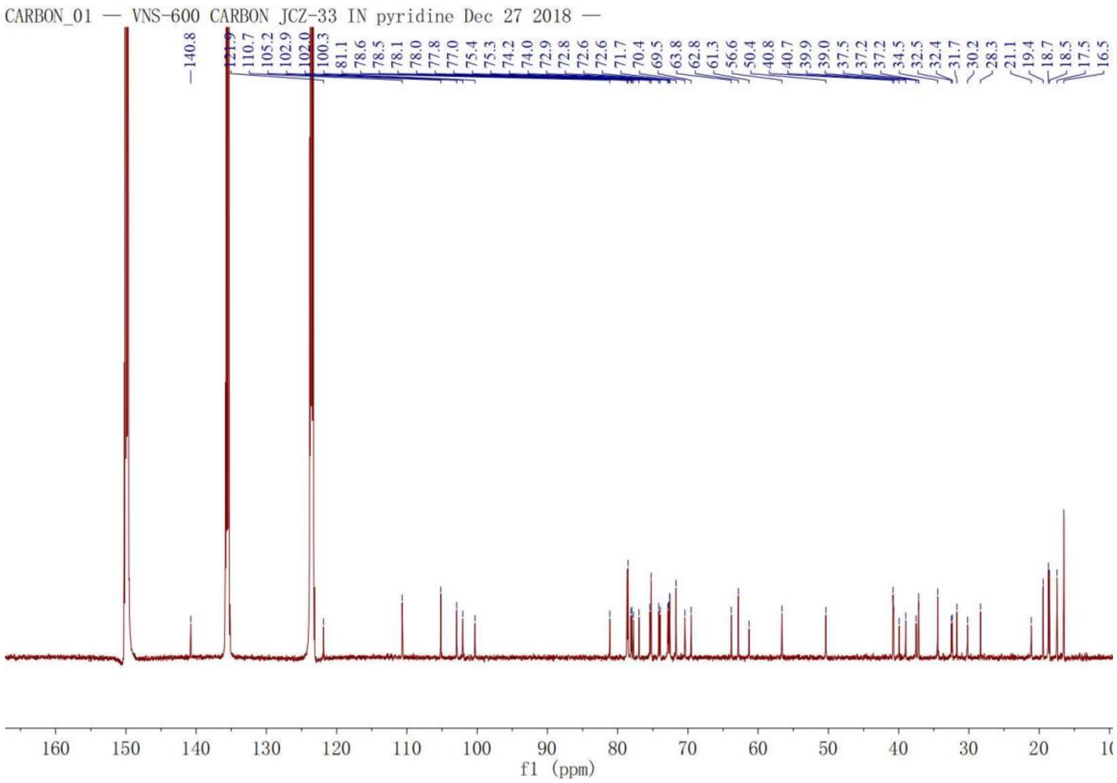

^{13}C NMR spectrum of 19.

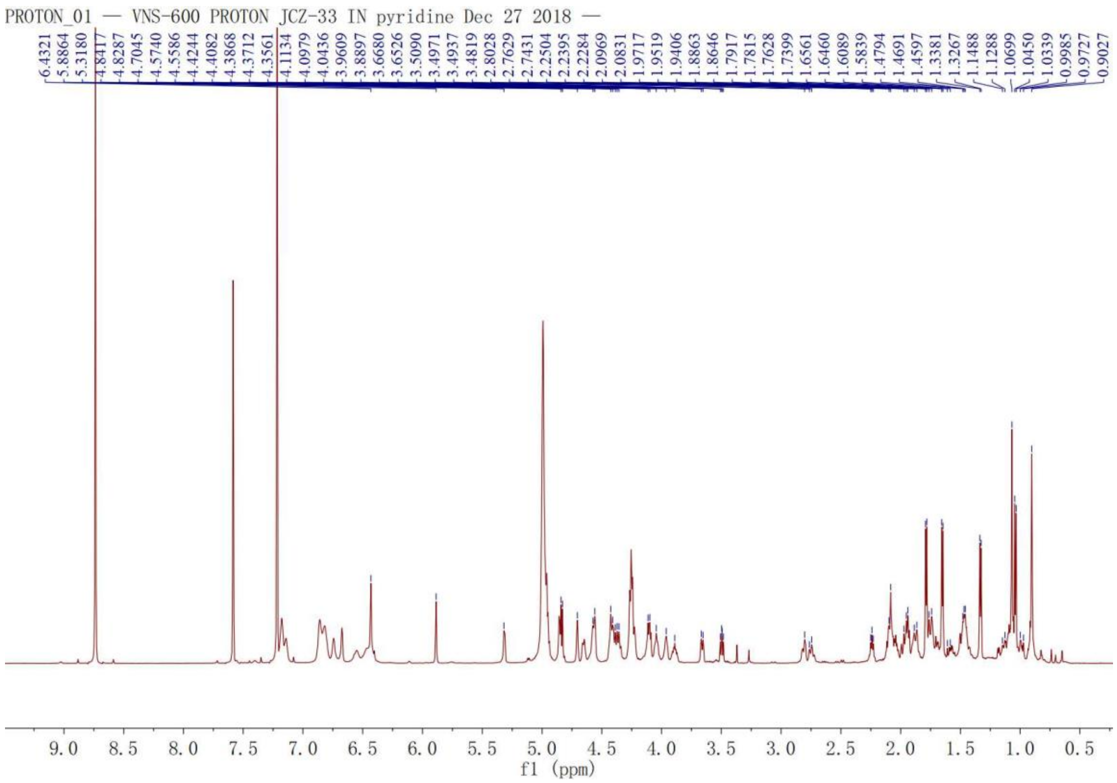

^{1}H NMR spectrum of 20.

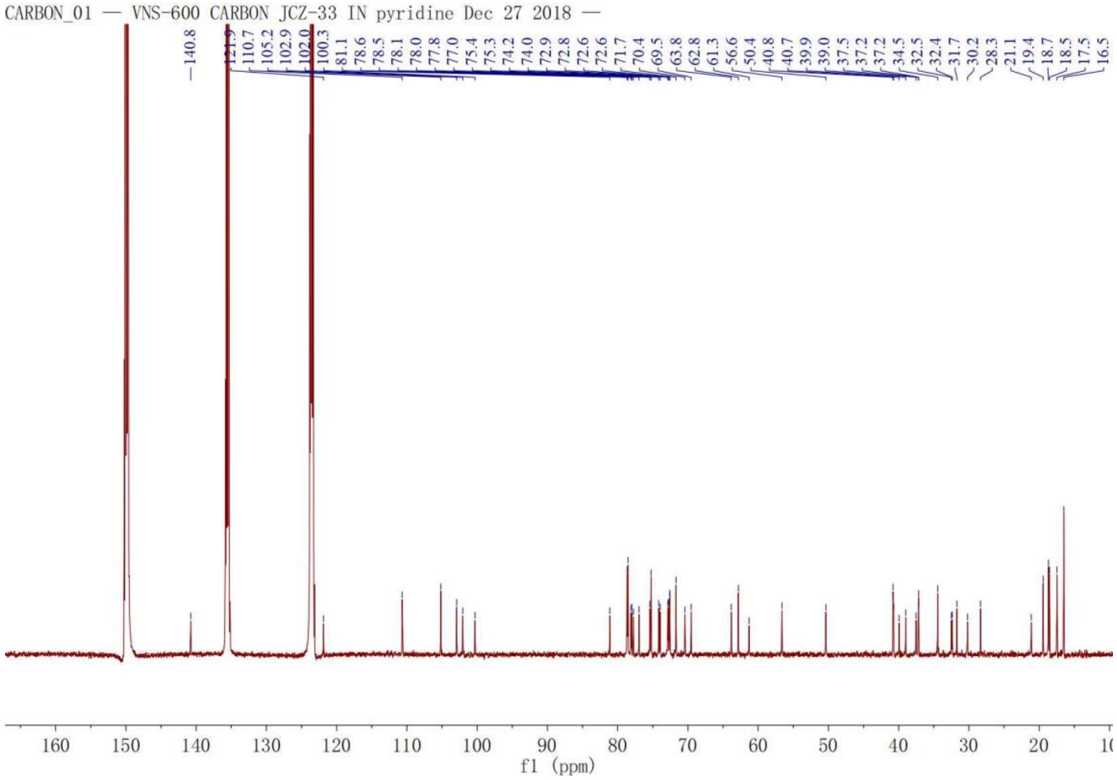

^{13}C NMR spectrum of 20.

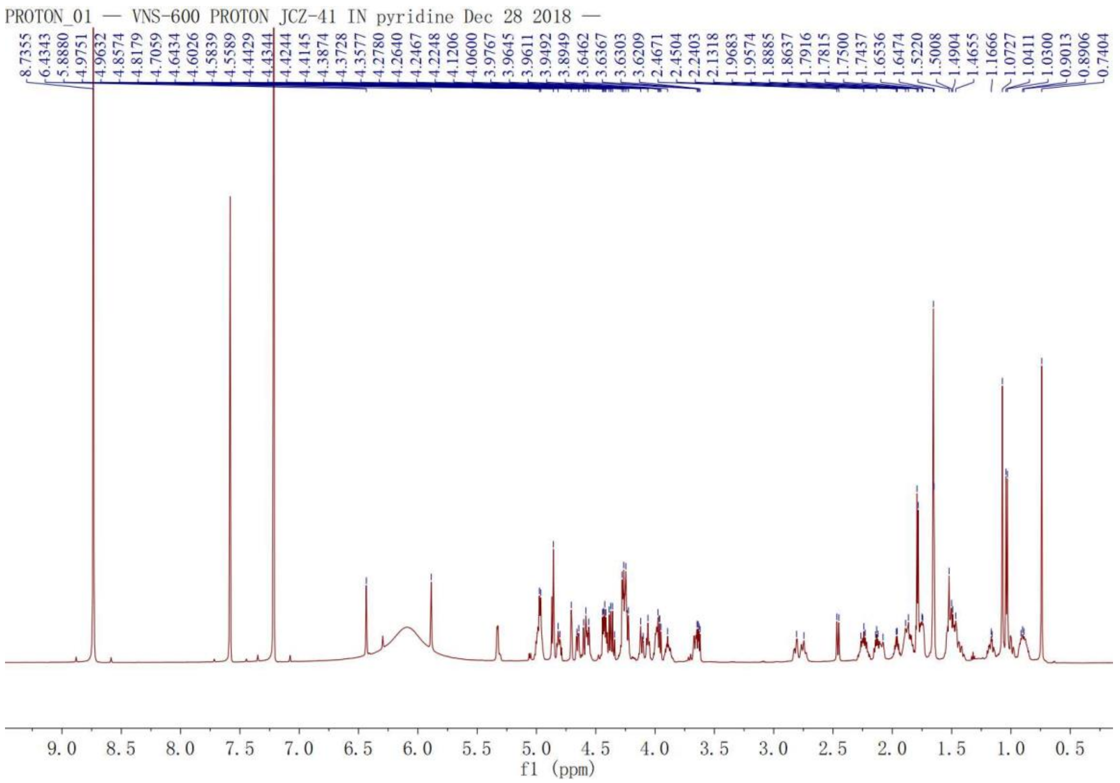

^{1}H NMR spectrum of 21.

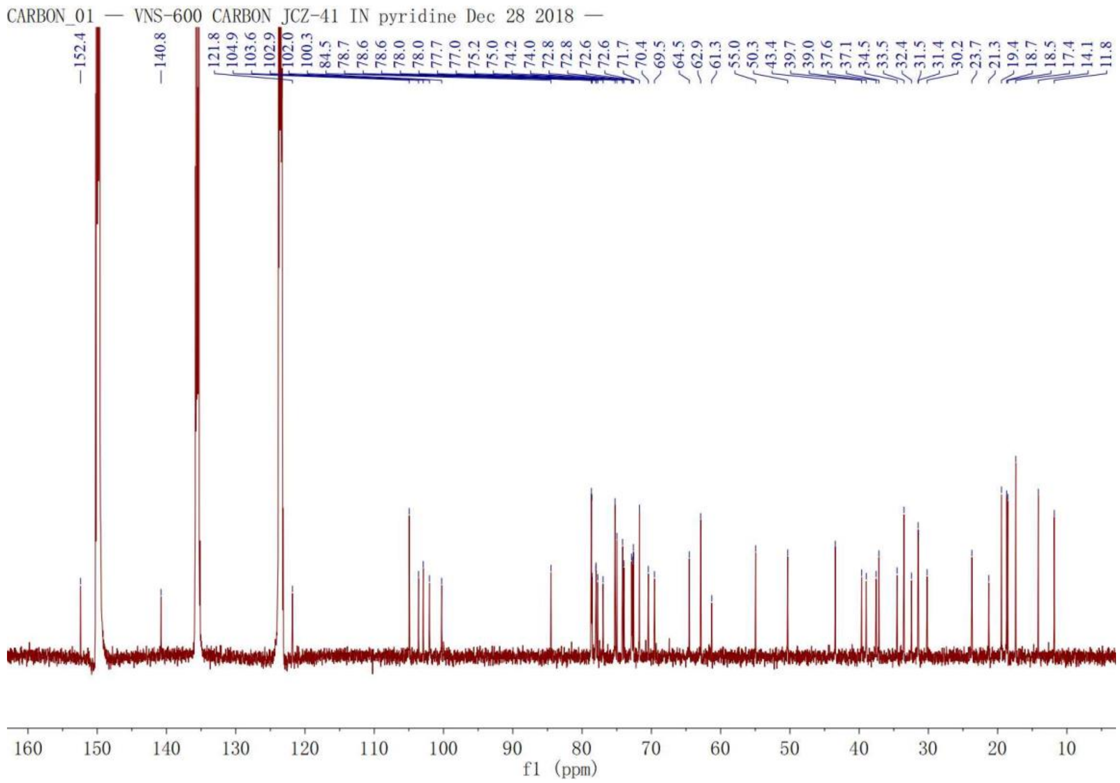

^{13}C NMR spectrum of 21.

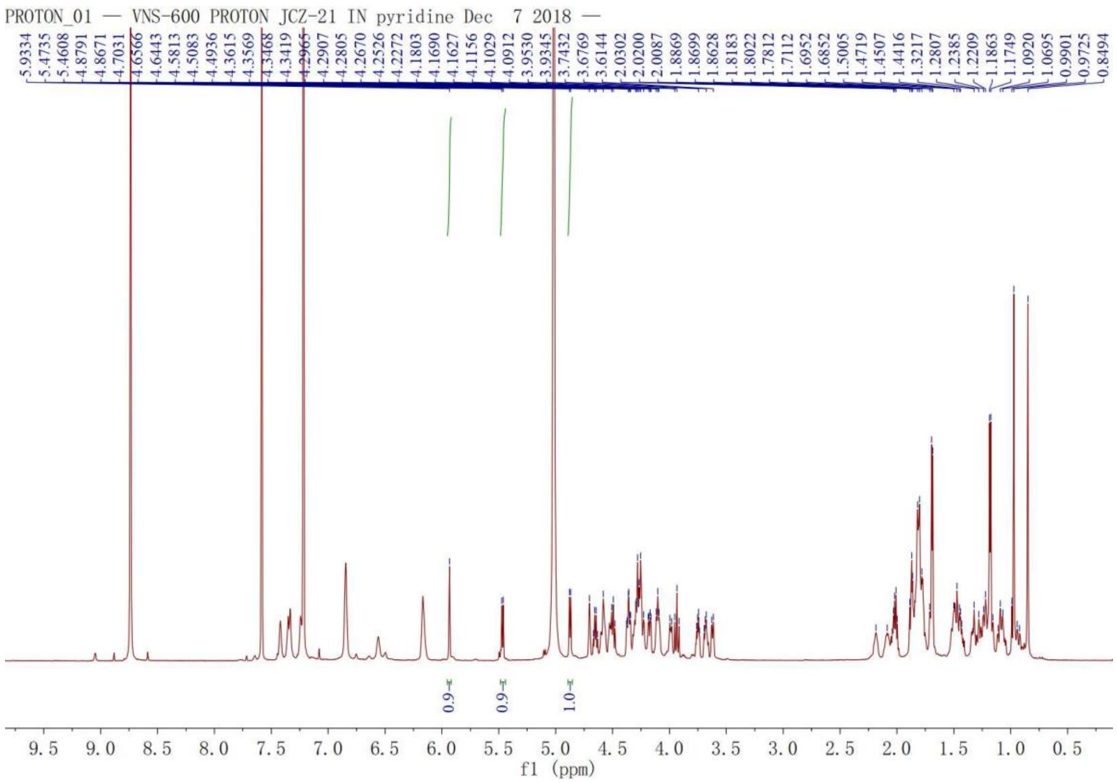

^{1}H NMR spectrum of 22.

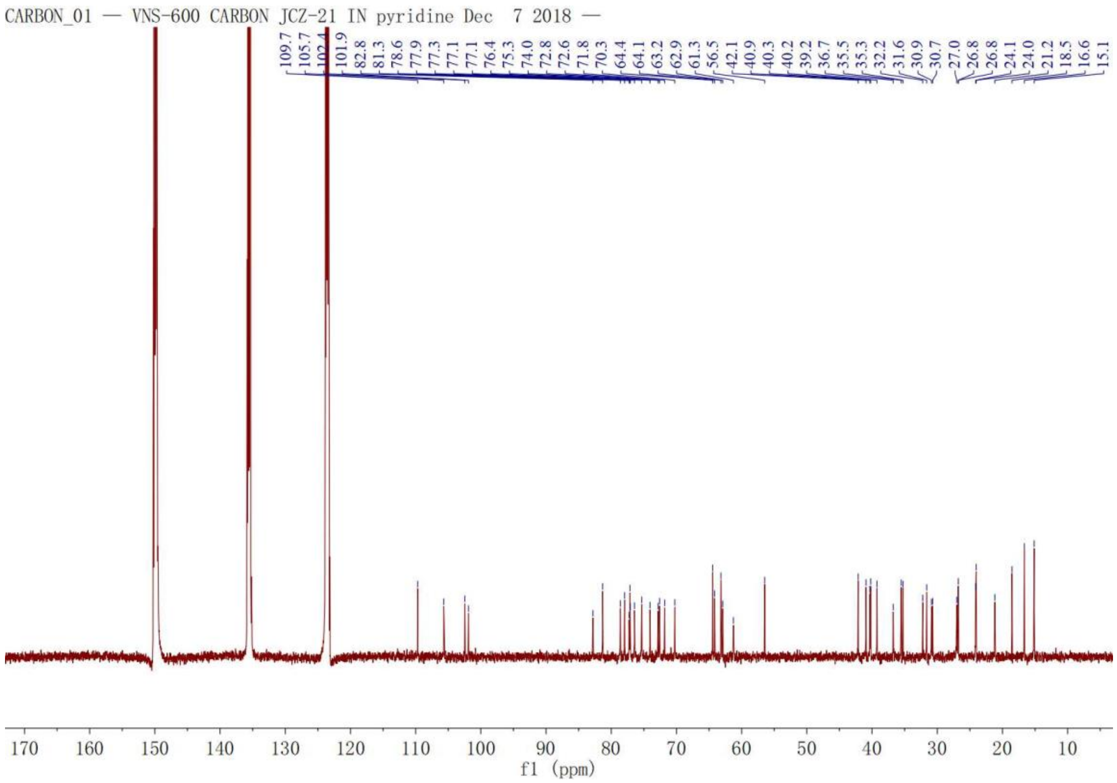

^{13}C NMR spectrum of 22.

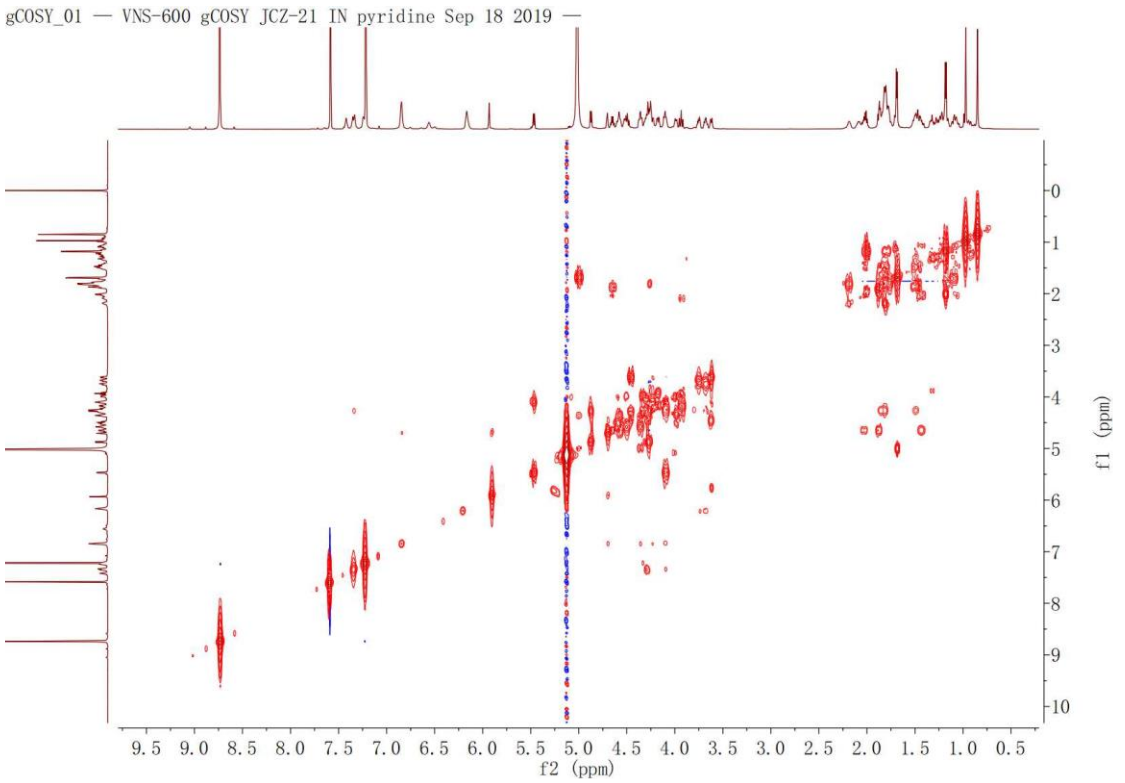

^{1}H-^{1}H COSY spectrum of 22.

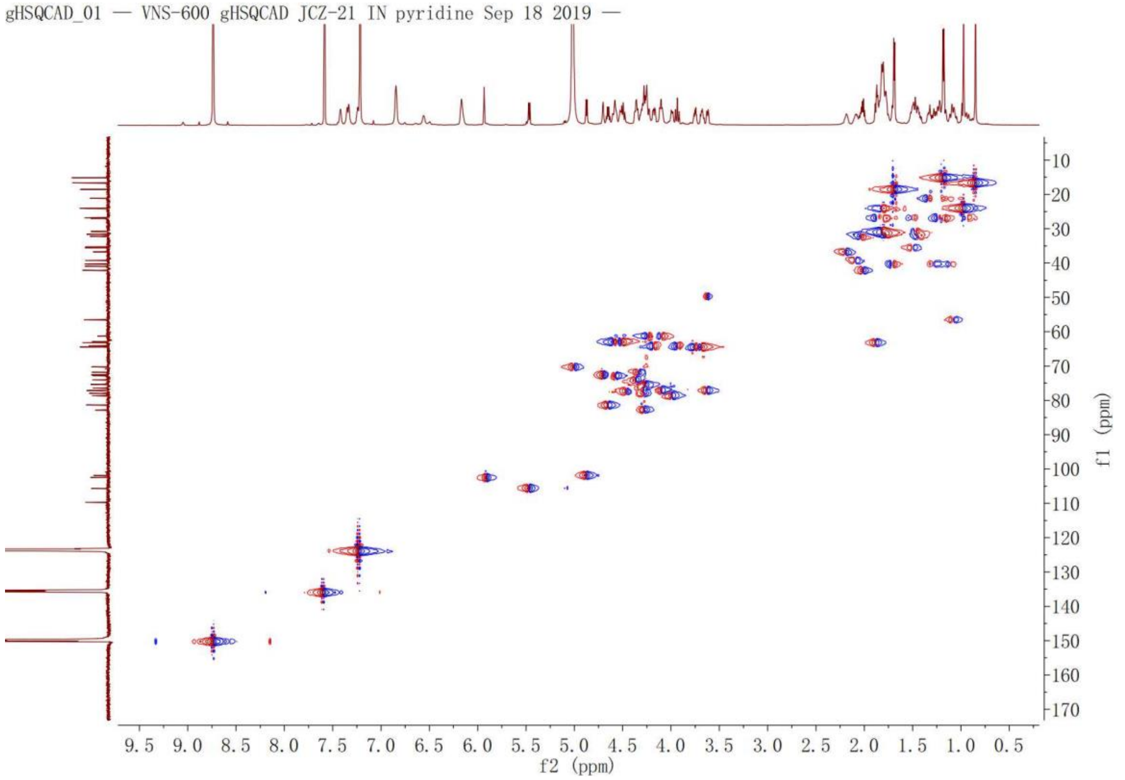

HSQC spectrum of 22.

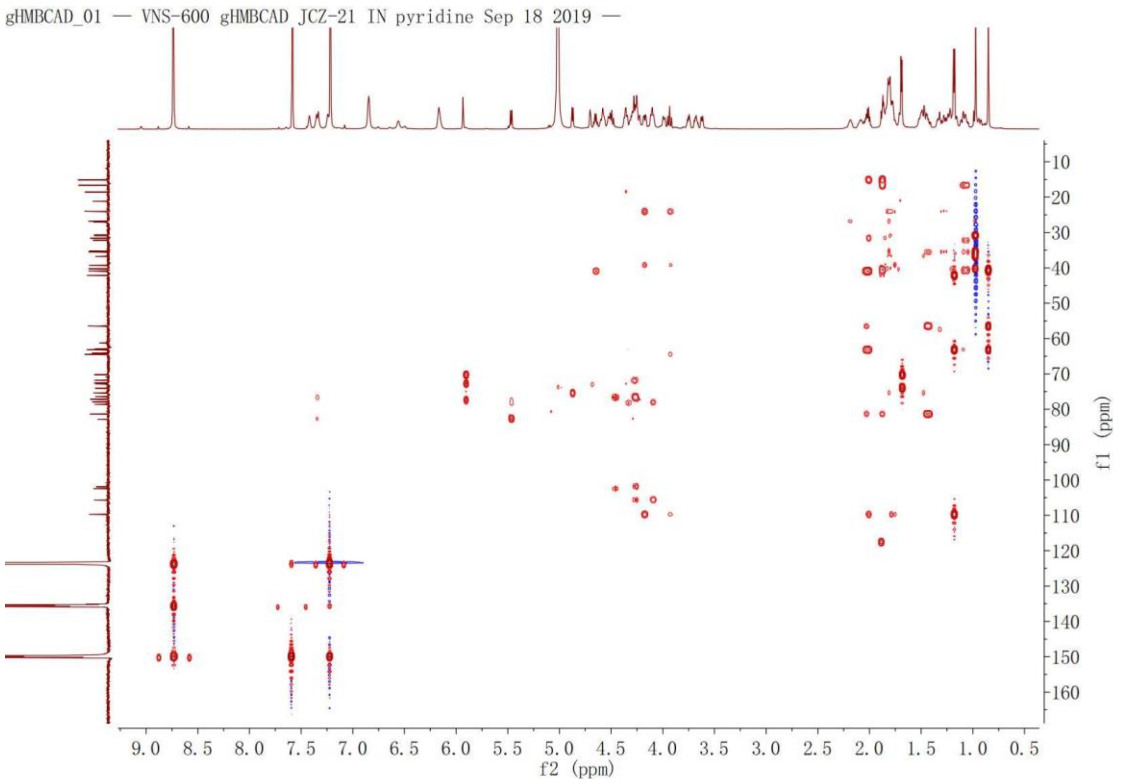

HMBC spectrum of 22.

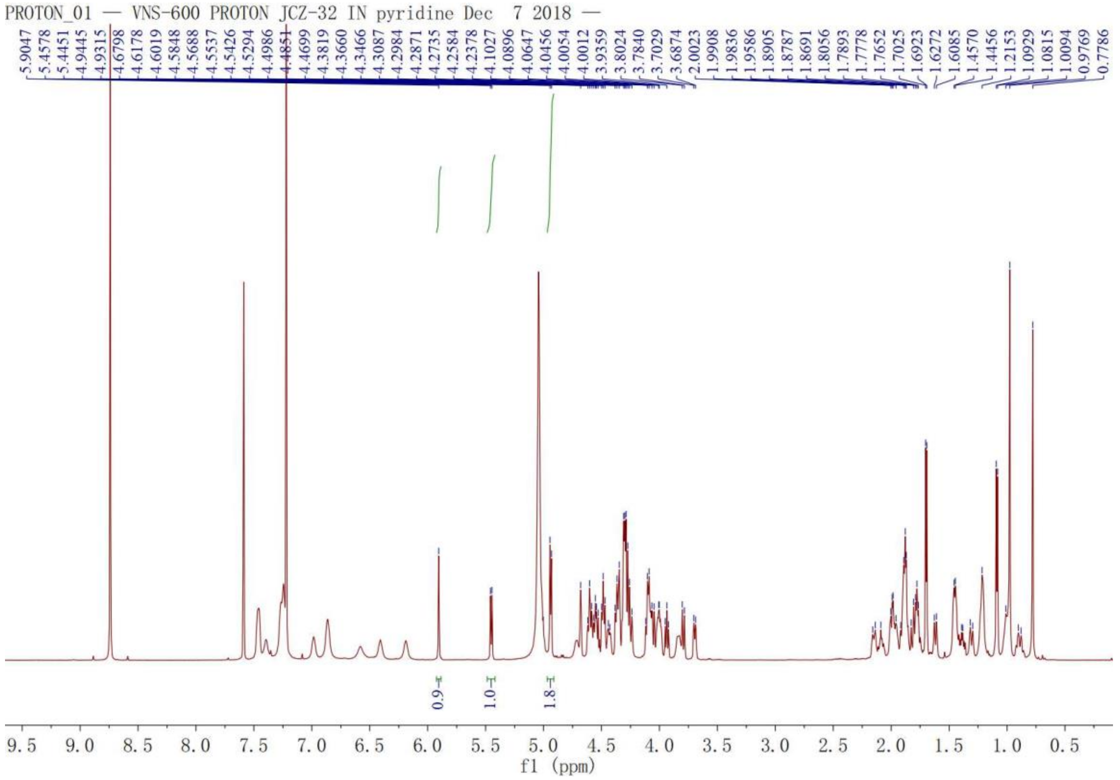

^{1}H NMR spectrum of 23.

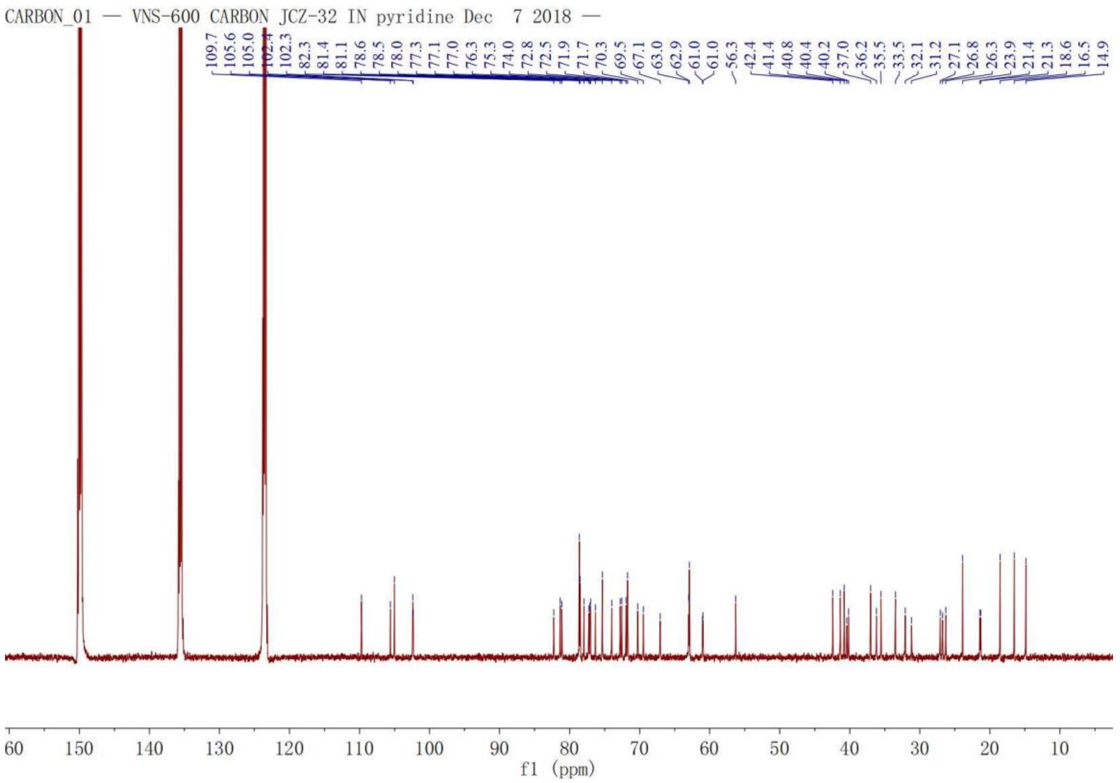

^{13}C NMR spectrum of 23.

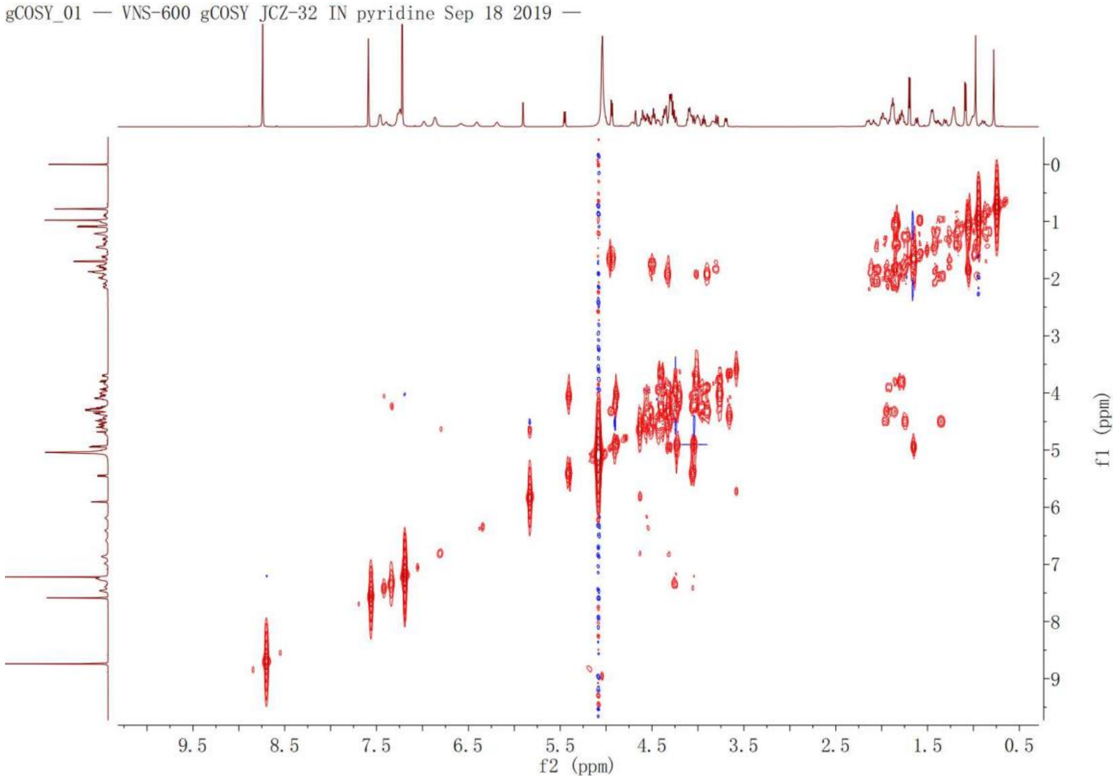

^{1}H-^{1}H COSY spectrum of 23.

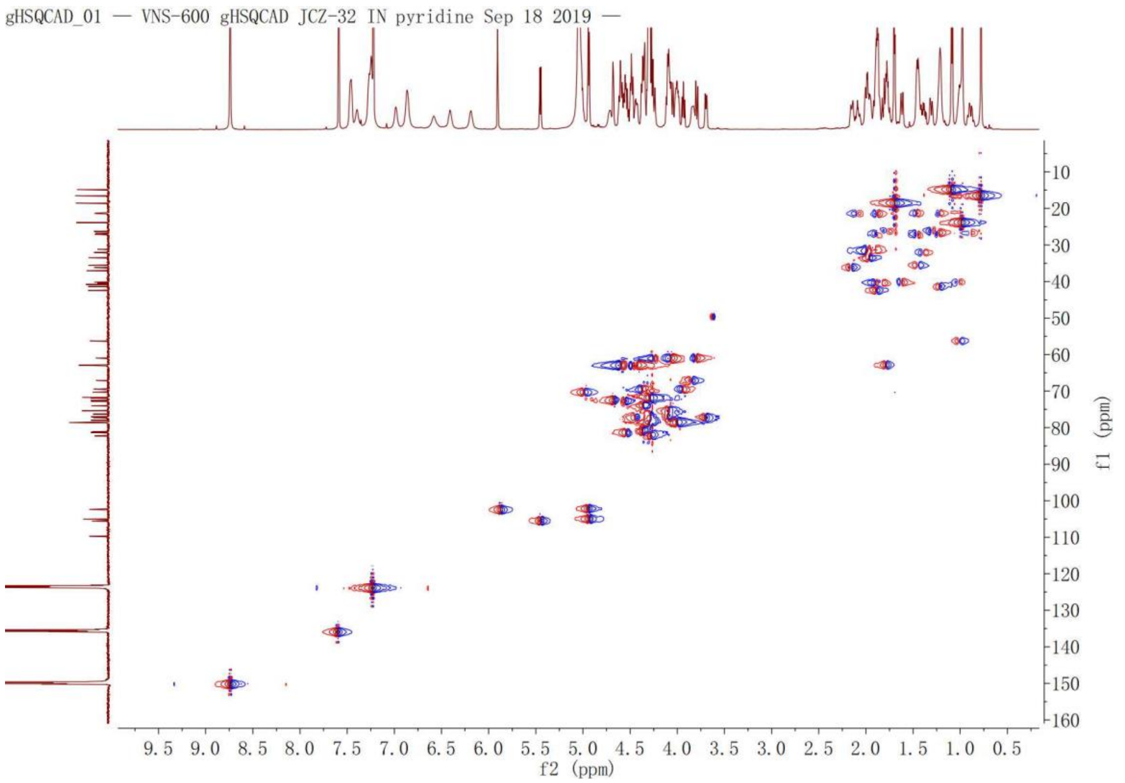

HSQC spectrum of 23.

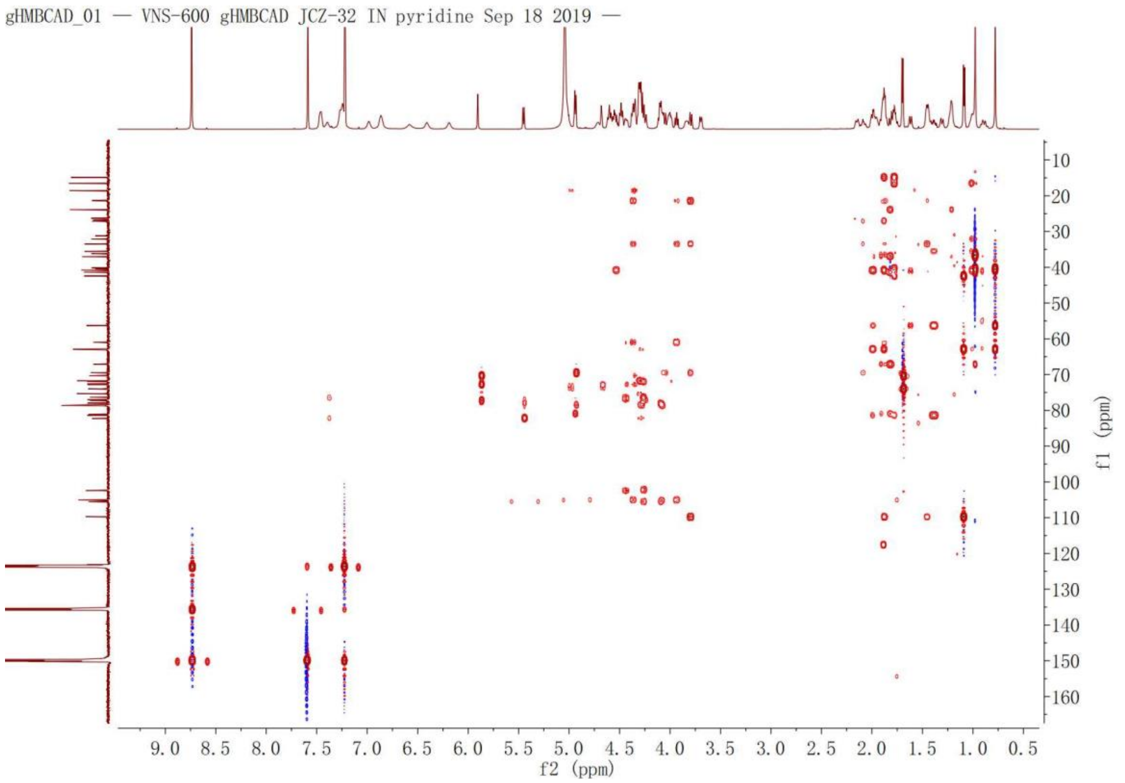

HMBC spectrum of 23.

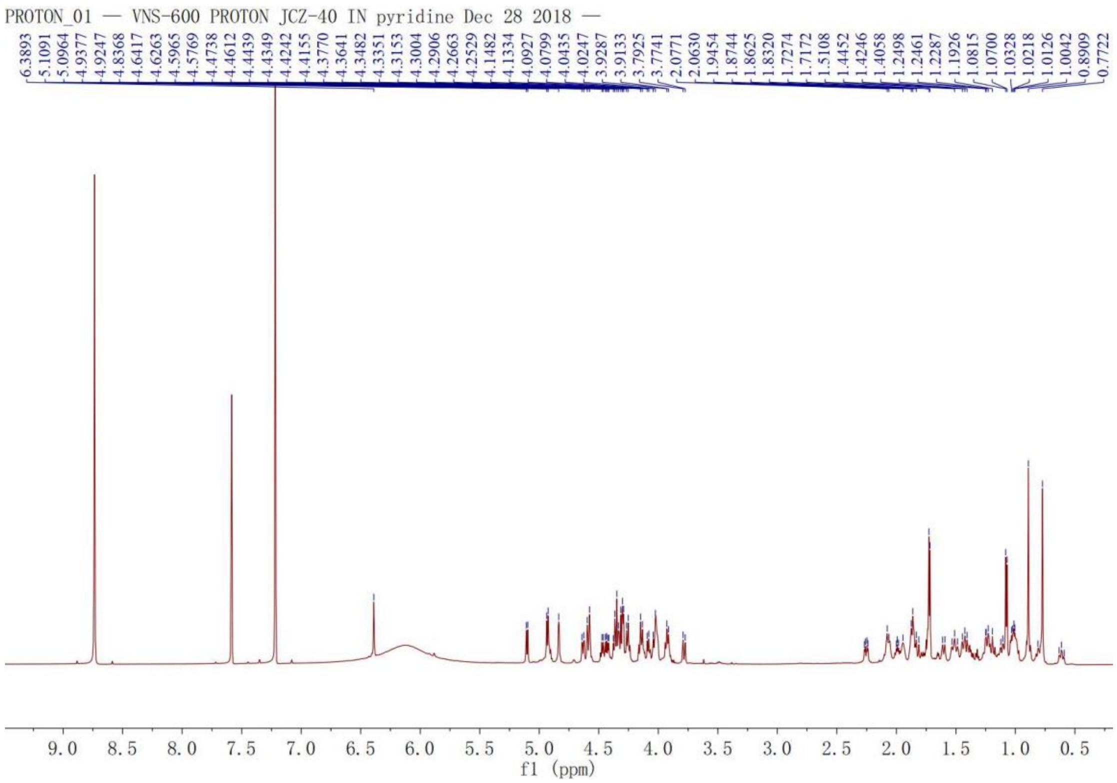

^{1}H NMR spectrum of 24.

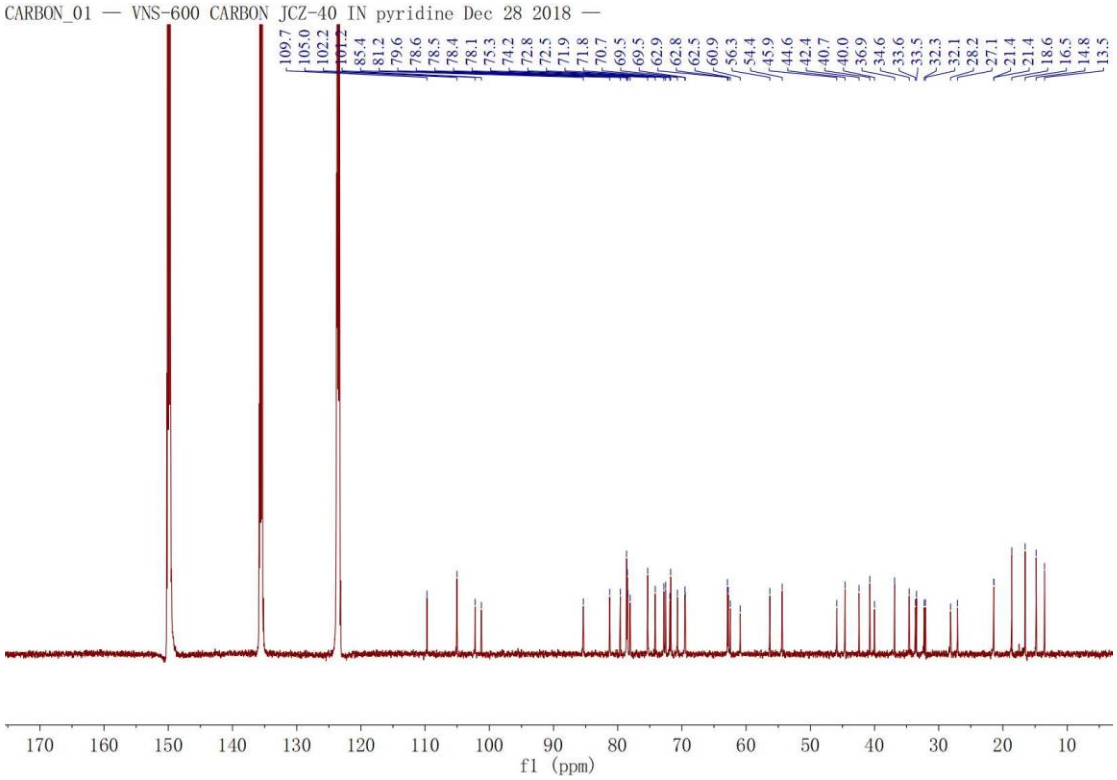

^{13}C NMR spectrum of 24.

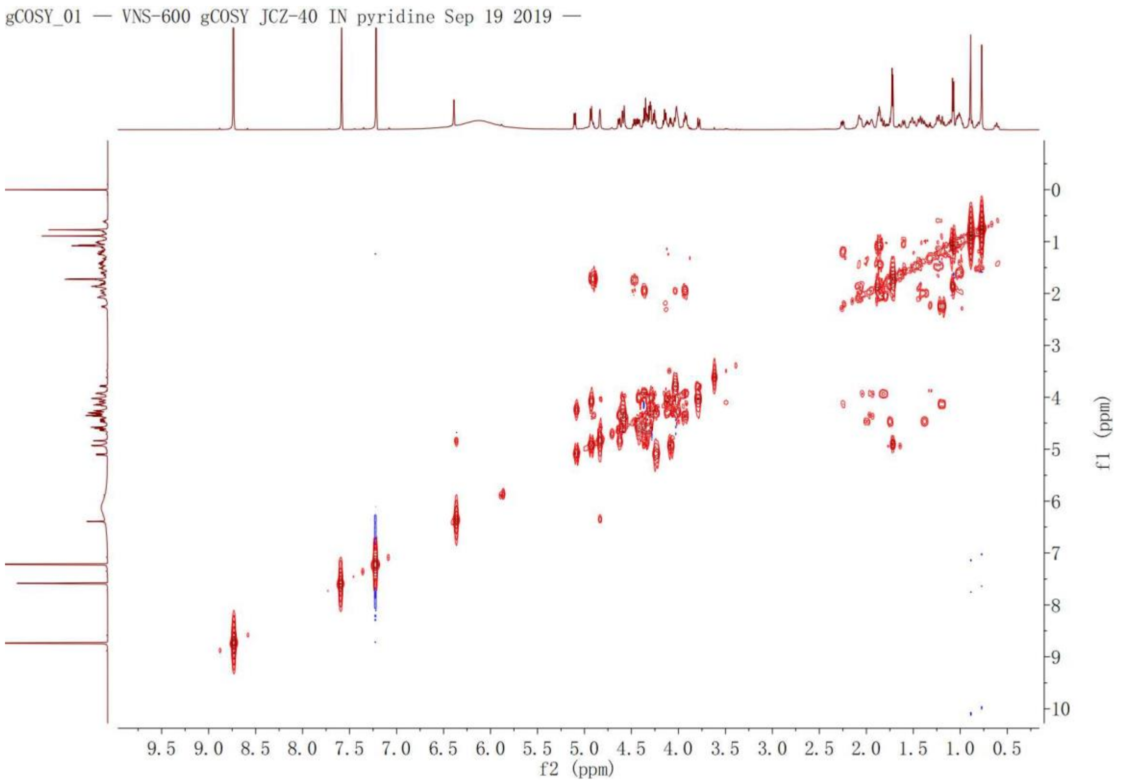

^{1}H-^{1}H COSY spectrum of 24.

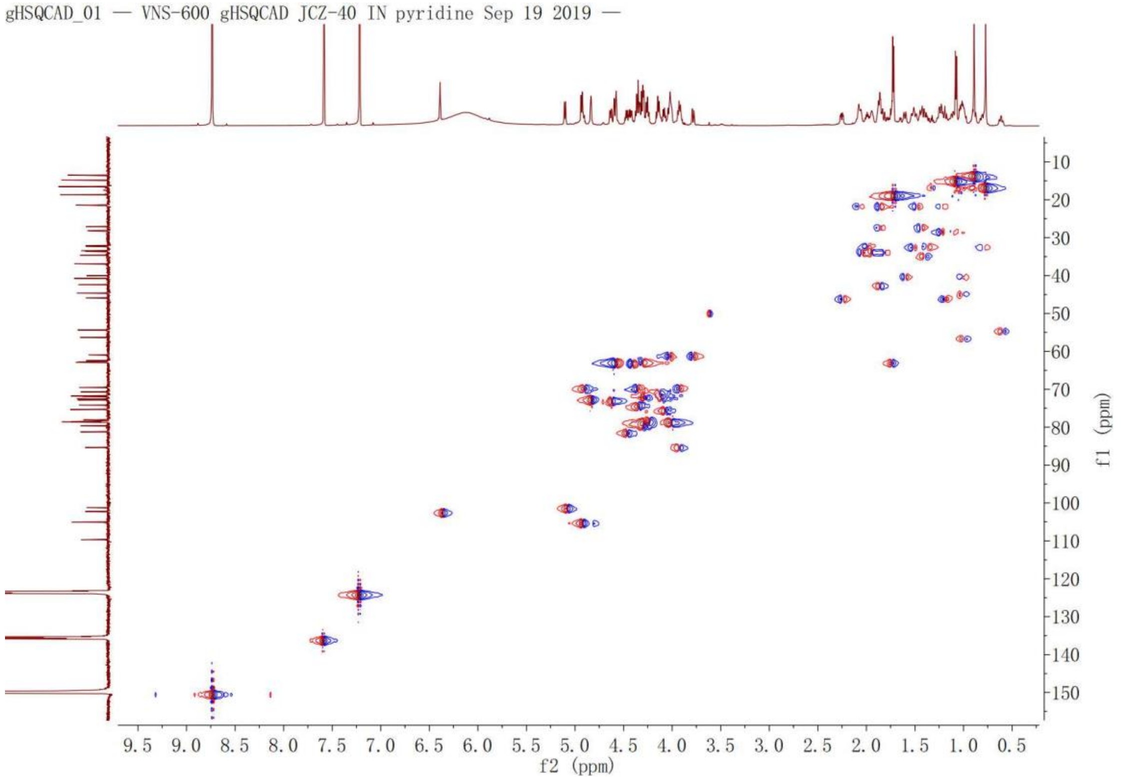

HSQC spectrum of 24.

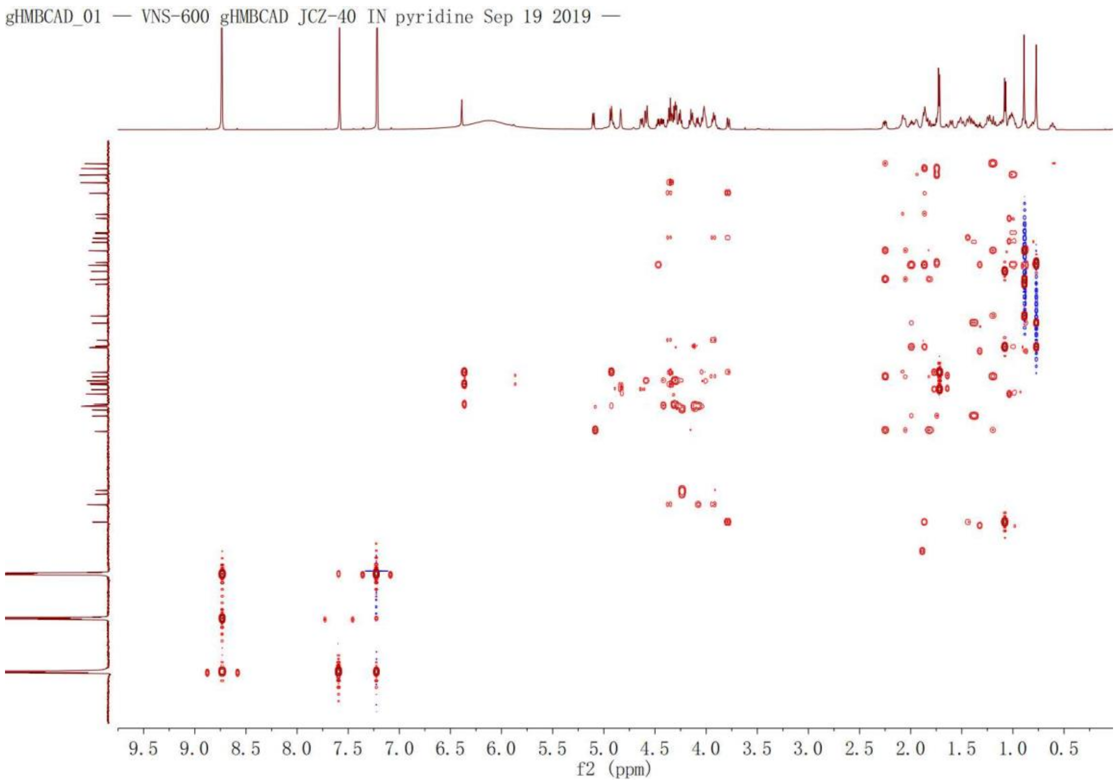

HMBC spectrum of 24.

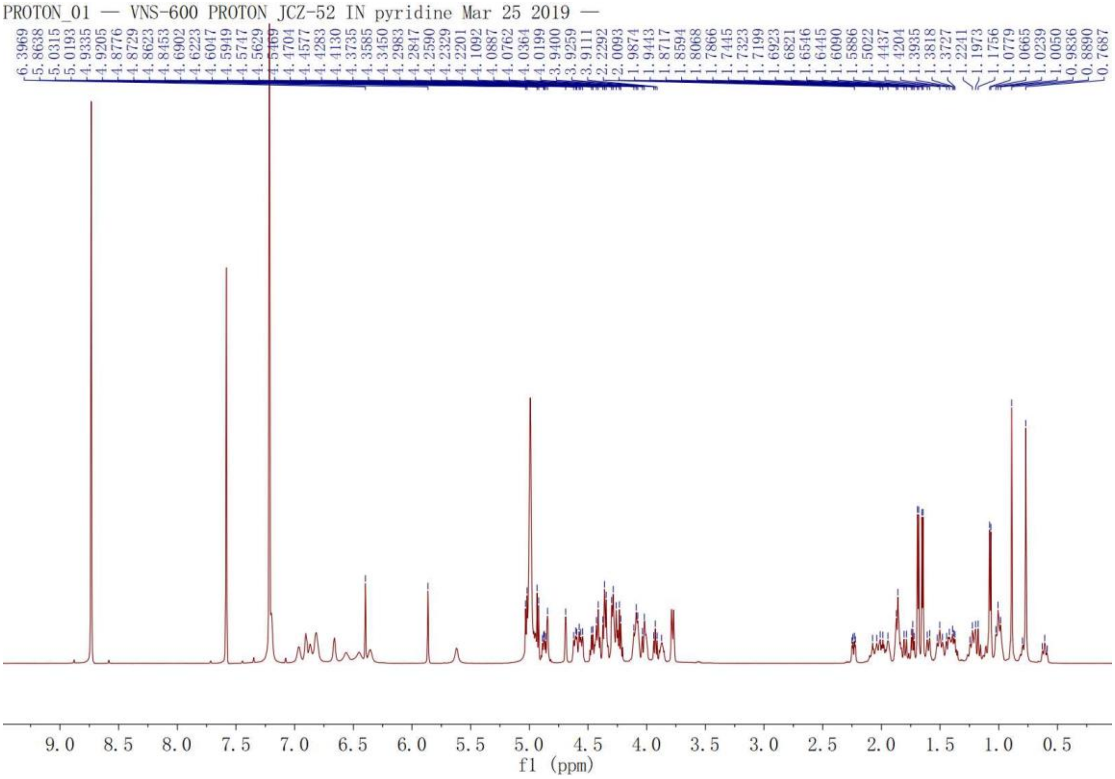

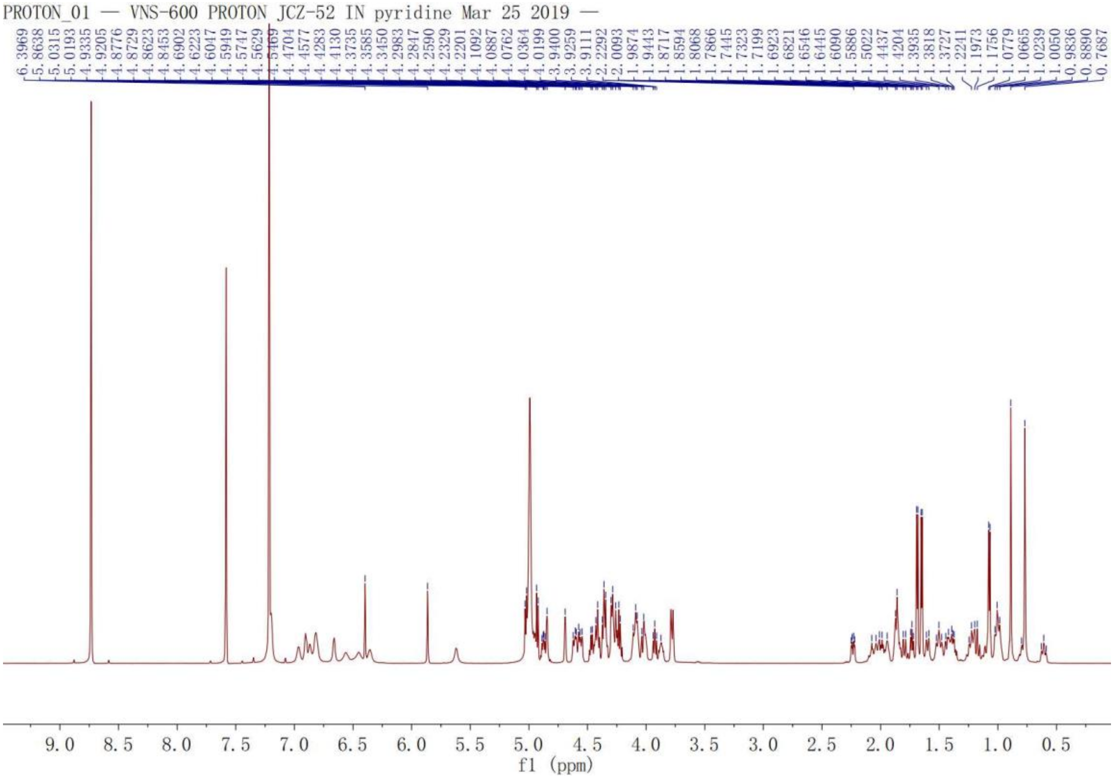

^{1}H NMR spectrum of 25.

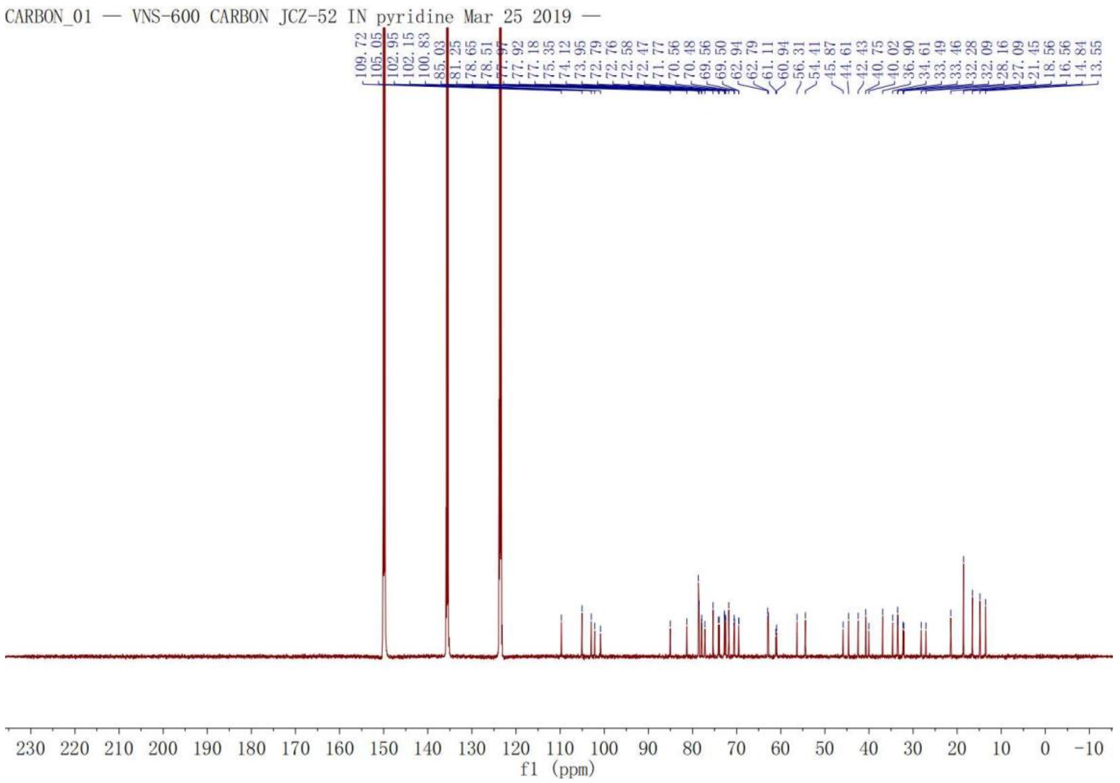

^{13}C NMR spectrum of 25.

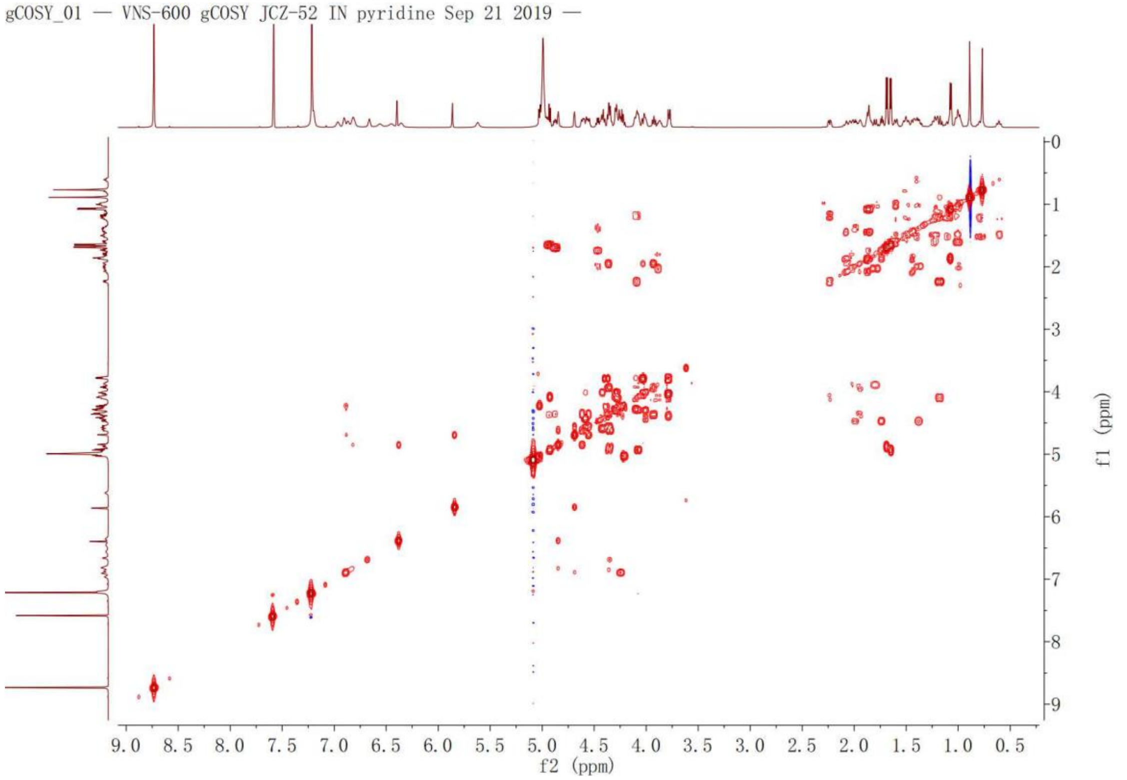

^{1}H-^{1}H COSY spectrum of 25.

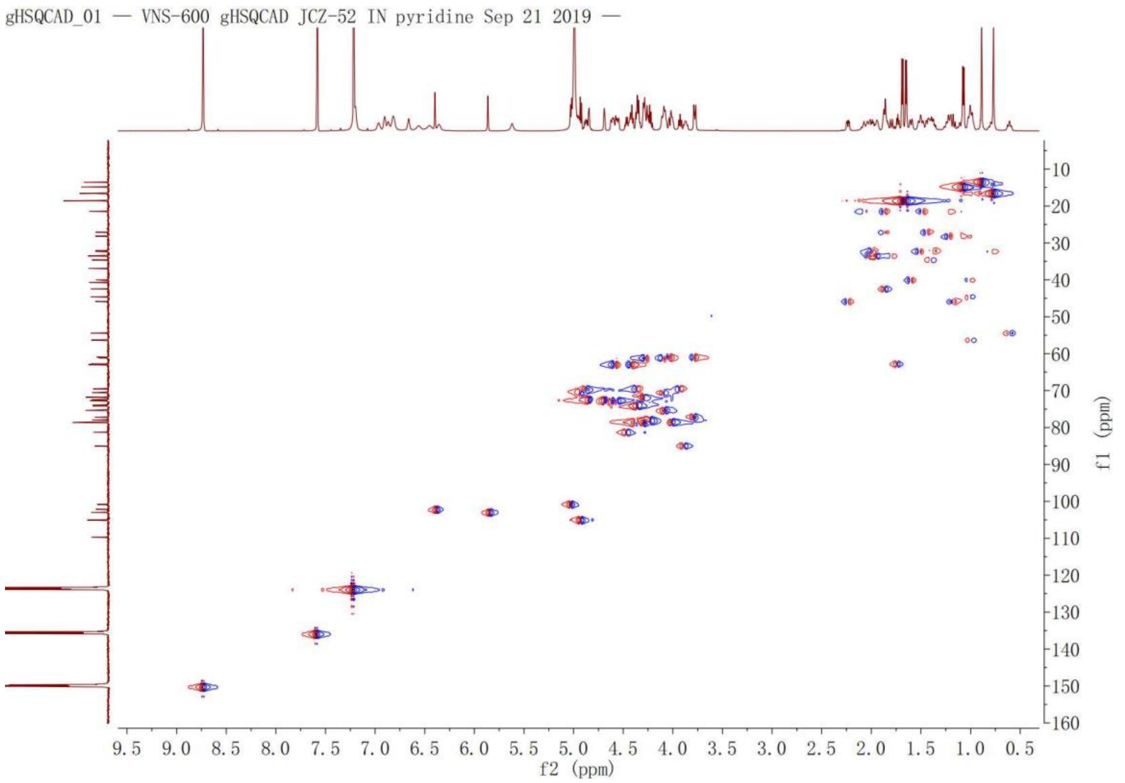

HSQC spectrum of 25.

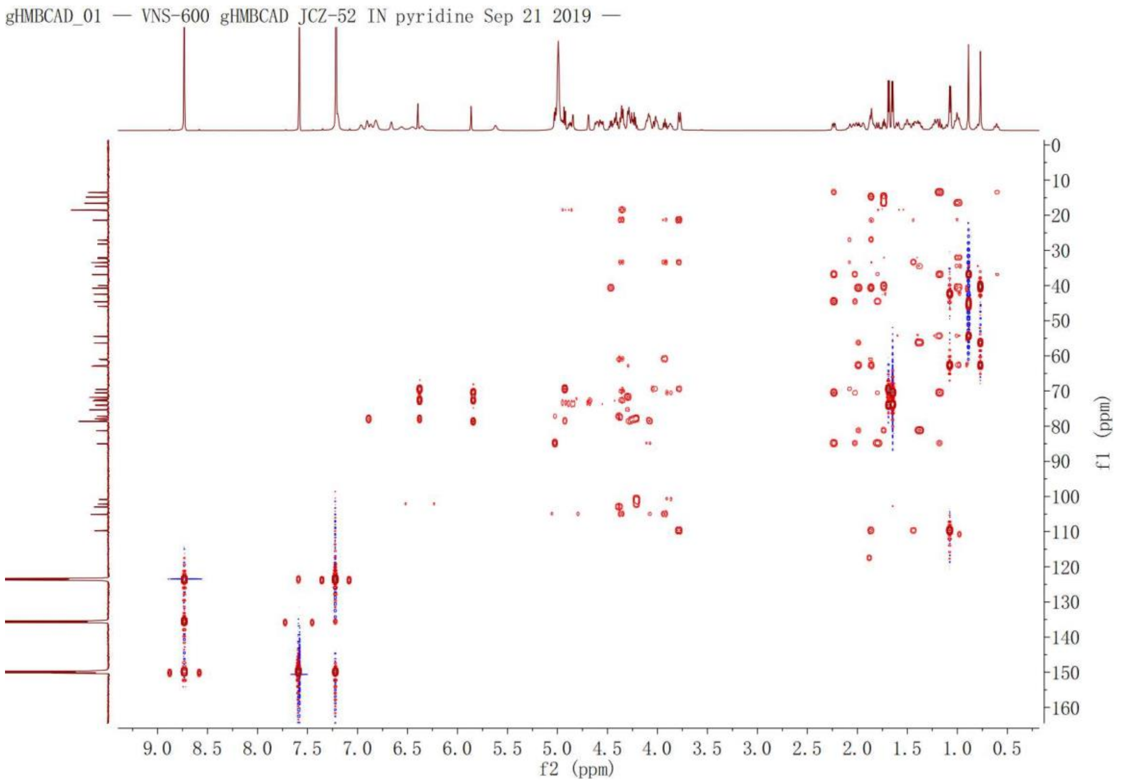

HMBC spectrum of 25.

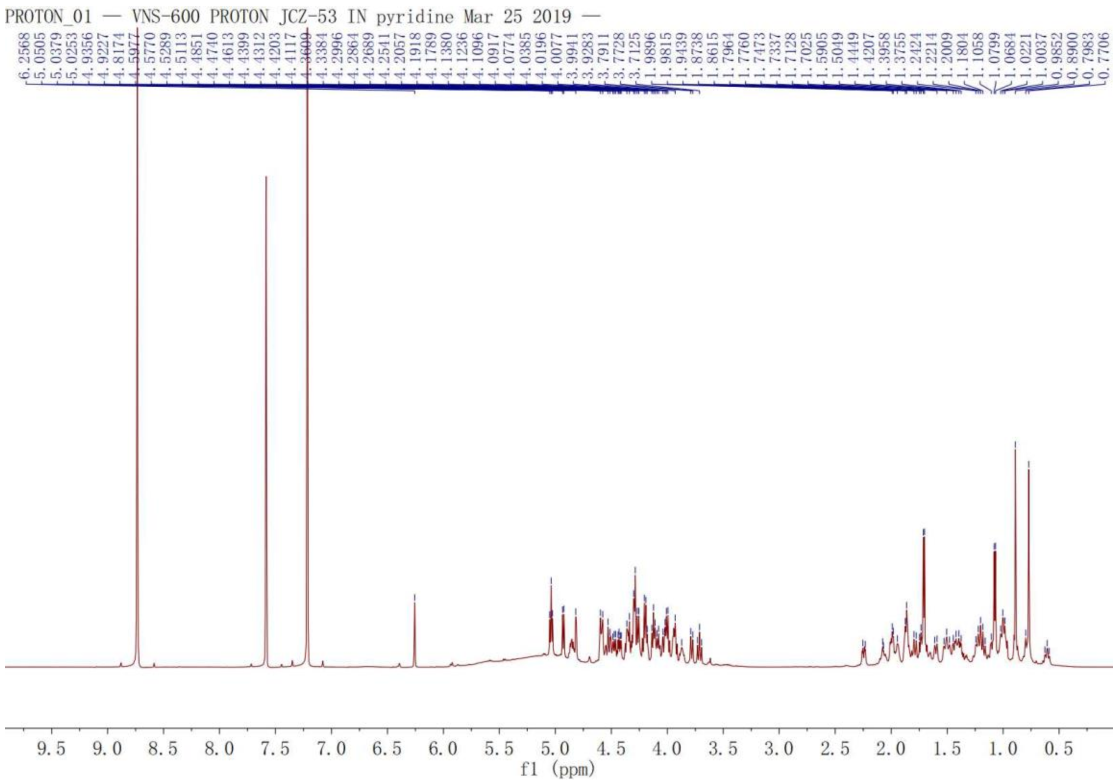

^{1}H NMR spectrum of 26.

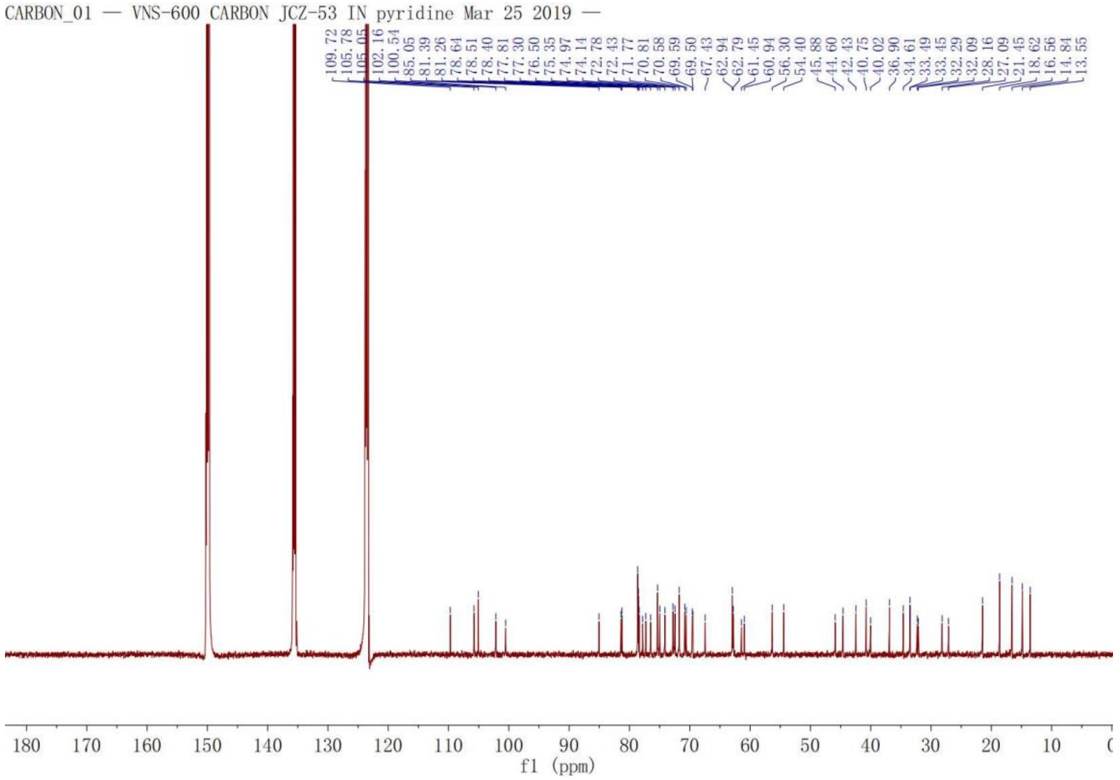

^{13}C NMR spectrum of 26.

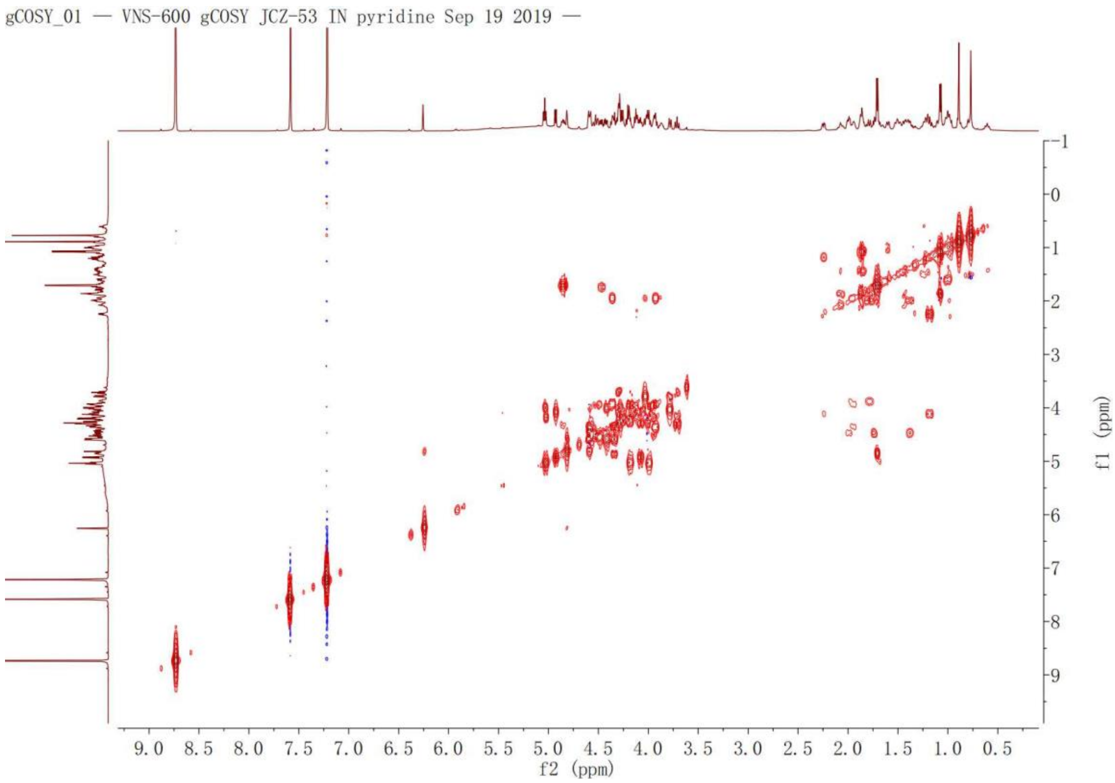

^{1}H-^{1}H COSY spectrum of 26.

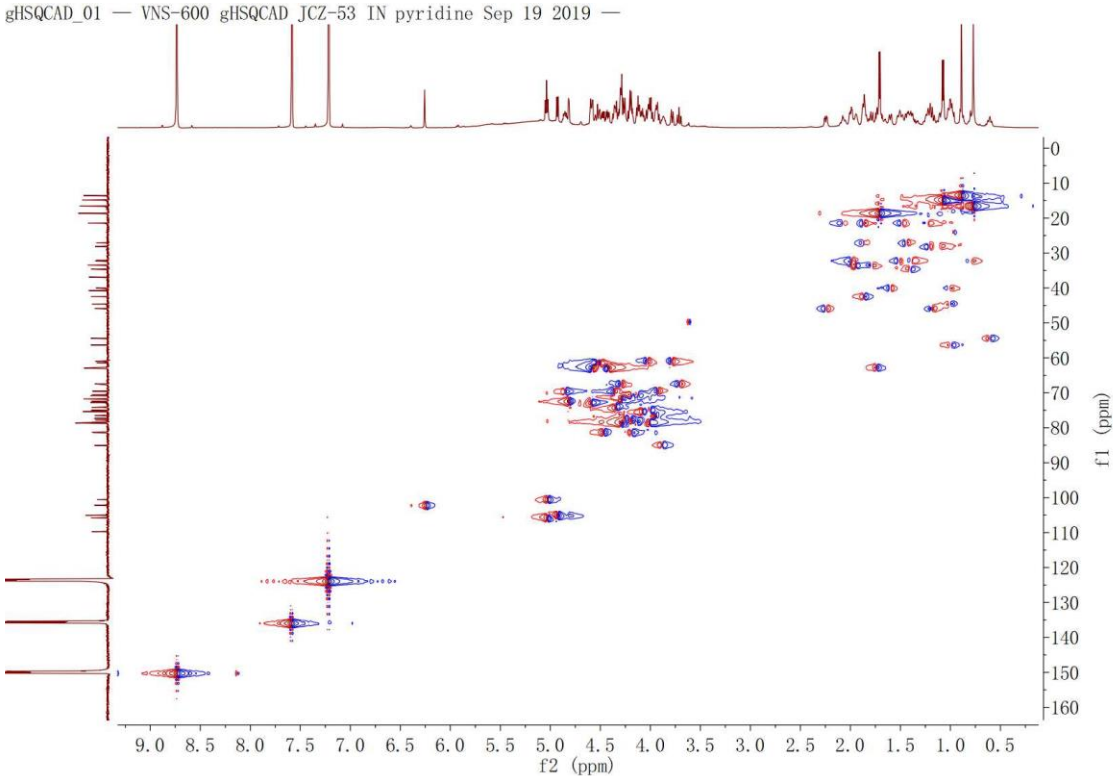

HSQC spectrum of 26.

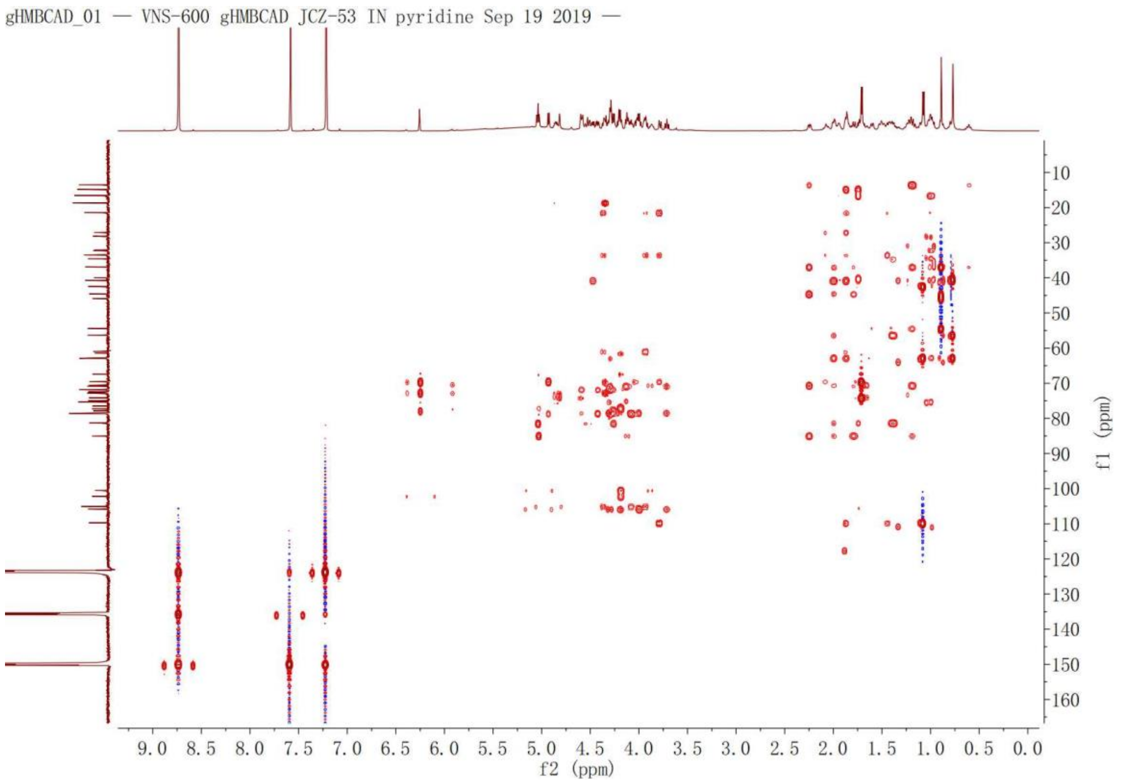

HMBC spectrum of 26.

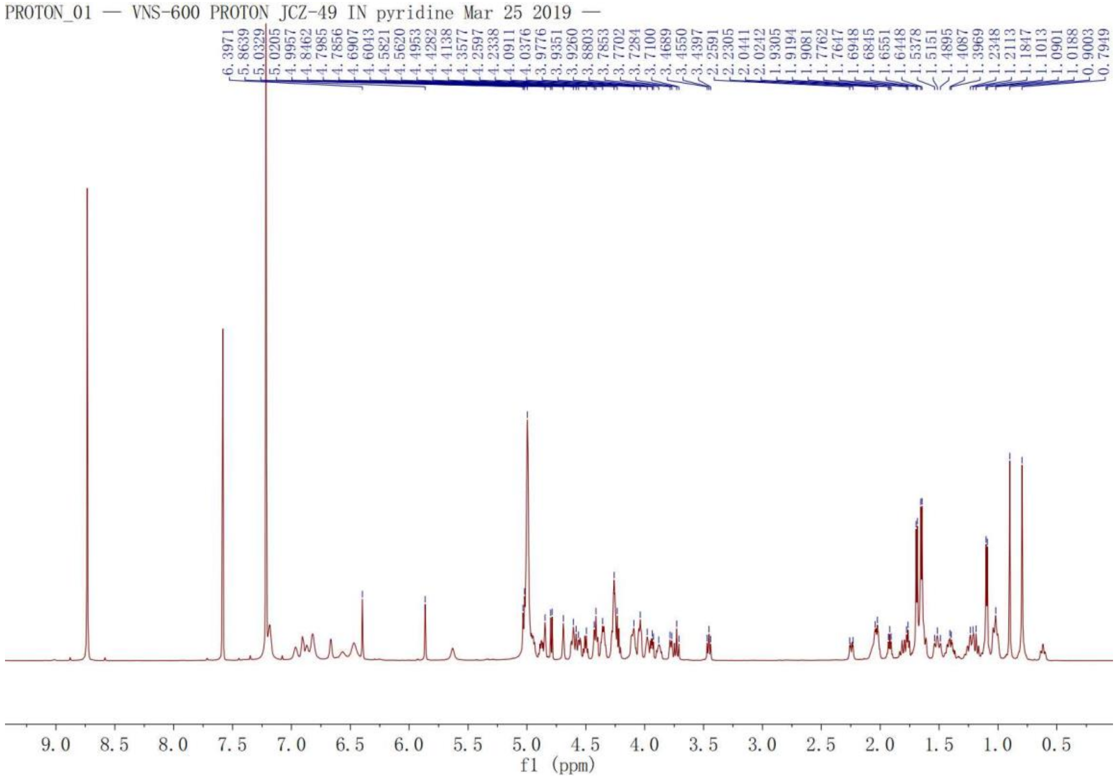

^{1}H NMR spectrum of 27.

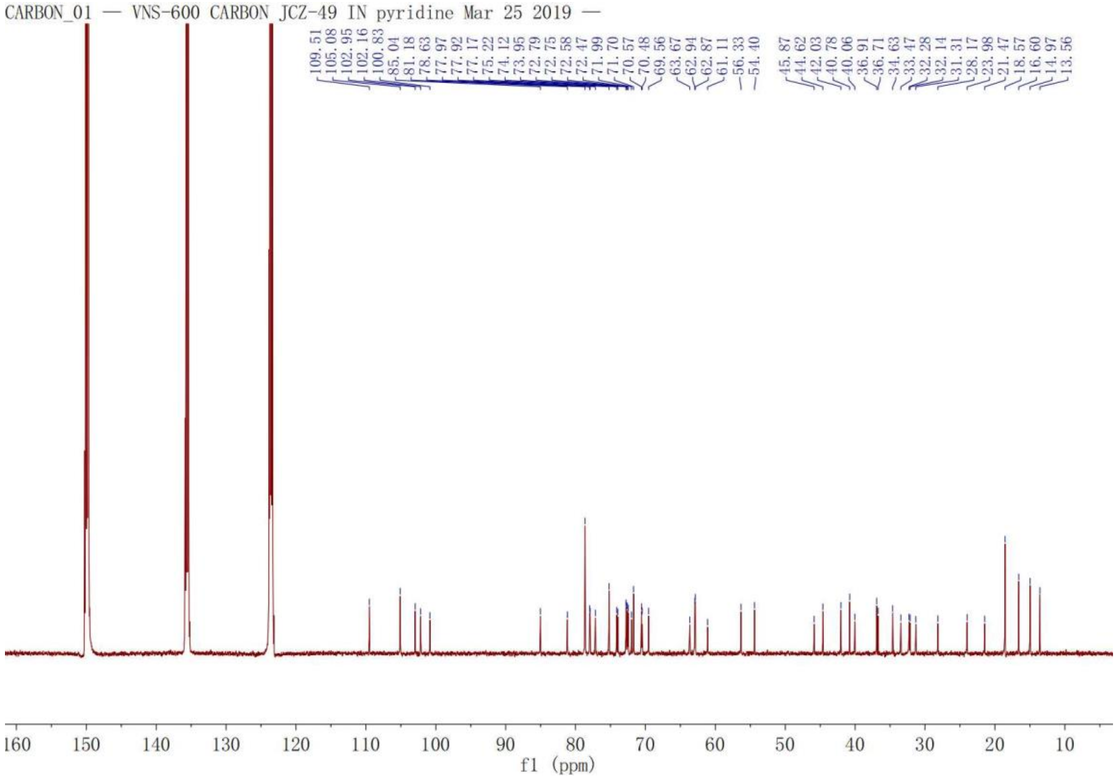

^{13}C NMR spectrum of 27.
